# Identification of soybean *trans*-factors associated with plastid RNA editing sites

**DOI:** 10.1590/1678-4685-GMB-2019-0067

**Published:** 2020-05-11

**Authors:** Nureyev F. Rodrigues, Fábio C. S. Nogueira, Gilberto B. Domont, Rogerio Margis

**Affiliations:** ^1^ Universidade Federal do Rio Grande do Sul (UFRGS), Centro de Biotecnologia, Programa de Pós-Graduação em Biologia Celular e Molecular (PPGBCM), Porto Alegre, RS, Brazil.; ^2^ Universidade Federal do Rio de Janeiro (UFRJ), Instituto de Química, Departamento de Bioquímica, Programa de Pós-Graduação em Bioquímica (PPGBq), Unidade Proteômica, Rio de Janeiro, RJ, Brazil.; ^3^ Universidade Federal do Rio de Janeiro (UFRJ), Instituto de Química, Laboratório de Apoio ao Desenvolvimento Tecnológico (LADETEC), Rio de Janeiro, RJ, Brazil.; ^4^ Universidade Federal do Rio Grande do Sul (UFRGS), Departamento de Biofísica, Porto Alegre, RS, Brazil.

**Keywords:** Chloroplast, Glycine max, PPR, rps14, salt stress

## Abstract

RNA editing is a posttranscriptional process that changes nucleotide sequences, among which cytosine-to-uracil by a deamination reaction can revert non-neutral codon mutations. Pentatricopeptide repeat (PPR) proteins comprise a family of RNA-binding proteins, with members acting as editing *trans*-factors that recognize specific RNA *cis*-elements and perform the deamination reaction. PPR proteins are classified into P and PLS subfamilies. In this work, we have designed RNA biotinylated probes based in soybean plastid RNA editing sites to perform *trans*-factor specific protein isolation. Soybean *cis*-elements from these three different RNA probes show differences in respect to other species. Pulldown samples were submitted to mass spectrometry for protein identification. Among detected proteins, five corresponded to PPR proteins. More than one PPR protein, with distinct functional domains, was pulled down with each one of the RNA probes. Comparison of the soybean PPR proteins to Arabidopsis allowed identification of the closest homologous. Differential gene expression analysis demonstrated that the PPR locus Glyma.02G174500 doubled its expression under salt stress, which correlates with the increase of its potential rps14 editing. The present study represents the first identification of RNA editing *trans*-factors in soybean. Data also indicated that potential multiple *trans*-factors should interact with RNA *cis*-elements to perform the RNA editing.

## Introduction

The evolutionary history of chloroplasts underwent several selective and adaptive processes, particularly along terrestrial colonization. Massive transfers of genetic information to the host genome and its functional assimilation led to retraction in the endosymbiotic genome (Timmis *et al.*, 2004). A strong selective pressure acted to maintain the remaining endosymbiotic genetic information. Posttranscriptional processes were selected by promoting the maintenance of essential sequences for gene expression and functional proteins. In plastids, RNA editing is a nucleotide change from cytosine to uracil (C-to-U) and less frequently, from uracil to cytosine (U-to-C) by deamination and amination reactions, respectively (Chateigner-Boutin and Small, 2010; Takenaka *et al.*, 2013). These changes are necessary for RNA maturation, to generate start or stop codons, or even to result in changes in amino acid identity (Schallenberg-Rüdinger and Knoop, 2016).

Extensive studies have been performed to elucidate molecular features, mechanism, and the machinery of plastid RNA editing. *Cis*-element sequences were identified and reported to be determinant to plastid RNA editing site specificity (Bock *et al.*, 1996). In general, 20 nucleotides upstream and, in some cases, 10 nucleotides downstream from the sequence of the RNA editing site correspond to the *cis*-elements for RNA editing (Vu and Tsukahara, 2017). The first RNA editing *trans*-factor identified was a Pentatricopeptide Repeat protein (PPR). PPR proteins are characterized by tandem arrays of degenerated 31 to 36-amino acid repeating units, called PPR motifs, repeated in tandem up to 30 times, that folds into a pair of antiparallel α-helices, forming a solenoid structure (Small and Peeters, 2000). This protein family has thousands of members in land plants, with about 450 members in Arabidopsis, corresponding to the most studied RNA editing factor so far recognized (Cheng *et al.*, 2016). PPR proteins form sequence-specific associations with RNA, and these associations affect folding, processing, and translation of the RNA, thus manipulating the expression of the transcript (Fujii and Small, 2011). The sequence-specific associations occur through the interaction between protein motifs and RNA, where one motif corresponds to one base, and the amino acids at particular positions determine the nucleotide-binding specificity (Kobayashi *et al.*, 2012).

Plastid RNA editing was reported in most of the plant lineages, and the number of editing sites varies among species. In seed plants, plastid editing sites have already been reported in rice (21), maize (26), tobacco (34), cucumber (51), and *Arabidopsis thaliana* (43) (Ichinose and Sugita, 2016). The identification of editing sites and measurement of editing levels have demonstrated differences among tissues and developmental stages (Miyata and Sugita, 2004; Tseng *et al.*, 2013). These findings can be used to evaluate the impact of different stresses on editing mechanisms. Soybean is a model crop with some prior studies about plastid RNA editing. Our group has described 43 phylogenetically conserved and five non-conserved editing sites in *Glycine max* using RNA sequencing data (Rodrigues *et al.*, 2017a). Besides that, we also have described a salt stress effect in soybean plastid RNA editing (Rodrigues *et al.*, 2017b).

Based on these sequencing data, three plastid RNA editing *cis*-elements were selected, all of them presenting high editing levels, where intense plastid RNA editing *trans-*factors activity is expected. Biotinylated probes were designed based on these *cis*-element sequences to perform an RNA-pulldown protein purification. Plastid RNA editing *trans-*factors acting in selected soybean plastid *cis*-element were identified, and its specificity among sites was evaluated. Also, other proteins were identified that have non-specific *cis*-element binding activity.

## Materials and Methods

### RNA probe design for *cis-*elements

The soybean chloroplast genome was retrieved from NC_007942.1 accession. The coding sequences of *atpF* (GlmaCp025), *ndhB* (GlmaCp064), and *rps14* (GlmaCp013) genes were used to design RNA probes. Three probes were produced corresponding to *atpF*-92, *ndhB*-1481, and *rps14*-80 editing sites as the reference to select 28 upstream and 7 downstream nucleotides, totalizing a 36-nucleotide probe from each editing site: *atpF*-92, UUUAAUACCGAUAUUUUAGCAACAAAUCCAAUAAAU; *ndhB*-1481, AUUGUAUGUGUGAUAGCAUCUACUAUACCAGGAAUA; and *rps14*-80, CAGAAAUAUCAUUUGAUUCGCCGAUCCUCAAAAAAA. The RNA probes were synthesized and biotinylated at the 5’ end. To analyze the conservation of RNA *cis*-element sequences among species, chloroplast coding sequences for each transcript were identified in the eight species listed in Table S1. A tree was created using the Neighbor-Joining method, with p-distance model performed in the Molecular Evolutionary Genetics Analysis (MEGA) 6.0 software (Tamura *et al.*, 2013). Sequence logos were generated using WebLogo3 (Crooks, 2004) at http://weblogo.threeplusone.com.

### Plant material and chloroplast isolation

For chloroplast isolation, soybean (*Glycine max* (L.) Merrill) cultivars Conquista were cultivated until the fifth trifoliate (V5) stage. The modified high salt chloroplast isolation protocol was followed to obtain chloroplasts (Vieira *et al.*, 2014).

### Plastid protein extraction and protein isolation by RNA probe pulldown

All the following steps were carried out at 0 °C if not otherwise stated. The final chloroplast pellet was resuspended in lysis buffer (0.2 M potassium acetate, 30 mM Tris-HCl pH 8.0, 10 mM MgCl_2_, 2 mM DTT) and transferred to a microcentrifuge tube. The resuspended solution was pulled through a syringe (0.3 mm 8 mm) 60 times. The homogenate was centrifuged twice at 16,000 x *g* for 20 min at 4 °C. A supernatant aliquot was transferred to a new tube, and the same volume of incubation buffer (150 mM NaCl, 20 mM Tris-HCl pH 8.0, 1 mM EDTA, 5 mM MgCl2, 0.5% Triton X-100) was added. The homogenate was transferred to a new tube and biotinylated probes (final concentration 5 μM) corresponding to each editing site were added. The solution was incubated at 160 rpm for 30 min at 25 °C. Control blank analyses corresponded to resin incubated with total protein extracts without any RNA probe. In addition to the blank control, each probe can be considered and used as the control of each other in the protein identification assays, forming a group in the analyses. The homogenate was transferred to a centrifuge tube containing streptavidin-agarose resin previously washed with lysis and incubation buffer 1:1 (v/v) thrice. The washing step consisted of adding the solution, gentle manual shaking and resin decantation, followed by discarding the volume above the resin. The solution was maintained on a gentle manual shaking for 15 min. Two washing steps were performed with lysis and incubation buffer 1:1 (v/v), followed by three washing steps with lysis and incubation buffer (without Triton X-100) 1:1 (v/v). The final solution containing streptavidin-agarose resin, biotinylated probes/blank control, and plastid proteins was maintained at -20 °C before sample preparation.

### Sample preparation for proteomic analysis

The resins were incubated for 5 min at room temperature, with 7 M urea/2 M thiourea. Proteins extracted from resins were further reduced using 10 mM DTT for 60 min at 35 °C and alkylated using 40 mM iodoacetamide for 60 min at 35 °C in the dark. Urea concentration was diluted to less than 1 M using 50 mM NH_4_HCO_3_ pH 8.0 and proteins were digested with trypsin (Promega) overnight at 35 °C. Trifluoroacetic acid (TFA) was added (final concentration 0.1%) in order to stop digestion, and peptides were passed through C18 spin columns (Harvard Apparatus), dried under vacuum and stored at -20 °C for further use. Two biological replicates were subjected to digestion for each RNA probe.

### Protein identification by mass spectrometry

Peptides obtained from the tryptic digestion (2 μg) were loaded onto a C18 reversed-phase pre-column (2 cm long, 100 μm internal diameter, with ReproSil-Pur C18-AQ 5 μm beads - Dr. Maisch GmbH) and fractionated on a New Objective PicoFrit^®^ Self-Pack column (18 cm long, 75 μm internal diameter, with ReproSil-Pur C18-AQ 3 μm beads - Dr. Maisch GmbH). The samples were analyzed in an EASY-nLC II system (Proxeon Biosystems) coupled in sequence to a high-resolution ESI-LTQ-Orbitrap Velos mass spectrometer (Thermo Scientific). The peptides were eluted using the gradient starting from 100% phase A (0.1% formic acid, 5% acetonitrile) to 35% phase B (0.1% formic acid, 95% acetonitrile) for 107 minutes, 35-100% of phase B for 5 min, and 100% of phase B for 8 min, totaling 120 min in a flow of 250 mL/min. After each run, the column was washed with 100% of phase B and re-equilibrated with phase A. 

The m/z spectra were obtained in positive mode with data-dependent automatic acquisition - Data-Dependent Acquisition (DDA) - of the MS and MS/MS spectra. The MS spectra were obtained in high resolution in the Orbitrap analyzer with a resolution from 30,000 at m/z 400, mass range of m/z 350-2000, Automatic Gain Control (AGC) of 1 x 106 and maximum injection time of 500 MS. The MS/MS spectra were obtained by higher energy collisional dissociation (HCD) in the Orbitrap for the 10 most intense ions, with a charge ≥ 2, resolution of 7500 at m/z 400, signal threshold of 10,000, the normalized energy of collision (NCE) of 30, and dynamic exclusion of 45 s. Proteome Discoverer 2.1 software was used for data analysis applying the Sequest^TM^ algorithm and a *G. max* database downloaded from Phytozome (June 2017). The parameters used were: full-tryptic search space, up to two missed cleavages allowed for trypsin, precursor mass tolerance of 10 ppm, and fragment mass tolerance of 0.1 Da. Carbamidomethylation of cysteine was included as fixed modification, and methionine oxidation and protein N-terminal acetylation as dynamic modifications.

### Analysis of probe-PPR protein binding events

To determine the specificity of the interaction between selected PPR and the respective probe sequence, the aPPRove method (Harrison *et al.*, 2016) was used to evaluate how and where the PPR protein binds to the RNA designed probes, and if this binding event has a statistical significance. The sequences from the PPR proteins and the RNA probes were used as input. The chloroplast genome sequence of soybean was used as information for random alignment. Binding events that had high statistical significance (*p* < 0.05) were selected.

### Phylogenetic analysis of *trans*-acting editing factors

Complete protein sequences from pulled-down PPR proteins were retrieved from the Phytozome database. These sequences were used as queries in BLASTP searches with default parameters against the Phytozome database to retrieve other Arabidopsis and soybean PPR proteins. To determine the structural organization and motif/domain composition of the *trans* -factors, the sequences were submitted to the Pfam web server (http://pfam.xfam.org/) for the prediction of functional domains (Finn *et al.*, 2016). The sequence domain found in each protein sequence was retrieved to create a fasta file. The protein domain sequences were aligned using MUSCLE (Edgar, 2004). The multiple alignments were manually inspected using Molecular Evolutionary Genetics Analysis (MEGA) 6.0 software (Tamura *et al.*, 2013). The model of protein evolution for each protein matrix substitution was calculated from multiple alignments by ProtTest3 (Darriba *et al.* , 2011). The phylogenetic tree was constructed using the Bayesian method, performed in BEAST 1.8.4 software (Drummond and Rambaut, 2007). The Birth/Death tree was selected as a tree prior to Bayesian analysis and 20,000,000 generations were performed with Markov chain Monte Carlo (MCMC) algorithms. The tree was visualized and edited using FigTree v1.4.3 software (http://tree.bio.ed.ac.uk/software/fig-tree/).

### Differential gene expression

 Public mRNAs libraries of soybean leaves, deposited by our group in NCBI GEO (http://www.ncbi.nlm.nih.gov/geo/), accession number GSE69571, were used to evaluate the differential gene expression of the identified PPR proteins. SAM files were created using Bowtie software (Langmead *et al.*, 2009) with default parameters and zero mismatches. A count table containing data from all libraries was created and used as an input file for differential expression analysis performed using the Bioconductor DESeq2 package (Love *et al.*, 2014) with an adjusted *p*-value cutoff of 0.05.

## Results

### Conservation of editing sites *cis*-elements

**Figure 1:**
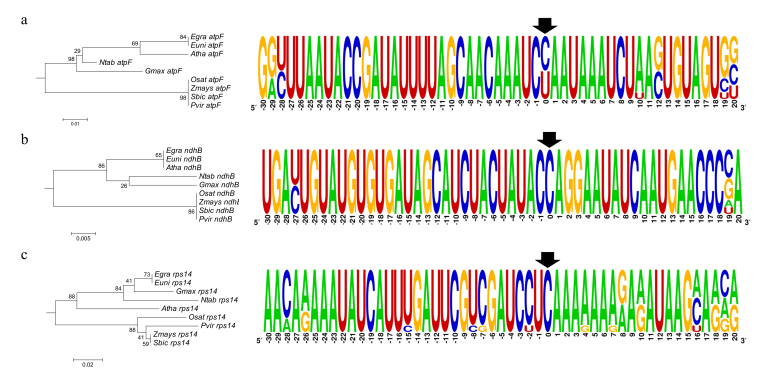
Sequence analysis of *cis*-elements. A neighbor-joining tree was created using the p-distance method and the sequence alignment of the region surrounding the (a) *atpF-92*, (b) *ndhB*-1481, and (c) *rps14*-80 editing sites, from -30 to +20 around the edited C (position zero) of *A. thaliana (Atha), E. uniflora (Euni), G. max (Gmax), N. tabacum (Ntab), O. sativa (Osat), P. virgatum (Pvir), S. bicolor (Sbic)*, and *Z. mays (Zmay).* A consensus logo is shown for each one of the three alignments, with an arrow indicating the editing nucleotide.

Recognition sequences from *atpF*-92, *ndhB*-1481, and *rps14*-80 editing sites were analyzed at 30 upstream and 20 downstream nucleotides in eight species (Figure 1). The *atpF-92* sequence conservation is divided between monocots and dicots (Figure 1a). Monocots already have thymine in the editing site location (Figure S1a). Other differences occur after 26 upstream and 10 downstream nucleotides. The *ndhB*-1481 recognition sequence is the most conserved among all analyzed recognition sequences. Differences could be observed only in position 27 upstream and 19 downstream from an editing site (Figure 1b and Figure S1b). The *rps14*-80 recognition sequence is the most variable sequence among all analyzed ones. Differences could be observed even within monocots (Figure 1c). In total, 14 positions with nucleotide differences were observed in the *rps14*-80 recognition sequence (Figure S1c).

### Non-specific protein profile

Despite sequence differences in the designed RNA probes, several non-specific proteins could be identified by the RNA probes used in the pulldown. The elution profile using *atpF*-92, *ndhB*-1481, and *rps14*-80 probes comprises 83, 106, and 78 proteins respectively, while the blank profile, corresponding to a sample not incubated with RNA-probes, comprises 160 proteins (Table S2).

Different RNA binding proteins were identified in the three distinct RNA probe pulldown profiles (Table 1). These proteins are involved in RNA metabolism and the translation process. Two RNA helicases were identified in the protein profiles of the RNA probe pulldown (Glyma.02G119000 and Glyma.18G014800) and two translation initiator factors IF-2 (Glyma.08G174200 and Glyma.19G044300). Other plastid proteins that are not RNA-binding were also identified: light-harvesting complex II chlorophyll a/b binding protein 1, LHCB1 (Glyma.16G165200), CHLOROPLAST UNUSUAL POSITIONING1, CHUP1 protein (Glyma.20G185300), weak chloroplast movement under blue light, WEB1 protein (Glyma.18G021300 and Glyma.08G266500) and magnesium chelatase subunit H (Glyma.10G097800). The cytosolic translation and transcription factors, kinases, metabolic enzymes and, in lesser abundance, cytoskeleton components were the main non-plastid contaminations in the RNA probe pulldown.

**Table 1:** RNA-interacting proteins identified in mass spectrometry assays and their respective probes.

Protein	Accession	RNA probe
Pentatricopeptide repeat proteins		
PPR	Glyma.11G217500	*atpF-92*
PPR	Glyma.19G025700	*atpF-92*
PPR	Glyma.01G016100	*ndhB*-1481
PPR	Glyma.11G111200	*ndhB*-1481*, rps14*-80
PPR	Glyma.02G174500	*rps14*-80
RNA helicases		
DEAD/DEAH box helicase	Glyma.02G119000	*atpF-92*, *ndhB*-1481, *rps14*-80
Helicase, IBR and zinc finger protein domain-containing protein	Glyma.18G014800	*rps14*-80
Translation factors		
Initiation factor (IF-2)	Glyma.08G174200	*atpF-92*
Initiation factor eIF-2B subunit delta (EIF2B4)	Glyma.19G044300	*ndhB*-1481

### Pentatricopeptide repeat proteins (PPR) identified by pulldown

In total, five PPR proteins were identified in different RNA probe pulldown profiles (Table 1). Glyma.11G217500 and Glyma.19G025700 proteins were identified in the *atpF-92* pulldown profile. These proteins have two Pfam domains assigned as PPRs: PF01535 and PF13041, six copies of PF01535, and a single PF13812, respectively. Glyma.19G025700 differs from the first PPR protein by harboring a third domain corresponding to a cytosine-deaminase (PF14432) that presents a DYW motif.

**Figure 2:**
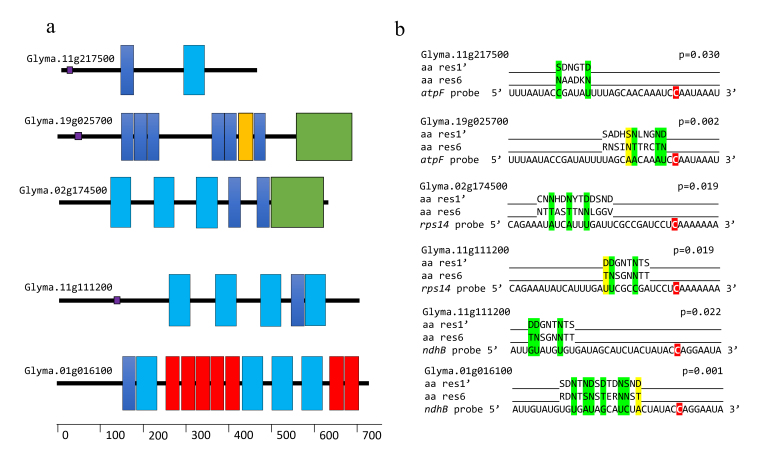
Protein structure of the pulled-down PPR proteins and their probe alignments using the aPPRove method. (a) The protein structures designed based in Pfam prediction. The different colors correspond to four PPR Pfam domains: blue (PF01535), red (PF12854), light blue (PF13041), yellow (PF13812), and to cytosine-deaminase Pfam domains in green (PF14432). (b) aPPRove prediction of the 6 and 1’ amino acids alignments of PPR protein to the RNA probe sequence. Green and yellow indicate, respectively, higher and lower frequency alignment predicted by aPPRove. Marked in red are the RNA editing site locations.

Two others PPR proteins were associated with the *rps14*-80 probe. Glyma.02G174500 with two PPR domains (three copies of PF01535 and two PF13041) plus the cytosine-deaminase domain with the DYW motif (PF14432). The second PPR protein, Glyma.11G111200 has only two PPR domains (four copies of PF01535 and a single PF13041). This protein was also identified in the *ndhB*-1481 pulldown, as was also observed with Glyma.01G016100 that contains three PPR domains (a single PF01535, seven PF12854, and four PF13041) (Figure 2a). 

The specificity of the PPR-probe alignment was evaluated using the aPPRove method (Harrison *et al.*, 2016). This analysis provides an evaluation of the binding event between the PPR and the probe as not occurring at random. All PPR proteins had more than one alignment per probe. The best alignment for each PPR protein in its respective probe is shown in Figure 2b. All PPR-probe alignments to each PPR protein are listed in Material S1.

The Glyma.19G025700 alignment occurs at one nucleotide upstream of the editing site; three alignments correspond to higher frequency alignment, and one to lower frequency alignment. The Glyma.02G174500 alignment occurs at 10 nucleotides upstream of the editing site, and all three alignments correspond to a higher frequency alignment. Glyma.11G111200 aligns to two different RNA probes; in the *rps*14 probe, the alignment occurs at four nucleotides upstream of the editing site, and in the *ndhB* probe, the alignment occurs at 10 nucleotides upstream of the editing site. Among amino acids/nucleotides combinations, three could be observed; three alignments corresponded to higher frequency alignment and one to lower frequency alignment to the *rps*14 probe, and two higher frequency alignments and one lower frequency alignment were to the *ndhB* probe. The Glyma.11G217500 alignment occurs at 14 nucleotides upstream of the editing site and has only two higher frequency combinations aligned to probe sequence. The Glyma.01G016100 alignment occurs at six nucleotides upstream of the editing site. Among amino acids combinations aligned to nucleotides in a probe sequence, seven corresponded to alignment with higher frequency based in Arabidopsis.

### Homology among Arabidopsis and soybean PPRs

**Figure 3:**
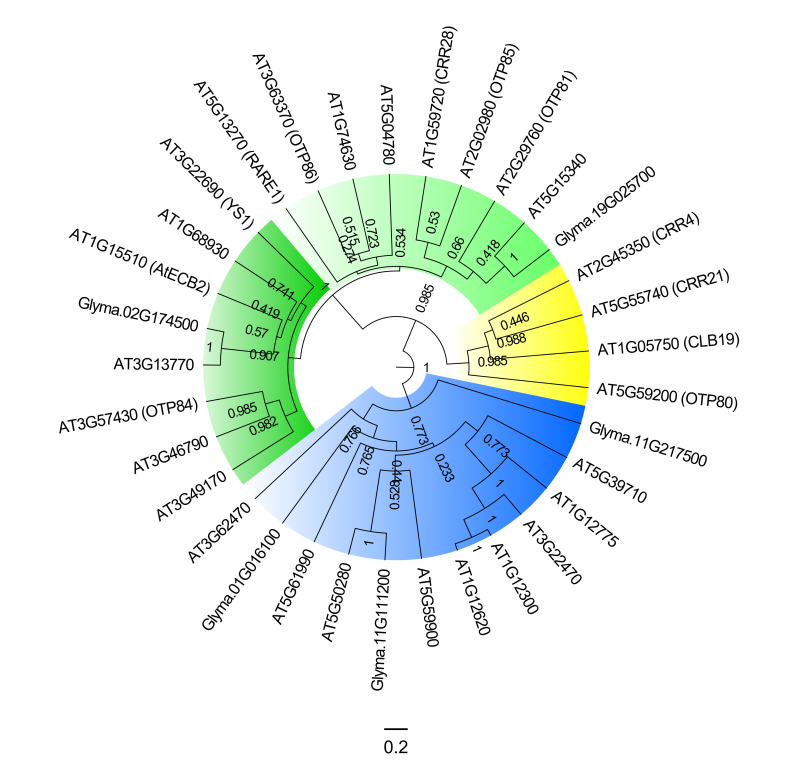
Phylogenetic relationship among PPR protein sequences. The phylogenetic analysis was performed with PPR protein sequences from *A. thaliana* and *G. max*. Posteriori probabilities are labeled above the branches. In blue, PPR P-type proteins; in yellow, PPR E-type proteins; in green, PPR DYW-type proteins.

To identify homologs and understand the evolutionary relationships of the PPRs identified in soybean with those already described in *A. thaliana* as involved in plastid RNA editing, we conducted a phylogenetic analysis using only the sequences encompassing the Pfam domains. The complete dataset consists of 37 sequences, the five soybean PPRs identified by RNA probe pulldown and 32 Arabidopsis PPR proteins (Table S3). The phylogenetic analysis of the PPR amino acid sequences resulted in the formation of well-supported clades separating the different PPR types (Figure 3). Besides that, PPRs from Arabidopsis formed clusters with soybean identified PPR proteins, supported by high posterior probabilities in some cases. Glyma.02G174500 and Glyma.19G025700 grouped respectively to AT3G13770 and AT5G15340 proteins within the DYW-type clade. Glyma.01G016100 grouped to AT5G39710 in a P-type domain clade. The Glyma.11G111200 protein grouped to AT5G50280 in a P-type domain clade. Glyma.11G217500 did not group to any Arabidopsis protein and remained as a basal protein in the P-type clade (Figure 3). Another phylogenetic analysis demonstrated that the Arabidopsis editing *trans*-factors of *atpF-92* (AEF1/MPR25), *ndhB*-1481 (OTP84), and *rps*14-80 (OTP86) do not cluster to soybean PPR proteins found in the pulldown assays. The soybean PPRs isolated from the pulldown continued to cluster to distinct Arabidopsis PPRs (Material S2).

A different approach using BLASTP analysis, against soybean PPRs, was performed to identify the three most similar proteins to Arabidopsis *trans*-factors AEF1/MPR25 (AT3G22150), OTP84 (AT3G57430), and OTP86 (AT3G63370) (Table 2). The RNA binding specificities of the soybean PPRs obtained by BLASTP analysis, as well as of the Arabidopsis *trans*-factors, were evaluated using the aPPRove method (Harrison *et al.*, 2016) and compared to the PPR-probe alignment of Glyma.02G174500, Glyma.01G016100, and Glyma.19G025700. In all PPR-probe alignments evaluated, the soybean PPRs of the probe pulldown assays had the best alignment, with a *p*-value more significant than the Arabidopsis or its most similar soybean PPR (Table 2). All Arabidopsis and their most similar soybean PPRs aligning to RNA *cis*-elements are listed in Material S3.

**Table 2:** PPR-probe alignment comparison among Arabidopsis, soybean PPR most similar to Arabidopsis and soybean PPRs pulled-down by RNA probes.

Protein	Alias	E-value	Editing site	*p*-value
AEF1	AT3G22150.1	-	*atpF*-92	0.003
	Glyma.14G003000.1	0.0	*atpF*-92	0.032
	Glyma.02G309700.1	0.0	*atpF*-92	0.003
	Glyma.06G206900.1	1.3e-127	*atpF*-92	0.122
	Glyma.19G025700.1*	1.59e-62	*atpF*-92	**0.002**
OTP84	AT3G57430.1	-	*ndhB*-1481	0.003
	Glyma.15G156600.1	0.0	*ndhB*-1481	0.009
	Glyma.06G206900.1	0.0	*ndhB*-1481	0.006
	Glyma.15G273200.1	0.0	*ndhB*-1481	0.015
	Glyma.01G016100.1*	6.57e-13	*ndhB*-1481	**0.001**
OTP86	AT3G63370.1	-	*rps14*-80	0.042
	Glyma.02G144100.1	0.0	*rps14*-80	0.052
	Glyma.20G155800.1	0.0	*rps14*-80	0.023
	Glyma.15G273200.1	1.8e-158	*rps14*-80	0.020
	Glyma.02G174500.1*	5.37e-88	*rps14*-80	**0.019**

*: soybean loci isolated using biotinylated RNA probe

### Gene expression analysis of identified PPR genes

**Figure 4:**
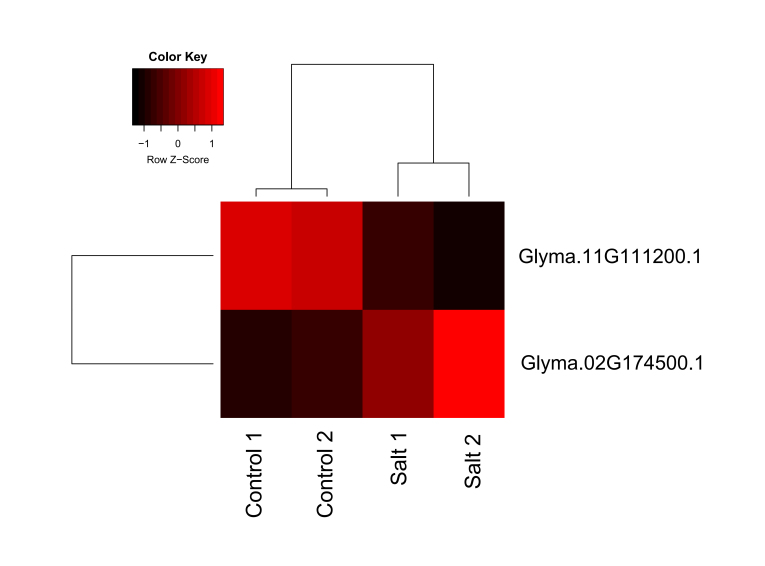
Heatmap showing the relative expression of differentially expressed transcripts of pentatricopeptide proteins pulled-down of *Glycine max* under salt stress. Colors indicate relative expression (red = high, black = low expression). Only transcripts whose adjusted *p*-values did not exceed 0.05 are shown.

A differential gene expression analysis was conducted to evaluate the expression of individual PPRs under salt stress. The five identified PPR genes were evaluated in comparison to another seven reference genes: five eukaryotic elongation factor 1-beta genes (Glyma.02G276600, Glyma.04G195100, Glyma.06G170900, Glyma.13G073200, and Glyma.14G039100) and two F-box genes (Glyma.11G126500.1 and Glyma.12G051100). These genes were already described as reference genes for normalization in soybean under salt stress (Le *et al.*, 2012). Only two genes, Glyma.02G174500 and Glyma.11G111200, both identified in *rps14*-80 probe pulldown, demonstrated differential expression between control and salt treatment libraries Figure 4). Glyma.02G174500 had a 1.09-fold change increase (*p*-value 0.0117), while Glyma.11G111200 had a decrease of -0.65-fold change (*p*-value 0.0004) (Figure S2).

## Discussion

In this work, *cis*-elements and *trans*-factors from three soybean plastid RNA editing sites were analyzed. Each evaluated *cis*-element of an editing site has a conservation pattern that may lead to an alteration in site-recognition of homologous proteins among species. In tobacco plastids, RNA editing sites with similar *cis*-elements are recognized by an identical site-recognition protein (Kobayashi *et al.*, 2007). Along this same line, *in vitro* RNA editing demonstrated that deletions, insertions, and mutations in *cis*-elements could lead to changes in a protein that recognize an editing site between plant species without loss of RNA editing (Neuwirt *et al.*, 2005).

To date, studies that identified RNA editing *trans*- factors and their interactions are based on co-immunoprecipitation and mutant genetic screening, and the model species have been restricted to Arabidopsis, maize, rice, and *Physcomitrella patens* (Sun *et al.*, 2013; Ichinose *et al.*, 2014; Tan *et al.*, 2014). In this study we used an alternative method in the protein isolation step for mass spectrometry assays that allowed us to identify PPR proteins in each probe pulldown. Recently, a study redefined the structural motifs of PPR domains (Cheng *et al.*, 2016). According to this definition and based on our phylogenetic analysis, Glyma.01G016100, Glyma.11G217500, and Glyma.11G111200 belong to the P subfamily, while Glyma.02G174500 and Glyma.19G025700 to DYW subgroup of PLS subfamily. P-type PPR proteins are involved in two main functions: stabilization and processing of specific RNA termini and control of the translation of specific mRNAs (Barkan and Small, 2014). The DYW-type PPR proteins are involved in editing their related editing sites, and in some cases, the DYW domain may participate in the editing of additional sites (Hayes *et al.*, 2015). The distribution of PPR among probe pulldown profile suggests that multiple trans-factors are necessary for editing.

In Arabidopsis, the three editing sites have only one *trans*-factor to RNA editing: AEF1/MRF25 to *atpF*-92 (Yap *et al.*, 2015), OTP84 to *ndhB*-1481, and OTP86 to *rps14*-80 (Hammani *et al.*, 2009). In soybean *atpF-92* and *rps14*-80, a P-type and a DYW-type can interact to promote editing. Some studies have demonstrated the requirement of two PPR proteins for RNA editing in plastids and mitochondria (Guillaumot *et al.*, 2017). The Glyma.11G111200 protein was identified in two pulldown profiles, *ndhB*-1481 and *rps14*-80. OTP82 and CRR22 have been reported to act as site-specificity factors at multiple RNA editing sites with unrelated *cis*-acting elements in plastids (Okuda and Shikanai, 2012). The same can occur with Glyma.11G111200. *In vitro* experiments have demonstrated a cross-competition in plastid RNA editing, suggesting a sharing of *trans*-factors between different editing sites (Heller *et al.*, 2008), and multiple PPR proteins could interact with a unique *cis*-element of an RNA editing site (Andrés-Colás *et al.*, 2017). Sharing of *trans*-factors can confer an advantage by being able to recognize more editing sites with a lower number of required proteins. Besides that, a unique PPR can be a dual target to plastids and mitochondria, acting in different *cis*-elements of different organelles (Ichinose and Sugita, 2016).

An inference of PPR proteins *trans*-factors using phylogenetic analysis can be difficult due to massive gene duplication and evolution of the PPR family in land plants (Hayes and Mulligan, 2011; Cheng *et al.*, 2016). This massive duplication enables the evolution of plant RNA editing *trans*-factors despite changes in the *cis*-element sequence or the loss of editing sites (Hein and Knoop, 2018). Hence, amino acids necessary for the recognition of the *cis*-elements can change over evolutionary time, being able to generate new sites and losing the recognition of already established *cis*-elements. Thus, due to this not-so-simple relationship, methods to identify homologous proteins cannot be used effectively in some cases. The comparison of binding events between Arabidopsis and soybean PPR proteins demonstrates that, despite the similarity, minimal differences among proteins may affect their *cis*-element binding capacity.

In a previous study, we demonstrated some plastid RNA editing enhancement in soybean leaves under salt stress (Rodrigues *et al.*, 2017b). One of them was the *rps14*-80 editing site. Here, we evaluated the expression pattern of PPR transcripts under salt stress. Interestingly, Glyma.02G174500, a DYW-type protein identified in the *rps14*-80 pulldown, has an increase of about one-fold, corresponding to a double increase in its gene expression. Thus, it is plausible to propose that the increase in the editing rate of *rps14*-80 editing site and the increase in Glyma.02G174500 gene expression are related, as it corresponds to its cognate *trans*-factor. The nucleotide alignment with the aPPRove method supports the proposition of the *trans*-factor function of this DYW-type protein in the *rps14*-80 editing site.

**Figure 5:**
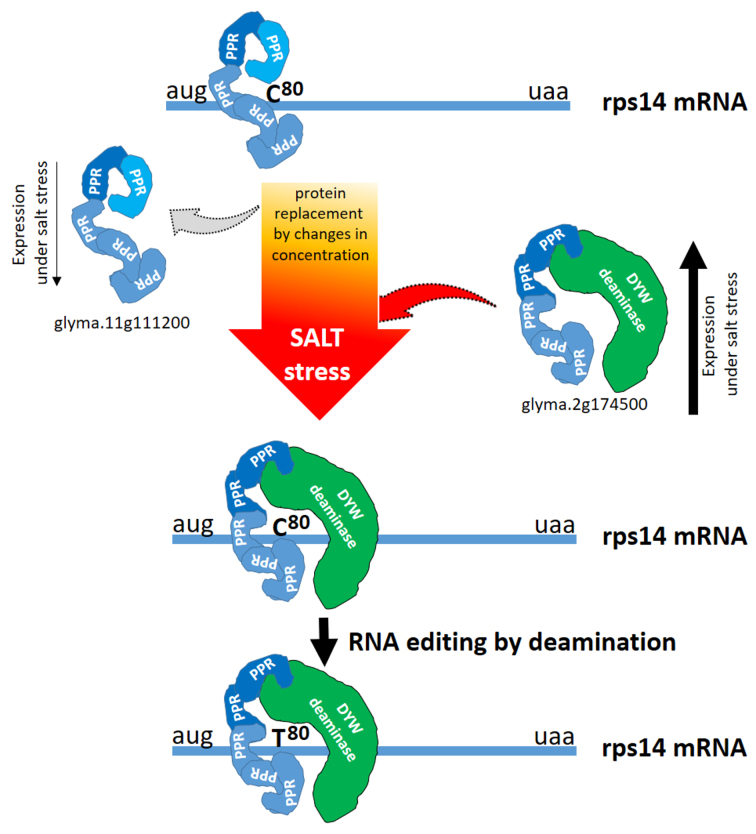
Hypothetical model for the interaction among two soybean PPRs and the *rps14 cis*-element at the editing position C^80^. Glyma.11G111200 has five PPR domains and is expressed in leaves under standard conditions. Glyma.02G174500 is induced under salt stress and contains five PPR plus a deaminase domain with a DYW motif. The model suggests a possible replacement between the two proteins at the RNA *cis*-element, triggered by an alteration in their relative expression levels and an increased C^80^ to T^80^ editing.

A model in which two distinct soybean PPRs can bind to the same *cis*-element under normal physiological and stressed conditions is presented (Figure 5). Under salt stress, the increase in Glyma.02G174500 expression and the decrease in Glyma.11G111200 can lead to a change in protein concentrations and the binding equilibrium at the *rps14*-80 editing site, with a slight increment of the C-to-U editing rate (Figure 5).

The study of the different classes of PPR proteins harboring a diversity of PPR and catalytic domains and their interaction with RNA cis-elements, remains a topic that requires much more investigation, particularly in non-model organisms others than Arabidopsis and rice. As demonstrated by our analysis, it is not easy to identify the homologous sequences of Arabidopsis PPRs in other plant species, and much less so to obtain a good prediction of the *cis*-elements that will be recognized by them.

## Supplementary material

The following online material is available for this article:

Figure S1 Alignment of analyzed *cis*-elements.

Figure S2 Mean difference (M) vs. average in salt-treated versus control soybean leaves.

Table S1 List of species selected to perform the *cis*-element analysis.

Table S2 List of proteins identified by MS/MS approach and the respective probe.

Table S3 List of PPR protein sequences used in phylogenetic analysis.

Material S1 Individual PPR-probe alignments of each RNA probe and their corresponding p-values.

Material S2 Phylogenetic tree of the PPR protein.

Material S3 Arabidopsis and soybean PPR-probe alignments and their corresponding p-values.

## References

[B1] Andrés-Colás N, Zhu Q, Takenaka M, De Rybel B, Weijers D, Van Der Straeten D (2017). Multiple PPR protein interactions are involved in the RNA editing
system in Arabidopsis mitochondria and plastids. Proc Natl Acad Sci U S A.

[B2] Barkan A, Small I (2014). Pentatricopeptide repeat proteins in plants. Annu Rev Plant Biol.

[B3] Bock R, Hermann M, Kössel H (1996). In vivo dissection of cis-acting determinants for plastid RNA
editing. EMBO J.

[B4] Chateigner-Boutin AL, Small I (2010). Plant RNA editing. RNA Biol.

[B5] Cheng S, Gutmann B, Zhong X, Ye Y, Fisher MF, Bai F, Castleden I, Song Y, Song B, Huang J (2016). Redefining the structural motifs that determine RNA binding and
RNA editing by pentatricopeptide repeat proteins in land
plants. Plant J.

[B6] Crooks GE (2004). WebLogo: A sequence logo generator. Genome Res.

[B7] Darriba D, Taboada GL, Doallo R, Posada D (2011). ProtTest- HPC: Fast selection of best-fit models of protein
evolution. Bioinformatics.

[B8] Drummond AJ, Rambaut A (2007). BEAST: Bayesian evolutionary analysis by sampling
trees. BMC Evol Biol.

[B9] Edgar RC (2004). MUSCLE: Multiple sequence alignment with high accuracy and high
throughput. Nucleic Acids Res.

[B10] Finn RD, Coggill P, Eberhardt RY, Eddy SR, Mistry J, Mitchell AL, Potter SC, Punta M, Qureshi M, Sangrador-Vegas A (2016). The Pfam protein families database: Towards a more sustainable
future. Nucleic Acids Res.

[B11] Fujii S, Small I (2011). The evolution of RNA editing and pentatricopeptide repeat
genes. New Phytol.

[B12] Guillaumot D, Lopez-Obando M, Baudry K, Avon A, Rigaill G, Falcon de, Broche B, Takenaka M, Berthomé R, De Jaeger G (2017). Two interacting PPR proteins are major Arabidopsis editing
factors in plastid and mitochondria. Proc Natl Acad SciUSA.

[B13] Hammani K, Okuda K, Tanz SK, Chateigner-Boutin AL, Shikanai T, Small I (2009). A study of new Arabidopsis chloroplast RNA editing mutants
reveals general features of editing factors and their target
sites. Plant Cell.

[B14] Hayes ML, Mulligan RM (2011). Pentatricopeptide repeat proteins constrain genome evolution in
chloroplasts. Mol Biol Evol.

[B15] Hayes ML, Dang KN, Diaz MF, Mulligan RM (2015). A conserved glutamate residue in the C-terminal deaminase domain
of pentatricopeptide repeat proteins is required for RNA editing
activity. J Biol Chem.

[B16] Harrison T, Ruiz J, Sloan DB, Ben-Hur A, Boucher C (2016). aPPRove: An HMM-based method for accurate prediction of
RNA-pentatricopeptide repeat protein binding events. PLoS One.

[B17] Heller WP, Hayes ML, Hanson MR (2008). Cross-competition in editing of chloroplast RNA transcripts in
vitro implicates sharing of trans-factors between different C
targets. J Biol Chem.

[B18] Ichinose M, Sugita M (2016). RNA editing and its molecular mechanism in plant
organelles. Genes (Basel).

[B19] Hein A, Knoop V (2018). Expected and unexpected evolution of plant RNA editing factors
CLB19, CRR28 and RARE1: Retention of CLB19 despite a phylogenetically deep
loss of its two known editing targets in Poaceae. BMC Evol Biol.

[B20] Ichinose M, Uchida M, Sugita M (2014). Identification of a pentatricopeptide repeat RNA editing factor
in Physcomitrella patens chloroplasts. FEBS Lett.

[B21] Kobayashi K, Kawabata M, Hisano K, Kazama T, Matsuoka K, Sugita M, Nakamura T (2012). Identification and characterization of the RNA binding surface of
the pentatrico-peptide repeat protein. Nucleic Acids Res.

[B22] Kobayashi Y, Matsuo M, Sakamoto K, Wakasugi T, Yamada K, Obokata J (2007). Two RNA editing sites with cis-acting elements of moderate
sequence identity are recognized by an identical site-recognition protein in
tobacco chloroplasts. Nucleic Acids Res.

[B23] Langmead B, Trapnell C, Pop M, Salzberg SL (2009). Ultrafast and memory-efficient alignment of short DNA sequences
to the human genome. Genome Biol.

[B24] Le DT, Aldrich DL, Valliyodan B, Watanabe Y, van Ha C, Nishiyama R, Guttikonda SK, Quach TN, Gutierrez-Gonzalez JJ, Tran LSP (2012). Evaluation of candidate reference genes for normalization of
quantitative RT-PCR in soybean tissues under various abiotic stress
conditions. PLoS One.

[B25] Love MI, Huber W, Anders S (2014). Moderated estimation of fold change and dispersion for RNA-seq
data with DESeq2. Genome Biol.

[B26] Miyata Y, Sugita M (2004). Tissue- and stage-specific RNA editing of rps14 transcripts in
moss (Physcomitrella patens) chloroplasts. J Plant Physiol.

[B27] Neuwirt J, Takenaka M, van der Merwe JA, Brennicke A (2005). An in vitro RNA editing system from cauliflower mitochondria:
Editing site recognition parameters can vary in different plant
species. RNA.

[B28] Okuda K, Shikanai T (2012). A pentatricopeptide repeat protein acts as a site-specificity
factor at multiple RNA editing sites with unrelated cis-acting elements in
plastids. Nucleic Acids Res.

[B29] Rodrigues NF, Christoff AP, Fonseca GC, Kulcheski FR, Margis R (2017). Front Plant Sci.

[B30] Rodrigues NF, Fonseca GC, Kulcheski FR, Margis R (2017). Genet Mol Biol.

[B31] Schallenberg-Rüdinger M, Knoop V (2016). Coevolution of organelle RNA editing and nuclear specificity
factors in early land plants. Adv Bot Res.

[B32] Small ID, Peeters N (2000). The PPR motif - a TPR-related motif prevalent in plant organellar
proteins. Trends Biochem Sci.

[B33] Sun T, Germain A, Giloteaux L, Hammani K, Barkan A, Hanson MR, Bentolila S (2013). An RNA recognition motif-containing protein is required for
plastid RNA editing in Arabidopsis and maize. Proc Natl Acad Sci U S A.

[B34] Takenaka M, Zehrmann A, Verbitskiy D, Härtel B, Brennicke A (2013). RNA editing in plants and its evolution. Annu Rev Genet.

[B35] Tamura K, Stecher G, Peterson D, Filipski A, Kumar S (2013). MEGA6: Molecular evolutionary genetics analysis version
6.0. Mol Biol Evol.

[B36] Tan J, Tan Z, Wu F, Sheng P, Heng Y, Wang X, Ren Y, Wang J, Guo X, Zhang X (2014). A novel chloroplast-localized pentatricopeptide repeat protein
involved in splicing affects chloroplast development and abiotic stress
response in rice. Mol Plant.

[B37] Timmis JN, Ayliffe MA, Huang CY, Martin W (2004). Endosymbiotic gene transfer: organelle genomes forge eukaryotic
chromosomes. Nat Rev Genet.

[B38] Tseng CC, Lee CJ, Chung YT, Sung TY, Hsieh MH (2013). Differential regulation of Arabidopsis plastid gene expression
and RNA editing in non-photosynthetic tissues. Plant Mol Biol.

[B39] Vieira LDN, Faoro H, Fraga HP, Rogalski M, De Souza EM, De Oliveira Pedrosa F, Nodari RO, Guerra MP (2014). An improved protocol for intact chloroplasts and cpDNA isolation
in conifers. PLoS One.

[B40] Vu LT, Tsukahara T (2017). C-to-U editing and site-directed RNA editing for the correction
of genetic mutations. Biosci Trends.

[B41] Yap A, Kindgren P, Colas des, Kazama T, Tanz SK, Toriyama K, Small I (2015). AEF1/MPR25 is implicated in RNA editing of plastid atpF and
mitochondrial nad5, and also promotes atpF splicing in Arabidopsis and
rice. Plant J.

