# Supplementary material for: Identification of soybean trans-factors associated with plastid RNA editing sites
Source: Genet Mol Biol. 2020 May 11;43(1 Suppl 2):e20190067. doi: 10.1590/1678-4685-GMB-2019-0067 (PMC7231544; doi:10.1590/1678-4685-GMB-2019-0067)
Supplement: Figure S1 [file 1415-4757-gmb-43-1-s2-e20190067-suppl1.pdf]

Genetics and Molecular Biology 43, 1(suppl 2) (2020)

Supplementary Material to "Identification of soybean *trans*-factors associated with plastid RNA editing sites"

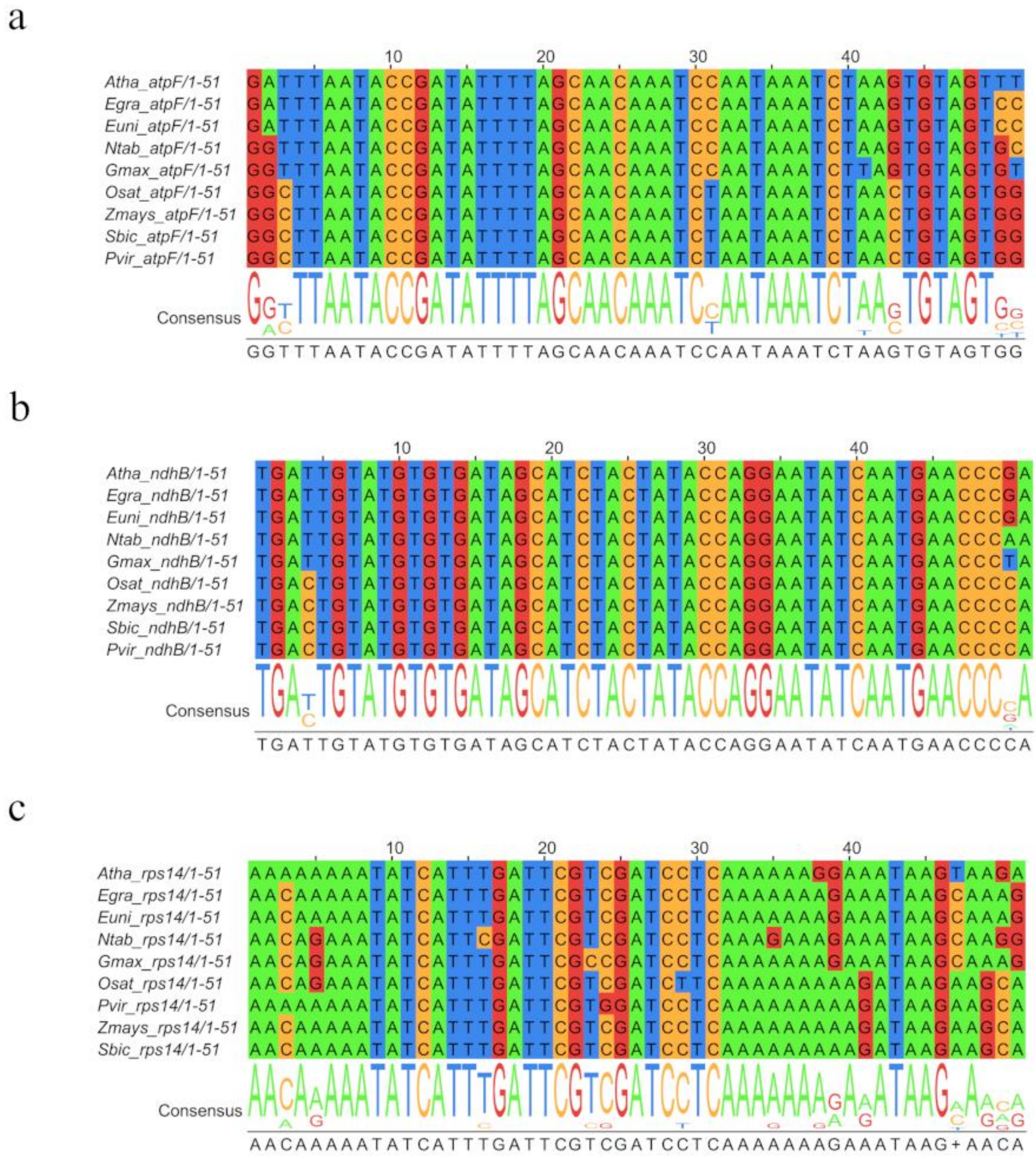

Figure S1 - Alignment of analyzed *cis*-elements. Sequence alignment of the region surrounding the (a) *atpF-92*, (b) *ndhB*-1481, and (c) *rps14*-80 editing sites. The alignment includes the sequence from -30 to +20 around the edited C (position 31) of *A. thaliana* (*Atha*), *E. uniflora* (*Euni*), *G. max* (*Gmax*), *N. tabacum* (*Ntab*), *O. sativa* (*Osat*), *P. virgatum* (*Pvir*), *S. bicolor* (*Sbic*), and *Z. mays* (*Zmay*). Above each alignment, a consensus logo is shown.
