# Supplementary material for: Identification of soybean trans-factors associated with plastid RNA editing sites
Source: Genet Mol Biol. 2020 May 11;43(1 Suppl 2):e20190067. doi: 10.1590/1678-4685-GMB-2019-0067 (PMC7231544; doi:10.1590/1678-4685-GMB-2019-0067)
Supplement: Figure S2 [file 1415-4757-gmb-43-1-s2-e20190067-suppl2.pdf]

Supplementary Material to "Identification of soybean *trans*-factors associated with plastid RNA editing sites"

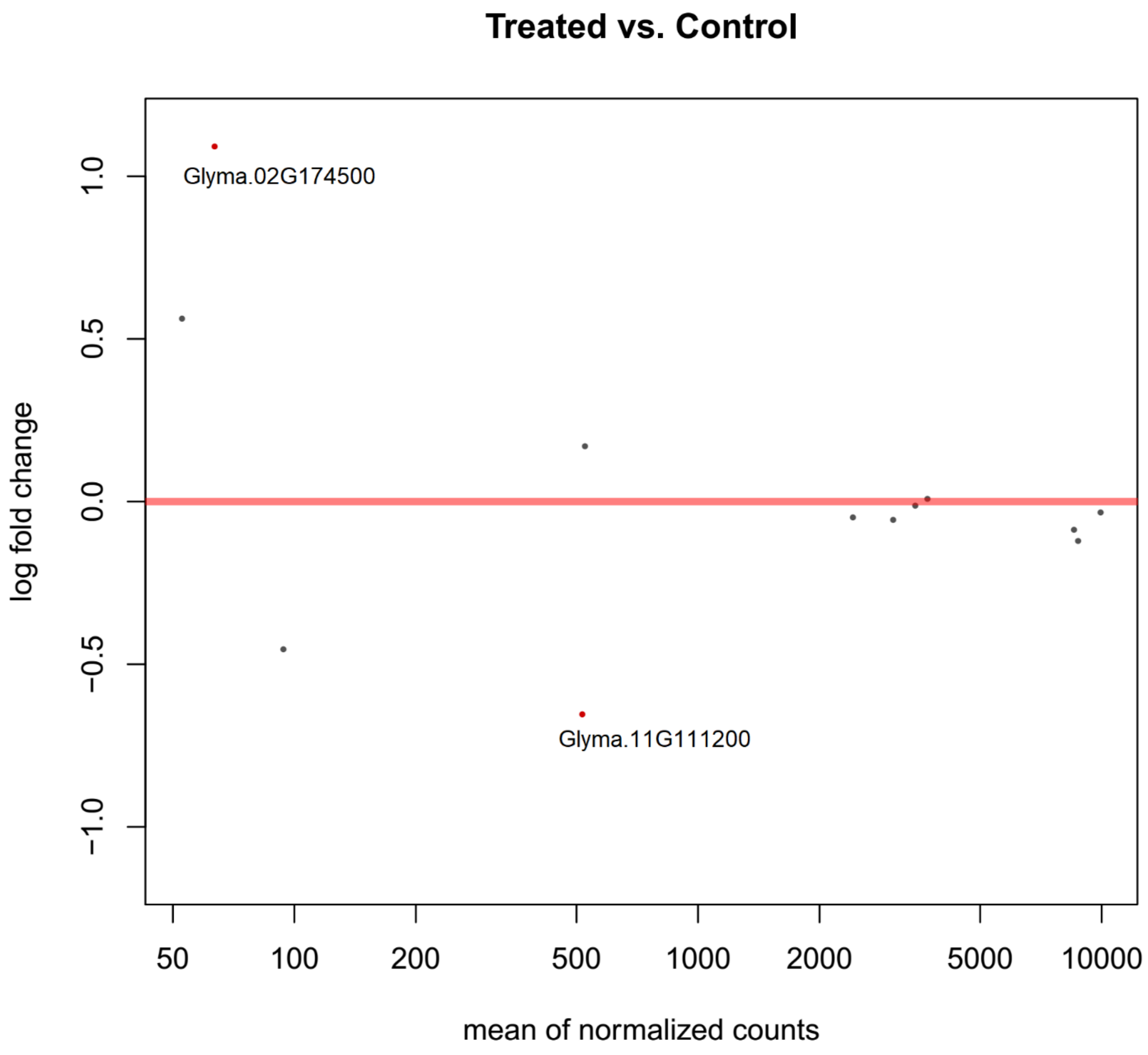

Figure S2 - Mean difference (M) vs. average expression (A) plot of differential gene expression in salt-treated versus control soybean leaves. The red dots indicate differentially expressed genes.
