# Supplementary material for: Identification of soybean trans-factors associated with plastid RNA editing sites
Source: Genet Mol Biol. 2020 May 11;43(1 Suppl 2):e20190067. doi: 10.1590/1678-4685-GMB-2019-0067 (PMC7231544; doi:10.1590/1678-4685-GMB-2019-0067)
Supplement: Table S1 [file 1415-4757-gmb-43-1-s2-e20190067-suppl3.pdf]

Supplementary Material to "Identification of soybean *trans*-factors associated with plastid RNA editing sites"

Table S1 - List of species selected to perform the *cis*-element analysis

Species	Accession
Arabidopsis thaliana	NC_000932.1
Eucalyptus grandis	NC_014570.1
Eugenia uniflora	NC_027744.1
Nicotiana tabacum	NC_001879.2
Oryza sativa	NC_001320.1
Panicum virgatum	NC_015990.1
Sorghum bicolor	NC_008602.1
Zea mays	NC_001666.2
