# Supplementary material for: Identification of soybean trans-factors associated with plastid RNA editing sites
Source: Genet Mol Biol. 2020 May 11;43(1 Suppl 2):e20190067. doi: 10.1590/1678-4685-GMB-2019-0067 (PMC7231544; doi:10.1590/1678-4685-GMB-2019-0067)
Supplement: Table S2 [file 1415-4757-gmb-43-1-s2-e20190067-suppl4.pdf]

Supplementary Material to "Identification of soybean *trans*-factors associated with plastid RNA editing sites"

Table S2 - List of proteins identified by MS/MS approach and respective probe

Confidence	Sequence	Modifications	Protein Groups	Proteins	PSMs	Master Protein Accessions	Description	Missed Cleavages	Theo. MH+ [Da]	atpF_01	atpF_02	ndhB_01	ndhB_02	rps14_01	rps14_02	Control_01	Control_02
Medium	FVTATQTSVANMMSRSGNALR		1	3	1	Glyma.01G014700.1.p	TRANSCRIPTION FACTOR JUMONJI (JMJ) FAMILY PROTEIN / ZINC FINGER (C5HC2 TYPE) FAMILY	1	2242.106695	N	N	N	N	M	N	N	N
Medium	HGYFDQAHKLYDVMK		1	1	1	Glyma.01G016100.1.p	K17964 - leucine-rich PPR motif-containing protein, mitochondrial (LRPPRC)	1	1851.884665	N	N	M	N	N	N	N	N
Medium	MGFSEKPQVEESDSTR	1xOxidation [M1]	1	2	3	Glyma.01G026600.1.p	no GO	0	1842.817433	N	N	N	M	M	M	N	N
Medium	VEVMVTQNNESGKFLLWDR		1	3	1	Glyma.01G065300.1.p	PTHR23273//PTHR23273:SF8 - REPLICATION FACTOR A 1, RFA1 // SUBFAMILY NOT NAMED	1	2265.133228	N	N	N	M	N	N	N	N
Medium	DPCLPGVKDAIQLCQK	2xCarbamidomethyl [C3; C14]	1	1	1	Glyma.01G094400.1.p	Calcium-transporting ATPase / Calcium-translocating P-type ATPase	1	1841.924816	N	N	N	N	N	N	N	M
Medium	QATMYILSLTKALAYCHEK	1xCarbamidomethyl [C16]	1	1	1	Glyma.01G145100.1.p	KOG0662 - Cyclin-dependent kinase CDK5	1	2241.140622	N	N	N	N	N	N	N	M
Medium	YPNLPQTFLDATK		1	3	2	Glyma.01G152100.3.p	PTHR33101:SF2 - ROP GUANINE NUCLEOTIDE EXCHANGE FACTOR 14	0	1507.77912	N	N	N	M	N	N	N	M
High	FENEVSSASTASTASAPR		1	1	3	Glyma.01G172500.1.p	K10756 - replication factor C subunit 3/5 (RFC3_5)	0	1811.840611	N	N	N	M	N	H	N	M
Medium	HVSSTTIQRFPSMGNISNQVAADGNNSHLPHSR		1	1	1	Glyma.01G209200.1.p	PTHR13402 - RGPR-RELATED	1	3559.720733	N	M	N	N	N	N	N	N
Medium	DHIKGGSDHASDNSNEK		1	1	11	Glyma.01G231400.1.p	K14826 - FK506-binding nuclear protein [EC:5.2.1.8] (FPR3_4)	1	1810.795058	M	M	M	N	N	N	N	N
Medium	IMKSYVTFKDNIPSGGVSSQLSHEDEMLSGATIFNMK	2xOxidation [M27; M36]	1	1	1	Glyma.01G238600.1.p	PTHR19375:SF101 - HEAT SHOCK 70 KDA PROTEIN 8	2	4093.955529	N	N	M	N	N	N	N	N
Medium	VKDMMDLESVHMEYLADSLCIWIINFSLHPSSNPRCIK	2xCarbamidomethyl [C20; C36]; 2xOxidation [M5; M12]	1	1	1	Glyma.01G244400.3.p	K16573 - gamma-tubulin complex component 6 (TUBGCP6, GCP6)	2	4581.17433	N	M	N	N	N	N	N	N
Medium	WNCAVCNQKQSVCR	3xCarbamidomethyl [C3; C6; C13]	1	4	1	Glyma.02G077500.1.p	PTHR15863:SF2 - UPF0544 PROTEIN C5ORF45	1	1809.794153	N	N	N	N	N	N	N	M

Confidence	Sequence	Modifications	Protein Groups	Proteins	PSMs	Master Protein Accessions	Description	Missed Cleavages	Theo. MH+ [Da]	atpF_01	atpF_02	ndhB_01	ndhB_02	rps14_01	rps14_02	Control_01	Control_02
Medium	AIDFTPESRIGQSSAICLELPNGRQLPNFR	1xCarbamidomethyl [C17]	1	1	1	Glyma.02G086100.1.p	PTHR23079:SF9 - RNA-DEPENDENT RNA POLYMERASE 1	2	3386.727382	N	N	N	N	M	N	N	N
High	TDNEIKNYWNTNVKK		1	3	1	Glyma.02G110100.1.p	PTHR10641//PTHR10641:SF626 - MYB-LIKE DNA-BINDING PROTEIN MYB // SUBFAMILY NOT NAMED	2	1866.934452	N	N	N	N	N	N	N	H
High	LSDLSAAADVNLK		1	1	17	Glyma.02G111800.2.p	PTHR22883//PTHR22883:SF96 - ZINC FINGER DHHC DOMAIN CONTAINING PROTEIN // SUBFAMILY NOT NAMED	0	1316.705621	N	N	N	H	M	N	M	H
High	SSSVSGGQGLIFGNKNMNK	1xOxidation [M17]	1	1	14	Glyma.02G119000.1.p	PTHR18934:SF143 - DEAD/DEAH BOX HELICASE	1	1940.94945	N	M	H	H	M	M	N	H
Medium	FRPERFVEEDVSIMGSDLR		1	2	2	Glyma.02G119600.1.p	PTHR24298:SF112 - CYTOCHROME P450 78A5-RELATED	1	2282.123391	N	N	N	M	N	N	N	M
Medium	NTNFKNQNVENIQIYHYK		1	1	1	Glyma.02G136100.1.p	PTHR10209//PTHR10209:SF98 - OXIDOREDUCTASE, 2OG-FE II OXYGENASE FAMILY PROTEIN // SUBFAMILY NOT NAMED	1	2267.120355	N	N	N	N	N	N	N	M
Medium	SLQLQVQMMSMGCGIIPMIFPGIQQYMPPMGMAIGMGMGMGMEMR	1xCarbamidomethyl [C13]	1	1	1	Glyma.02G160200.1.p	PTHR12565:SF86 - TRANSCRIPTION FACTOR PIF4-RELATED	0	4967.243435	N	N	N	N	N	N	N	M
Medium	KETTSSSGSPTQTGSSTQK		1	1	1	Glyma.02G164000.1.p	no GO	1	1898.893768	M	N	N	N	N	N	N	N
High	LDEMLQNQGVKVNK		1	1	3	Glyma.02G164900.1.p	PTHR23359//PTHR23359:SF99 - NUCLEOTIDE KINASE // SUBFAMILY NOT NAMED	1	1615.847217	M	N	N	N	N	N	N	H
Medium	AIDISGVSMLVIDGLNTICSAGHADTVK	1xCarbamidomethyl [C19]; 1xOxidation [M9]	1	1	1	Glyma.02G165400.1.p	PTHR24031:SF283 - DEAD/DEAH BOX HELICASE-LIKE PROTEIN	0	2873.43832	N	N	N	N	N	N	N	M
Medium	HGEGREVLELFNLMIDENK		0	1	1	Glyma.02G174500	PPR repeat (PPR) // PPR repeat family (PPR_2) // DYW family of nucleic acid deaminases (DYW_deaminase)	1	4078.949807	N	N	N	N	N	M	N	N
Medium	ENNQSCLLHNVEQKHINLCDAADDFKPSWNIQLK	2xCarbamidomethyl [C6; C19]	1	1	1	Glyma.02G189600.1.p	PTHR10108:SF37 - METHYLTRANSFERASE PMT6-RELATED	0	1750.856779	M	N	N	N	N	N	N	N
Medium	FLGDDMVLLPGLSDDK	1xOxidation [M6]	1	1	1	Glyma.02G200800.1.p	PTHR23147:SF4 - SERINE/ARGININE-RICH SPLICING FACTOR 2	1	2335.116057	N	N	N	N	N	N	N	M

Confidence	Sequence	Modifications	Protein Groups	Proteins	PSMs	Master Protein Accessions	Description	Missed Cleavages	Theo. MH+ [Da]	atpF_01	atpF_02	ndhB_01	ndhB_02	rps14_01	rps14_02	Control_01	Control_02
Medium	ATNSSEGSPGSVKANGFSSTQPPK		1	2	1	Glyma.02G201300.4.p	PTHR23083:SF397 - UDP-N-ACETYLGLUCOSAMINE--PEPTIDE N-ACETYLGLUCOSAMINYLTRANSFERASE SPINDLY-RELATED	0	1546.811149	N	N	N	N	N	N	N	M
Medium	LPFGLCGCQEENSKGR	2xCarbamidomethyl [C6; C8]	0	4	1	Glyma.02G229900	MITOGEN-ACTIVATED PROTEIN KINASE KINASE KINASE MOM-4-RELATED	0	4124.072748	N	N	N	N	N	N	N	M
Medium	VFGGDNIEGTLISPK		1	2	2	Glyma.02G257800.1.p	K08518 - syntaxin-binding protein 5 (STXBP5)	1	1614.822107	H	N	N	M	N	N	N	H
Medium	EFTMVSLLSACAHLGALQHFELNVIVLTAIIDMYCK	2xCarbamidomethyl [C11; C35]; 1xOxidation [M33]	1	1	1	Glyma.02G285400.1.p	PF01535//PF12854 - PPR repeat (PPR) // PPR repeat (PPR_1)	2	1546.807995	N	N	N	N	N	N	N	M
High	EQDDLLSDLKDVPK		1	2	13	Glyma.02G294200.1.p	PTHR11216//PTHR11216:SF73 - EH DOMAIN // SUBFAMILY NOT NAMED	1	2282.103427	N	M	N	N	N	N	N	N
Medium	EMPAMIGKAKAQQK	1xOxidation [M2]	1	2	1	Glyma.02G294200.1.p	PTHR11216//PTHR11216:SF73 - EH DOMAIN // SUBFAMILY NOT NAMED	1	3052.671748	N	N	N	N	N	N	N	M
Medium	DAVYEADDLLDEVSTKAATQK		1	1	1	Glyma.03G039200.1.p	PTHR23155//PTHR23155:SF562 - LEUCINE-RICH REPEAT-CONTAINING PROTEIN // SUBFAMILY NOT NAMED	2	4013.916047	M	N	N	N	N	N	N	N
Medium	LQNLQLLNVSGCEK	1xCarbamidomethyl [C12]	0	1	1	Glyma.03G048300	LEUCINE-RICH REPEAT-CONTAINING PROTEIN // SUBFAMILY NOT NAMED	1	1002.521449	N	N	N	N	N	N	M	N
Medium	LDKLTYLNLSYAGFVGQIPIEISQLTR		1	2	1	Glyma.03G052400.1.p	PTHR27004:SF37 - DISEASE RESISTANCE FAMILY PROTEIN/LRR FAMILY PROTEIN-RELATED	0	1366.634113	N	H	N	N	N	N	N	N
Medium	MARAFGGDQIEGNTNRVVGTYGYMAPEYAVDGLFSIK	1xOxidation [M24]	1	2	1	Glyma.03G055000.3.p	Non-specific serine/threonine protein kinase / Threonine-specific protein kinase	2	2227.085032	N	N	N	M	N	N	N	N
Medium	SLGEDPTRK		1	1	1	Glyma.03G085100.1.p	GTP cyclohydrolase I	0	2764.342428	M	N	N	N	N	N	N	N
High	MEVMQDAGASAIK	1xOxidation [M4]	1	1	1	Glyma.03G116100.1.p	UDP-GLYCOSYLTRANSFERASE 72B2-RELATED	0	1809.824961	N	N	N	M	N	M	N	M
Medium	DALYLIDIGQNDLADSFAK		0	1	1	Glyma.03G229600	GDSL ESTERASE/LIPASE LIP-4	2	2284.1568	M	M	M	M	N	H	N	N
Medium	ISRDVHEARGPDEVNNFGSK		1	2	1	Glyma.03G232200.1.p	PTHR21726:SF29 - F1O19.10 PROTEIN	2	2282.10161	N	N	N	N	N	N	N	M

Confidence	Sequence	Modifications	Protein Groups	Proteins	PSMs	Master Protein Accessions	Description	Missed Cleavages	Theo. MH+ [Da]	atpF_01	atpF_02	ndhB_01	ndhB_02	rps14_01	rps14_02	Control_01	Control_02
Medium	SDINTPGSVTLDPHPLTSEAWEELR		1	2	1	Glyma.04G005700.3.p	Beta-fructofuranosidase / Saccharase	2	1303.715081	N	N	N	N	N	N	N	M
Medium	NTSEDGTSQSLDWITR		1	2	3	Glyma.04G012800.1.p	LEUCINE-RICH REPEAT PROTEIN KINASE-LIKE PROTEIN	2	3321.767263	N	N	M	N	N	N	N	N
High	YKVPENNNDSKTALLPHTDK		1	1	10	Glyma.04G070500.1.p	OXIDOREDUCTASE, 2OG-FE II OXYGENASE FAMILY PROTEIN // SUBFAMILY NOT NAMED	1	2226.137585	N	N	N	N	N	M	N	N
Medium	KASSRETMGVGGGNGPGVQAFMK	1xOxidation [M8]	1	1	1	Glyma.04G094400.1.p	kinesin family member 4/21/27 (KIF4_21_27)	0	1826.837774	N	N	N	N	N	N	N	M
Medium	IKNMDLEIRR	1xOxidation [M4]	1	2	1	Glyma.04G125800.1.p	vacuolar protein sorting-associated protein 26 (VPS26)	1	1865.822869	N	N	N	N	N	N	N	M
Medium	KHIYWILCATHCIDLMLEDIGKLPLIR	2xCarbamidomethyl [C8; C12]	1	1	1	Glyma.04G163700.1.p	Protein of unknown function (DUF 659) (DUF659) // hAT family C-terminal dimerisation region (Dimer_Tnp_hAT)	0	1477.814837	N	N	N	N	N	H	N	N
Medium	FSLMGLITNYFGPPRPGKQS	1xOxidation [M4]	1	1	1	Glyma.04G163800.1.p	no GO	2	1686.947206	N	N	N	N	N	N	N	M
Medium	CDPFSGELFTVQSSPR	1xCarbamidomethyl [C1]	1	1	1	Glyma.04G176400.1.p	Quinoprotein glucose dehydrogenase (PQQ, quinone) / Quinoprotein D-glucose dehydrogenase	0	2012.000483	H	N	N	N	N	N	N	N
Medium	SITCMVNRQEEGDNGR	1xCarbamidomethyl [C4]	1	1	1	Glyma.04G233400.3.p	TRANSDUCIN/WD40 DOMAIN-CONTAINING PROTEIN	0	1122.527322	N	N	N	M	N	N	N	N
High	EVFLVELVDSISK		1	2	1	Glyma.04G240400.1.p	vacuolar protein sorting-associated protein 45 (VPS45)	0	2309.054126	N	N	N	N	N	N	N	M
Medium	FGLKPQGKSAPMARAK		1	2	1	Glyma.04G246200.1.p	AUXILIN/CYCLIN G-ASSOCIATED KINASE-RELATED	1	2227.106345	H	N	N	N	M	N	N	N
High	LYNNVIEETASMLTELR	1xOxidation [M12]	1	1	1	Glyma.05G023500.1.p	CALMODULIN-BINDING PROTEIN-LIKE PROTEIN	1	2211.11143	M	M	N	M	M	H	N	H
Medium	SDSVISGGADSK		1	2	1	Glyma.05G027800.1.p	ribosome biogenesis protein YTM1 (YTM1, WDR12)	1	3323.741819	N	N	M	N	N	N	N	N
Medium	LEEHGGNLNEAVNAHFTEGDR		1	1	1	Glyma.05G049200.1.p	PTHR23322:SF3 - PROTEIN SAY1	1	2227.11823	N	N	N	N	H	N	N	N
High	NWYLSTLTDVEEVKMVQR	1xOxidation [M15]	1	1	3	Glyma.05G056900.1.p	Nitrate-transporting ATPase	1	1841.807796	N	N	N	H	N	M	N	H
High	NWYLSTLTDVEEVKMVQR		1	1	13	Glyma.05G056900.1.p	Nitrate-transporting ATPase	0	2298.089314	M	N	N	N	N	N	N	N
Medium	TSFKAFMASNTIAMVLASTAAFINLFTPLTK	1xOxidation [M]	1	1	1	Glyma.05G124700.1.p	Reverse transcriptase (RNA-dependent DNA polymerase) (RVT_2) // Ankyrin repeats	1	2905.384369	N	N	N	N	M	N	N	M
High	FHKGIPTLPPNFEEDTWAK		1	1	3	Glyma.05G140800.1.p	cullin 4 (CUL4)	2	4696.161734	N	N	M	N	N	N	N	N
High	MELDQLCKNMDTSEK	1xCarbamidomethyl [C7]	1	1	6	Glyma.05G144200.3.p	LUNG CANCER METASTASIS-RELATED LCMR1 PROTEIN	2	4697.35221	M	N	N	N	N	N	N	N

Confidence	Sequence	Modifications	Protein Groups	Proteins	PSMs	Master Protein Accessions	Description	Missed Cleavages	Theo. MH+ [Da]	atpF_01	atpF_02	ndhB_01	ndhB_02	rps14_01	rps14_02	Control_01	Control_02
Medium	VFASEAQIMEANNAMAWGTLK	1xOxidation [M15]	1	1	1	Glyma.05G144900.1.p	YqaJ-like viral recombinase domain (YqaJ)	2	2409.161568	N	N	N	M	N	N	N	N
Medium	MNSVSEIVEEVNALEAQRTELE ENR	1xOxidation [M1]	1	3	2	Glyma.05G169000.1.p	kinetochore protein Nuf2 (NUF2, CDCA1)	2	1491.801312	N	N	N	N	N	N	N	H
Medium	NLQDELDNVVGRDK		0	1	1	Glyma.05G233200	Cytochrome P450 CYP2 subfamily	2	2377.225882	N	N	N	N	N	N	N	M
Medium	VAEIVDKGSTSCGASGALYTLI CCVIGCGCLYSCFYRPKMR	6xCarbamidomethyl [C12; C23; C24; C28; C30; C34]; 1xOxidation [M40]	1	1	1	Glyma.05G236300.1.p	PLAC8 family (PLAC8)	1	2225.141685	N	N	M	N	M	N	N	N
Medium	ITFEDRDYVVQELTPESFDGVDI ALFSAGGSISKHFGPIAVNR		1	1	1	Glyma.05G240700.1.p	aspartate-semialdehyde dehydrogenase (asd)	2	1317.712104	N	M	N	N	N	N	N	M
Medium	TEKFINRGSGEPVHMSGAYNSK		1	1	1	Glyma.05G241600.3.p	Histidine kinase / Protein kinase (histidine) // Protein-serine/threonine phosphatase / Serine/threonine specific protein phosphatase	1	1717.873038	N	N	N	N	N	N	N	M
High	SSSQIAKVEGKETK		1	2	3	Glyma.05G242900.1.p	GENOMIC DNA, CHROMOSOME 3, P1 CLONE: MRC8	0	1827.80338	N	N	M	N	N	N	N	N
Medium	FVSQGRDGTVKVWELGDAGLS R		1	1	1	Glyma.06G026400.1.p	GUANINE NUCLEOTIDE-BINDING PROTEIN SUBUNIT BETA-LIKE PROTEIN 1	2	3249.522276	N	N	N	N	N	N	M	N
Medium	GGCLAAFKVSSTVVVVAEMQR	1xCarbamidomethyl [C3]; 1xOxidation [M19]	1	1	2	Glyma.06G031600.1.p	DISCO-INTERACTING PROTEIN 2 DIP2 -RELATED // SUBFAMILY NOT NAMED	1	2226.078081	N	N	M	N	M	N	N	H
Medium	SGGKTDVNGAGKVK		1	3	2	Glyma.06G062700.1.p	ASPARTYL BETA-HYDROXYLASE N-TERMINAL REGION DOMAIN-CONTAINING PROTEIN-RELATED	0	866.459124	H	N	N	N	N	N	N	N
Medium	VKTILEQGFYFCGR	1xCarbamidomethyl [C12]	1	2	1	Glyma.06G068900.1.p	PROTEIN RRF-3	2	2239.128811	N	M	N	N	N	N	N	H
Medium	DDLGKGAIDKEVVEK		0	4	1	Glyma.06G083100	DIENELACTONE HYDROLASE	1	2120.065208	N	N	N	N	N	N	N	M
Medium	CVQMIQELSCNSGSAK	2xCarbamidomethyl [C1; C10]; 1xOxidation [M4]	1	1	1	Glyma.06G094600.1.p	CYCLIN-D4-1-RELATED	2	2239.14273	N	N	N	N	N	N	N	H
Medium	VEATAENNAASNRSSGDAACIQ RNMELIEK	1xCarbamidomethyl [C20]	1	3	1	Glyma.06G136600.1.p	pseudo-response regulator 5 (PRR5)	1	2968.414547	N	N	N	N	N	N	M	N
High	EIDLYSTNPECVKMLVGNK	1xCarbamidomethyl [C11]; 1xOxidation [M14]	1	1	3	Glyma.06G154000.1.p	Ras-related protein Rab-18 (RAB18)	1	1557.801981	N	N	N	N	N	N	N	M

Confidence	Sequence	Modifications	Protein Groups	Proteins	PSMs	Master Protein Accessions	Description	Missed Cleavages	Theo. MH+ [Da]	atpF_01	atpF_02	ndhB_01	ndhB_02	rps14_01	rps14_02	Control_01	Control_02
High	HGNVVNAR		1	1	1	Glyma.06G175400.1.p	nucleolin (NCL, NSR1)	1	1851.955915	N	N	N	N	N	N	N	M
High	HVPTSPCKVNQDIGSWASKK	1xCarbamidomethyl [C7]	1	1	3	Glyma.06G202900.1.p	no GO	1	1477.826071	N	N	N	M	N	N	N	M
Medium	VMDLSNVEQGKEITNLSSR		1	1	1	Glyma.06G217700.1.p	NUCLEOPORIN-RELATED PROTEIN	0	3176.519817	N	N	N	N	N	N	N	M
High	VKDDGFGWSGTDVLVMAKIGK	1xOxidation [M16]	1	1	1	Glyma.06G221100.1.p	PHLOEM PROTEIN 2-A10-RELATED	1	1986.054232	H	H	N	N	N	N	N	N
Medium	VGENAWSLMHSSIPDKLQSLYNDGYK	1xOxidation [M9]	1	1	1	Glyma.06G229100.1.p	bifunctional polynucleotide phosphatase/kinase [EC:3.1.3.32 2.7.1.78] (PNKP)	1	1496.814778	H	H	N	N	N	H	N	N
Medium	LDIKNYHDALNNK		1	1	1	Glyma.06G253500.1.p	PROTEIN ULTRAPETALA 1-RELATED	1	1361.709326	N	N	N	M	N	N	M	N
Medium	RGLFQSDAALLESSTTR		1	1	2	Glyma.06G302700.1.p	PEROXIDASE 3-RELATED	2	1507.785862	N	N	N	N	N	N	N	M
Medium	YTPEVIKALAASSK		1	3	2	Glyma.06G310500.1.p	DNA replication ATP-dependent helicase Dna2 (DNA2)	1	1395.741295	N	N	N	N	N	N	N	M
Medium	TQTQTPSSLSLCTSKPQEGNMALESQPQK	1xCarbamidomethyl [C12]	1	1	1	Glyma.07G001800.1.p	solute carrier family 25 (mitochondrial phosphate transporter), member 23/24/25/41 (SLC25A23S)	0	2526.163284	N	N	N	N	N	N	N	M
High	LILKDPQDPSTPNAVSYK		1	1	4	Glyma.07G014500.1.p	ACID PHOSPHATASE-RELATED	1	2225.156072	N	N	N	N	N	H	N	H
High	TFKLPNPLYYIQ		1	1	5	Glyma.07G014500.1.p	ACID PHOSPHATASE-RELATED	0	1842.81156	N	N	N	M	N	N	N	N
Medium	VKEMAQGATEAVK		1	1	2	Glyma.07G032400.1.p	LATE EMBRYOGENESIS ABUNDANT PLANTS LEA-RELATED	2	2241.13573	N	N	N	N	N	N	N	M
Medium	MLEEKGLGKMLDK	1xOxidation [M1]	1	1	1	Glyma.07G077200.1.p	FAS-associated factor 2 (FAF2, UBXD8)	1	1491.790948	H	N	N	H	N	N	N	M
Medium	QELKPMQEHKK		1	1	1	Glyma.07G111300.1.p	Plant protein of unknown function (DUF247)	2	2282.115998	N	N	N	N	N	N	N	M
Medium	MNIMSGCSESSTKPNTIINNSPK	1xCarbamidomethyl [C7]; 1xOxidation [M]	1	1	2	Glyma.07G131900.1.p	no GO	1	1393.693099	N	N	N	M	N	N	N	N
High	SKFVQASGGEGVQATLNQLYK		1	1	8	Glyma.07G133300.1.p	HAT FAMILY DIMERIZATION DOMAIN-CONTAINING PROTEIN	0	2050.042622	N	N	N	N	M	N	N	N
Medium	CWVGSFSSEHASVFDK	1xCarbamidomethyl [C1]	1	1	1	Glyma.07G134000.1.p	PTHR37723:SF1 - FHL-3	1	1489.779789	N	N	N	M	N	N	N	N
Medium	VKVNDYDEITSVVGSSASKSR		1	1	1	Glyma.07G141900.1.p	PF12043 - Domain of unknown function (DUF3527) (DUF3527)	2	2919.360263	N	N	N	N	N	N	N	M
High	ILCMNKIDLVEK	1xCarbamidomethyl [C3]; 1xOxidation [M4]	1	1	7	Glyma.07G173800.1.p	GTP-BINDING PROTEIN ERG	0	2284.156983	M	N	N	M	N	M	M	N

Confidence	Sequence	Modifications	Protein Groups	Proteins	PSMs	Master Protein Accessions	Description	Missed Cleavages	Theo. MH+ [Da]	atpF_01	atpF_02	ndhB_01	ndhB_02	rps14_01	rps14_02	Control_01	Control_02
Medium	WVERDFLSLSETREASSNR		1	1	1	Glyma.07G174100.1.p	PTHR21736:SF15 - PROTEIN OBERON 3	0	1659.808523	M	N	N	M	N	N	N	N
Medium	FSGQRGLNTGNSR		1	2	1	Glyma.07G187300.1.p	no GO	1	1781.801924	N	N	N	N	N	N	N	M
Medium	MVTWSTWQELLLGGAVSR	1xOxidation [M1]	1	1	1	Glyma.07G192000.1.p	PTHR15398//PTHR15398:SF5 - BROMODOMAIN-CONTAINING PROTEIN 8 // SUBFAMILY NOT NAMED	1	2066.004991	N	M	N	N	N	N	N	N
Medium	YIVGYYNVKQSR		1	1	1	Glyma.07G251600.1.p	no GO	2	4109.124324	N	N	N	N	N	N	M	N
Medium	EEVEFARAEEGVVQNGGNVKEGGEEEK		1	1	1	Glyma.07G251700.1.p	PTHR24056//PTHR24056:SF176 - CELL DIVISION PROTEIN KINASE // SUBFAMILY NOT NAMED	0	3455.617222	N	N	N	N	N	N	N	M
Medium	TTIGELTPFDWLSVYPLGMK	1xOxidation [M19]	1	1	4	Glyma.07G258600.1.p	BETA-GLUCOSIDASE 45-RELATED	2	2242.117243	N	N	N	N	N	N	N	M
Medium	TGLARIGSRLSEAEVK		0	1	1	Glyma.07G260200	CALCIUM-DEPENDENT PROTEIN KINASE 29	0	1851.842919	N	N	N	M	N	N	N	M
Medium	NLPPSAIGDSSNFNAR		1	2	2	Glyma.08G006400.1.p	staphylococcal nuclease domain-containing protein 1 (SND1)	1	2243.08734	N	N	N	H	N	M	N	N
Medium	CIVMLLCWKENGDQ	2xCarbamidomethyl [C1; C7]; 1xOxidation [M4]	1	1	1	Glyma.08G026100.1.p	PF08137 - DVL family (DVL)	0	2330.173044	N	N	N	N	N	N	N	M
Medium	TNPGSVAEVFTGADNRFQR		1	1	1	Glyma.08G063600.1.p	PF03108//PF04434//PF10551 - MuDR family transposase (DBD_Tnp_Mut) // SWIM zinc finger (SWIM) // MULE transposase domain (MULE)	1	2261.095403	N	N	N	M	N	N	N	N
Medium	AVAPPNLPPNFAAVKFHDPVPEPVAPTPVEYQSRVSDR		1	1	1	Glyma.08G065000.1.p	PF00564 - PB1 domain (PB1)	1	1899.890538	N	N	N	N	N	N	N	M
Medium	LAAQATNENSSSSVQIHWYIMTSPFTDEATR		1	1	1	Glyma.08G086700.1.p	UDP-N-acetylgalactosamine diphosphorylase	2	3388.771556	N	N	N	N	N	N	N	H
Medium	GMIYSLLDRNEEAKEQFAK		1	2	1	Glyma.08G090400.1.p	Tetratricopeptide repeat (TPR_2)	1	2050.045124	N	N	N	N	N	N	N	M
Medium	FEKPEEMGIEEDNIR	1xOxidation [M7]	1	2	2	Glyma.08G150000.1.p	K08853 - AP2-associated kinase [EC:2.7.11.1] (AAK)	1	3340.768184	N	N	N	N	M	N	N	N
Medium	EPPPVSPEEEGANASWR		0	1	1	Glyma.08G164700	METAL TOLERANCE PROTEIN 10-RELATED	1	1673.803729	N	N	N	N	N	N	N	M
High	VHLSRDPDGQPYLTQTEMR		1	1	2	Glyma.08G165100.1.p	Transglycosylase SLT domain (SLT)	1	1898.905185	M	N	N	N	N	N	N	N
Medium	MAVTEAQNPLLVENTCGSLLK	1xCarbamidomethyl [C16]; 1xAcetyl [N-Term]	1	1	1	Glyma.08G171100.1.p	sulfate transporter 1, high-affinity (SULTR1)	0	1158.534717	N	N	N	N	N	N	M	N
Medium	LPCVFLDTPGHEAFGAMRAR	1xCarbamidomethyl [C3]; 1xOxidation [M17]	1	1	1	Glyma.08G174200.1.p	TRANSLATION INITIATION FACTOR IF-2, CHLOROPLASTIC	2	1318.714746	N	H	N	N	N	N	N	N

Confidence	Sequence	Modifications	Protein Groups	Proteins	PSMs	Master Protein Accessions	Description	Missed Cleavages	Theo. MH+ [Da]	atpF_01	atpF_02	ndhB_01	ndhB_02	rps14_01	rps14_02	Control_01	Control_02
Medium	FFHKVGSMNASVTDSEK	1xOxidation [M8]	1	1	1	Glyma.08G207800.1.p	peptide alpha-N-acetyltransferase (E2.3.1.88)	0	1405.669295	N	N	N	N	N	N	N	M
High	KPVMKSSPETEKKPVAHSPQCV SVTPPAISR	1xCarbamidomethyl [C21]; 1xOxidation [M4]	1	2	1	Glyma.08G218700.1.p	DNA-3-methyladenine glycosylase I / DNA-3-methyladenine glycosidase I	2	1618.780636	N	N	N	N	N	N	N	H
Medium	TSPSRIEDAEFVNSLLGSK		1	4	1	Glyma.08G231800.1.p	Remorin, C-terminal region (Remorin_C)	1	1390.680863	N	N	N	N	N	N	M	N
Medium	FLDASGNQITGPIPVGLGDMVS LVSLNLSRNR		1	1	1	Glyma.08G246100.1.p	LRR RECEPTOR-LIKE SERINE/THREONINE-PROTEIN KINASE RPK2	0	1613.820333	H	N	N	N	N	N	N	M
Medium	MPRGHGSSNGVNHAPR		1	1	1	Glyma.08G246500.1.p	no GO	2	2494.104707	N	N	N	N	N	N	N	M
Medium	ESFAAEMELEKSSLAEK		1	3	2	Glyma.08G256300.1.p	PROTEIN LITTLE NUCLEI1	1	2083.017335	H	M	M	M	H	H	N	H
Medium	AELATYMESK	1xOxidation [M7]	1	1	1	Glyma.08G266500.2.p	PROTEIN WEAK CHLOROPLAST MOVEMENT UNDER BLUE LIGHT 1-RELATED	2	2227.107213	M	N	N	N	H	N	N	N
High	ACAKLTDLNKGK	1xCarbamidomethyl [C2]	1	1	1	Glyma.08G291800.1.p	DEAD/DEAH box helicase (DEAD) // Helicase conserved C-terminal domain (Helicase_C) // PPR repeat (PPR) // PPR repeat family (PPR_2) // DYW family of nucleic acid deaminases (DYW_deaminase)	1	1546.807126	N	N	N	N	N	N	N	M
Medium	GVSEDDVEVTLDK		1	3	1	Glyma.08G300100.1.p	cullin 3 (CUL3)	1	4276.064896	N	N	N	N	N	M	N	N
High	EDKEVDGVEVKEDK		1	1	1	Glyma.08G329100.2.p	PTHR13468:SF1 - PROTEIN DEK	0	2050.009607	M	H	N	N	N	N	N	N
Medium	ITKAHDSESSTSK		1	1	1	Glyma.08G358900.1.p	PTHR33142:SF6 - EXPRESSED PROTEIN	2	2918.455248	N	N	N	M	N	N	N	N
High	EQNNLLADLMDIPK		1	2	2	Glyma.09G008800.1.p	K12483 - EH domain-containing protein 1 (EHD1)	0	2898.444802	N	N	N	N	N	N	N	H
Medium	MSMSERKTIDLDQGWDFMQK	3xOxidation [M1; M3; M18]	1	2	1	Glyma.09G046500.1.p	K03347 - cullin 1 (CUL1, CDC53)	0	1371.697516	N	N	N	N	N	N	N	M
High	MTLWSMAKAPLMYAGDVR	1xAcetyl [N-Term]	1	1	18	Glyma.09G095100.1.p	Alpha-N-acetylgalactosaminidase / Alpha-NAGA	0	2314.207121	N	N	N	N	N	M	N	N
High	MTLWSMAKAPLMYAGDVRK	1xOxidation [M6]; 1xAcetyl [N-Term]	1	1	2	Glyma.09G095100.1.p	Alpha-N-acetylgalactosaminidase / Alpha-NAGA	1	2243.086872	H	N	N	H	M	M	N	H
Medium	ETAANIGASAKSGLEK		1	1	1	Glyma.09G112100.1.p	LATE EMBRYOGENESIS ABUNDANT PROTEIN 4-5	2	4258.998122	N	N	N	N	N	M	N	N
Medium	EDDPDMLADVKQMAAAAAAA GQAISAFENELRPIDQYAIR		1	8	1	Glyma.09G112200.5.p	E1A-binding protein p400 [EC:3.6.4.-] (EP400)	1	1858.79372	N	N	N	H	N	M	N	N
High	MIVEVAELAFQCLQQDR	1xCarbamidomethyl [C12]	1	3	3	Glyma.09G116300.1.p	Protein kinase domain (Pkinase) // Wall-associated	2	2083.020306	N	M	M	M	H	M	N	M

Confidence	Sequence	Modifications	Protein Groups	Proteins	PSMs	Master Protein Accessions	Description	Missed Cleavages	Theo. MH+ [Da]	atpF_01	atpF_02	ndhB_01	ndhB_02	rps14_01	rps14_02	Control_01	Control_02
							receptor kinase galacturonan-binding (GUB_WAK_bind) // Wall-associated receptor kinase C-terminal (WAK_assoc)										
Medium	ILHSRRLFHQWVVDGYCMIESQK	1xCarbamidomethyl [C17]; 1xOxidation [M18]	1	1	1	Glyma.09G117700.1.p	DNA helicase	2	2257.099619	N	N	N	M	N	N	N	N
High	AQAIMLLAGNGLSAGSNEASPNVQAPCLK	1xCarbamidomethyl [C27]; 1xOxidation [M5]	1	1	1	Glyma.09G123600.1.p	PROTEIN TIFY 6A-RELATED	2	2096.132106	N	N	N	N	N	N	N	M
Medium	QNGASIEGNGVLGR		1	2	1	Glyma.09G160900.1.p	CYTOCHROME P450 SUPERFAMILY PROTEIN	2	2225.094534	N	M	N	N	N	N	N	N
Medium	LNMESVTTVNSLIGNTVQHIK	1xOxidation [M3]	1	2	1	Glyma.09G168000.2.p	HOMEOBOX-LEUCINE ZIPPER PROTEIN HDG12	0	1859.819481	N	N	N	N	N	N	N	M
High	YIISAMSPSRVNPMMQLEK	3xOxidation [M6; M14; M15]	1	2	11	Glyma.09G170700.1.p	N-ALPHA-ACETYLTRANSFERASE 40	1	4571.137468	M	N	N	N	N	N	N	N
Medium	EEIQEMMDFAAKHNITADVEVIPMDYVNTAFERLEK	2xOxidation [M7; M24]	1	1	1	Glyma.09G201200.1.p	CINNAMYL ALCOHOL DEHYDROGENASE 6-RELATED	0	1491.735935	H	N	M	N	N	N	N	N
High	SDVYDKDDEEQSATTR		1	1	5	Glyma.09G203800.1.p	Organic Anion Transporter Polypeptide (OATP) family (OATP)	2	3048.473994	N	N	M	N	N	N	N	N
High	NANSFTLRGEDGPSNKFTK		1	1	16	Glyma.09G225200.1.p	ubiquitin carboxyl-terminal hydrolase 10 [EC:3.4.19.12] (USP10, UBP3)	2	4259.027983	N	N	N	N	N	N	M	N
Medium	TKNYFTDHLNAIYESERR		1	1	1	Glyma.09G280400.1.p	Soyasapogenol B glucuronide galactosyltransferase	1	2409.04904	N	N	M	N	N	N	N	N
Medium	EQQSLMVPLREKFIHPK	1xOxidation [M6]	1	3	1	Glyma.09G282200.1.p	K17592 - sacsin (SACS)	1	2239.127474	N	N	M	N	N	M	N	H
Medium	SQIYDGNANRNNVEVFKEK		1	1	1	Glyma.10G000400.2.p	Sieve element occlusion N-terminus (SEO_N) // Sieve element occlusion C-terminus (SEO_C)	0	1689.799992	N	N	N	M	N	N	N	M
Medium	FSFESVSHESTSSWSR		1	1	2	Glyma.10G006300.1.p	REGULATOR OF CHROMOSOME CONDENSATION REPEAT-CONTAINING PROTEIN-RELATED	2	1491.77232	N	N	H	M	N	N	N	N
Medium	QDVRHLKDVSLWNQNFDK		0	2	1	Glyma.10G008200	GENOMIC DNA, CHROMOSOME 3, TAC CLONE:K14B15	1	1899.900434	H	N	H	N	N	N	N	N
Medium	GDFELGEIPSLMDLGLNPSATMSQDGKFGANASMIGGETEALQR	1xOxidation [M22]	1	1	1	Glyma.10G017900.1.p	DNA photolyase / DNA photolyase	1	1865.829394	N	N	M	N	M	N	N	H

Confidence	Sequence	Modifications	Protein Groups	Proteins	PSMs	Master Protein Accessions	Description	Missed Cleavages	Theo. MH+ [Da]	atpF_01	atpF_02	ndhB_01	ndhB_02	rps14_01	rps14_02	Control_01	Control_02
High	TDCSLTEQLLPSK	1xCarbamidomethyl [C3]	1	3	5	Glyma.10G023700.1.p	KOG1893 - Uncharacterized conserved protein	2	3347.66147	N	N	N	N	M	N	N	N
Medium	AQLFGPMKNQNTHCIKVEDME APVFK	1xCarbamidomethyl [C14]; 1xOxidation [M7]	1	3	1	Glyma.10G024300.1.p	K10523 - speckle-type POZ protein (SPOP)	1	2239.155301	H	N	N	N	N	N	N	N
Medium	MVGCEGLWGVLMKSSGTTELN TFYPIKAECQADVPATR	2xCarbamidomethyl [C4; C30]; 1xAcetyl [N-Term]	1	2	1	Glyma.10G038700.1.p	RABGAP/TBC DOMAIN-CONTAINING PROTEIN	1	1316.699096	N	N	N	N	N	M	N	N
Medium	EEPAKGSAPHNNDDWAGWDN AK		1	1	1	Glyma.10G043600.1.p	ADP-ribosylation factor GTPase-activating protein 1 (ARFGAP1)	1	1865.835919	N	N	N	N	H	M	N	N
High	LTVTEYERQQVMDESGILK		1	4	5	Glyma.10G062800.1.p	Rab5 GTPase effector Rabaptin-5 // Mitotic checkpoint protein MAD1	0	1841.806928	N	N	N	M	N	N	N	N
Medium	EAQFSSPNLNVAYKMNVR	1xOxidation [M15]	0	1	1	Glyma.10G097800	magnesium chelatase subunit H (chlH, bchH)	2	1996.939279	N	M	N	N	N	N	N	N
Medium	SSMVYAATTPTSSTGTK		1	1	2	Glyma.10G100700.1.p	no GO	0	1557.797958	N	N	N	N	N	N	N	M
High	MEDKLKTELEQK		1	1	8	Glyma.10G100900.1.p	PF09731 - Mitochondrial inner membrane protein (Mitofilin)	1	1373.731794	M	N	N	N	N	N	M	M
High	MNESKDLYDVELVSNK	1xOxidation [M1]	1	1	2	Glyma.10G105200.1.p	TAG lipase / steryl ester hydrolase / phospholipase A2 / LPA acyltransferase (TGL4)	2	1826.827227	N	N	N	N	N	N	N	M
High	ACQSKQSDQSLQEDNK	1xCarbamidomethyl [C2]	1	2	4	Glyma.10G196400.1.p	PTHR34373:SF2 - SHUGOSHIN C TERMINUS	1	1261.656897	N	M	N	N	N	N	N	N
Medium	NSYNAINHGISVRLLEEQLATA ESGDDFKR		1	8	1	Glyma.10G201800.1.p	SENTRIN/SUMO-SPECIFIC PROTEASE // SUBFAMILY NOT NAMED	1	1361.735816	N	N	N	N	N	N	M	M
High	AFSHYLTLMGIAETHHRVR		1	1	1	Glyma.10G205500.1.p	phosphoenolpyruvate carboxylase (ppc)	2	2225.130919	N	N	N	M	N	N	N	N
Medium	AGADDKPLRMVK	1xOxidation [M10]	1	4	1	Glyma.10G207500.1.p	cytoskeleton-associated protein 5 (CKAP5, XMAP215)	1	2050.008739	M	N	N	N	N	N	N	N
High	TEAEVQSSENASDAKGDK		1	1	2	Glyma.10G229400.1.p	AAA ATPASE // SUBFAMILY NOT NAMED	2	4081.98452	N	N	N	N	N	N	N	M
Medium	MSNNSDGSVLESSELEK	1xOxidation [M1]	1	1	1	Glyma.10G264200.1.p	CWF19-LIKE PROTEIN 2	1	1367.677632	N	N	N	N	N	N	M	N
Medium	STTTEETNIQDSGWNK		0	1	1	Glyma.10G276900	KOW DOMAIN-CONTAINING TRANSCRIPTION FACTOR 1	1	2170.025845	M	M	N	N	M	H	N	N
Medium	VSQFNGDKCSAKSDPTQK	1xCarbamidomethyl [C9]	1	3	1	Glyma.11G001900.1.p	DNA polymerase kappa (POLK)	1	2243.119887	N	N	N	M	N	N	N	N
Medium	SGGGVVITAVDGGGNAAR		1	2	1	Glyma.11G012700.1.p	MITOCHONDRIAL OUTER MEMBRANE PROTEIN 25	2	2284.196556	M	N	N	N	N	N	N	N
Medium	MRAAAIEQQIAR	1xOxidation [M1]	1	2	3	Glyma.11G078600.2.p	Myb/SANT-like DNA-binding domain (Myb_DNA-bind_3)	0	1843.798295	N	N	N	N	N	H	N	N

Confidence	Sequence	Modifications	Protein Groups	Proteins	PSMs	Master Protein Accessions	Description	Missed Cleavages	Theo. MH+ [Da]	atpF_01	atpF_02	ndhB_01	ndhB_02	rps14_01	rps14_02	Control_01	Control_02
Medium	KRIECCGEFEQNTR	2xCarbamidomethyl [C5; C6]	1	1	1	Glyma.11G091300.1.p	INHIBITOR OF APOPTOSIS // SUBFAMILY NOT NAMED	1	1061.54733	M	N	N	N	N	N	N	N
Medium	DCIKAAISAQGK	1xCarbamidomethyl [C2]	1	2	1	Glyma.11G103800.1.p	C3HC4 ZINC FINGER DOMAIN-CONTAINING PROTEIN-RELATED	1	2905.375994	N	N	N	N	N	N	N	M
Medium	RANVQFLSAPMK		1	1	3	Glyma.11G108900.1.p	SUGAR KINASE // SUBFAMILY NOT NAMED	0	1509.743129	N	N	N	N	N	N	N	H
Medium	MGDKLMVLFTNLPSSREFR		0	1	1	Glyma.11G111200	PPR repeat (PPR) // PPR repeat family (PPR_2)	2	2275.135336	N	N	N	M	N	M	N	N
Medium	VEADNGSRYVQLANAYAAKG K		1	1	1	Glyma.11G121500.1.p	PPR repeat (PPR) // PPR repeat family (PPR_2)	0	2411.179903	N	N	N	N	N	N	N	M
Medium	IAEVHAAEDKSQEDSVPPK		1	1	1	Glyma.11G128300.1.p	TAT-BINDING HOMOLOG 7	2	3064.51903	M	N	N	N	N	N	N	N
Medium	MAASTTSQVYIQVIDDVMNKV RDEFVNNGGPGDEVLK	1xAcetyl [N-Term]	1	1	1	Glyma.11G136100.1.p	transcription initiation factor TFIIA large subunit (TFIIA1, GTF2A1, TOA1)	0	2207.108653	N	N	N	N	N	M	N	N
Medium	EDFRNFLPAMK		1	4	1	Glyma.11G145200.1.p	GLYCOSYLTRANSFERASE FAMILY PROTEIN 2	0	1843.827938	N	N	N	N	N	M	N	H
High	IYKTDGLADASTQTEENASR		1	2	5	Glyma.11G193000.3.p	PF06136 - Domain of unknown function (DUF966) (DUF966)	1	1766.78747	N	N	N	N	N	N	N	M
Medium	KETLMMSNTLHNFIIQYK	2xOxidation [M5; M6]	1	1	1	Glyma.11G202400.1.p	no GO	1	1658.80291	M	N	N	N	N	N	N	H
Medium	YAEDMIGRGISLSSSTLSKLR		1	1	1	Glyma.11G217500.1.p	PPR repeat (PPR) // PPR repeat family (PPR_2)	0	1384.670298	M	N	N	N	N	N	N	N
High	MSTATLSHQMESMTTR	2xOxidation [M1; M10]	1	1	1	Glyma.11G223200.1.p	Protein tyrosine kinase (Pkinase_Tyr) // Wall-associated receptor kinase galacturonan-binding (GUB_WAK_bind)	2	2211.133896	N	N	N	H	M	H	H	H
Medium	AAIKEESEGK		1	1	1	Glyma.11G247600.1.p	GLYCERALDEHYDE-3-PHOSPHATE DEHYDROGENASE GAPC1, CYTOSOLIC	2	2227.128811	N	N	N	N	H	N	N	N
Medium	VSYGGRHNLNSGGGMAVMAE DTFFFLK		1	1	1	Glyma.11G256900.1.p	BED zinc finger (zf-BED) // Protein of unknown function (DUF 659) (DUF659)	1	1433.695408	N	N	N	N	N	N	N	M
High	LYANITDDSADALK		1	1	1	Glyma.12G136700.1.p	LEUCINE-RICH REPEAT-CONTAINING PROTEIN // SUBFAMILY NOT NAMED	2	2083.019838	N	M	N	N	N	N	N	N
Medium	SGSFQFNEEMIETLFGYNAVDK		0	2	1	Glyma.12G136800	FORMIN-LIKE PROTEIN 12-RELATED	2	2392.171404	H	N	N	N	N	N	N	N
Medium	ATENFSTKNKLGEGGFGQVYK		1	1	2	Glyma.12G144200.1.p	Protein tyrosine kinase (Pkinase_Tyr) // PAN-like domain (PAN_2)	1	1808.807931	N	M	N	N	N	N	N	N

Confidence	Sequence	Modifications	Protein Groups	Proteins	PSMs	Master Protein Accessions	Description	Missed Cleavages	Theo. MH+ [Da]	atpF_01	atpF_02	ndhB_01	ndhB_02	rps14_01	rps14_02	Control_01	Control_02
Medium	TLVDDEELMNLLFSFLEPNR	1xOxidation [M9]	1	3	1	Glyma.12G174000.1.p	serine/threonine-protein phosphatase 6 regulatory subunit 3 (PPP6R3, SAPS3)	2	2226.152189	N	N	N	M	N	N	N	N
Medium	CTVETHAEYILDLQKQLSLMGS EARR	1xCarbamidomethyl [C1]; 1xOxidation [M20]	1	1	1	Glyma.12G201100.1.p	VACUOLAR PROTEIN SORTING-ASSOCIATED PROTEIN 18 HOMOLOG	1	2084.015087	N	N	N	N	H	H	N	N
Medium	SEMPLDVLVCNAAVYLPTAK	1xCarbamidomethyl [C10]; 1xOxidation [M3]	1	2	1	Glyma.12G222200.1.p	protochlorophyllide reductase (E1.3.1.33, por)	2	1316.716855	N	N	N	N	N	N	M	M
High	LADFGVSACLYDNAGDR	1xCarbamidomethyl [C9]	1	5	2	Glyma.13G001900.1.p	Ste20-like serine/threonine protein kinase	1	2026.002214	N	N	N	N	N	N	N	M
Medium	YNSVESHMADSQSRR		1	3	1	Glyma.13G009900.1.p	TAF RNA Polymerase I subunit A (TAF1_subA)	1	1810.819741	N	M	M	H	M	M	N	M
High	FLDFGLLCQMDKR	1xCarbamidomethyl [C8]; 1xOxidation [M10]	1	1	2	Glyma.13G043700.1.p	ATH SUBFAMILY PROTEIN ATH8	1	1413.657776	N	N	N	M	N	N	N	N
Medium	SSNSSLSLSYPSR		1	2	1	Glyma.13G048800.1.p	endo-1,3(4)-beta-glucanase (E3.2.1.6)	2	2225.072753	N	M	N	N	N	N	N	N
High	GKSNSNQIFGGKVMVLGGDFR		1	1	10	Glyma.13G076000.1.p	DNA helicase	1	2083.005919	M	M	H	M	H	H	N	H
High	GKSNSNQIFGGKVMVLGGDFR	1xOxidation [M14]	1	1	1	Glyma.13G076000.1.p	DNA helicase	2	4107.949181	N	N	M	N	N	N	N	N
Medium	MALNKNHSYNNK		1	1	1	Glyma.13G122400.1.p	E3 ubiquitin-protein ligase RNF38/44 [EC:6.3.2.19] (RNF38_44)	2	4194.044161	N	N	M	N	N	N	N	N
Medium	FMDAVVVDNEKTGKECIK	1xCarbamidomethyl [C16]	1	2	1	Glyma.13G123700.1.p	structural maintenance of chromosome 1 (SMC1)	1	2277.129205	N	N	N	N	N	M	N	N
High	MWNWLLTAEGQANTQKNDKK	1xOxidation [M1]	1	2	2	Glyma.13G158400.1.p	no GO	1	2261.12708	N	N	N	M	N	N	N	N
Medium	GENMELGRDSDTGGQVK	1xOxidation [M4]	1	4	1	Glyma.13G161600.1.p	GLYCOSYLTRANSFERASE // SUBFAMILY NOT NAMED	1	1842.820804	N	N	N	N	N	H	N	N
Medium	FAKMEGITAGLCFKGQSKPR	1xCarbamidomethyl [C12]	1	6	1	Glyma.13G221700.6.p	no GO	1	1826.825889	N	N	N	M	M	M	N	N
High	GSPMARDFSGAILTLAEMGK	2xOxidation [M4; M18]	1	1	4	Glyma.13G233300.1.p	IONOTROPIC GLUTAMATE RECEPTOR // SUBFAMILY NOT NAMED	1	1810.830974	N	N	N	M	N	N	N	N
Medium	VTDLDDNLKRK		1	1	2	Glyma.13G253200.1.p	no GO	2	3310.778932	N	N	N	N	N	N	N	M
Medium	SNIVHQPSKNIACDSIDK	1xCarbamidomethyl [C13]	1	1	1	Glyma.13G268300.1.p	DNA polymerase subunit Cdc27 (CDC27)	2	1318.714746	N	M	N	M	N	N	N	M
High	MVGTEAAEKMSVNEEK	1xOxidation [M10]; 1xAcetyl [N-Term]	1	1	18	Glyma.13G283500.1.p	PF08387 - FBD (FBD)	0	1340.706959	N	N	N	N	N	N	N	M
Medium	SSNHASRNNNQGK		1	2	1	Glyma.13G294300.1.p	TRANSCRIPTION ACTIVATOR GLK1-RELATED	1	1523.755625	N	N	N	N	N	N	N	H
Medium	NKGTGHPQLKDITCGQADER	1xCarbamidomethyl [C14]	1	1	1	Glyma.13G295400.1.p	SQUAMOSA PROMOTER-BINDING-LIKE PROTEIN 4-RELATED	2	2239.171722	N	N	N	N	N	N	N	H

Confidence	Sequence	Modifications	Protein Groups	Proteins	PSMs	Master Protein Accessions	Description	Missed Cleavages	Theo. MH+ [Da]	atpF_01	atpF_02	ndhB_01	ndhB_02	rps14_01	rps14_02	Control_01	Control_02
High	QGSNLFMGNIGDSRAIMGSK		1	2	22	Glyma.13G299300.5.p	PROTEIN PHOSPHATASE 2C 1-RELATED	0	2269.160688	N	N	N	N	N	M	N	N
Medium	GKLADFGLSKIYSDEDDTHMTT VVAGTLGYLDPEYNR	1xOxidation [M20]	1	2	1	Glyma.13G352700.1.p	LRR RECEPTOR-LIKE SERINE/THREONINE-PROTEIN KINASE MEE39-RELATED	0	1764.865035	N	N	N	N	N	M	N	N
Medium	NNLSGSIPSTLVEKSKEGSLALS VGQNPYLCESGQCNQK	2xCarbamidomethyl [C31; C36]	1	1	1	Glyma.13G352800.1.p	LEUCINE-RICH REPEAT PROTEIN KINASE-RELATED	1	1361.731793	N	N	N	M	N	N	M	N
Medium	NGKYGVGGICNGGGGASALVV ELQ	1xCarbamidomethyl [C10]	1	2	1	Glyma.14G004600.1.p	Acetyl-CoA C-acetyltransferase / Acetoacetyl-CoA thiolase	1	2084.036868	M	M	H	N	H	H	N	N
Medium	LGHDSLVDGMLKDGLWDVYK		1	4	1	Glyma.14G004600.1.p	Acetyl-CoA C-acetyltransferase / Acetoacetyl-CoA thiolase	2	1491.801312	N	N	N	N	N	N	N	M
Medium	SRQNTGEGGGEAFLGDEK		0	1	1	Glyma.14G036500	CLAVATA3/ESR (CLE)-RELATED PROTEIN 21-RELATED	1	2393.292231	N	N	N	N	M	N	N	N
High	SDSEVEEMLDRMLTR	2xOxidation [M8; M12]	1	1	2	Glyma.14G036800.1.p	proteasome component ECM29 (ECM29)	1	1850.822335	N	N	N	M	N	N	N	N
Medium	SDSEVEEMLDRMLTR	1xOxidation [M12]	1	1	10	Glyma.14G036800.1.p	proteasome component ECM29 (ECM29)	1	1491.739775	N	N	N	N	N	N	N	M
Medium	SDSEVEEMLDRMLTR		1	1	1	Glyma.14G036800.1.p	proteasome component ECM29 (ECM29)	1	1964.923612	M	N	N	N	N	N	N	N
Medium	IMDLITHPSPVLFDAIKSGNVEA VKMLIDK	1xOxidation [M2]	1	1	1	Glyma.14G038400.1.p	Ankyrin repeats (many copies) (Ank_4) // Domain of unknown function (PGG)	1	2253.126703	N	N	M	N	N	N	N	N
Medium	AMKEKATEIAAR		1	1	3	Glyma.14G043700.1.p	GLUCOSYL/GLUCURONOS YL TRANSFERASES // SUBFAMILY NOT NAMED	0	1613.842115	N	N	N	N	N	N	N	M
Medium	GGFPVGGGVGLPGGGR		1	1	1	Glyma.14G052500.1.p	no GO	2	2409.208501	N	N	N	N	N	N	N	M
High	KMMRPSISPTTDK	2xOxidation [M2; M3]	1	2	3	Glyma.14G094800.1.p	TRANSCRIPTION FACTOR GATA GATA BINDING FACTOR // SUBFAMILY NOT NAMED	0	2216.906584	N	N	N	N	N	N	N	M
High	LSDTNYVPTKEDVLYARVR		1	2	2	Glyma.14G098000.1.p	guanine nucleotide-binding protein subunit alpha, other (GNA)	2	2393.166002	N	N	N	N	N	N	N	M
Medium	LFNMINDLPTIFEVVTGMAK	1xOxidation [M18]	1	4	2	Glyma.14G099700.1.p	PHD FINGER PROTEIN ALFIN-LIKE 3	0	1393.699156	N	N	N	N	N	N	H	N
Medium	ESSVSGLTIISENDWK		1	1	1	Glyma.14G132500.1.p	ubiquitin carboxyl-terminal hydrolase 48 [EC:3.4.19.12] (USP48)	0	1780.826935	N	N	N	N	N	N	N	M
Medium	ARGGCGTTILSLR	1xCarbamidomethyl [C5]	1	1	2	Glyma.14G159400.1.p	JMJC DOMAIN-CONTAINING HISTONE	0	1122.517427	N	N	N	N	N	N	N	M

Confidence	Sequence	Modifications	Protein Groups	Proteins	PSMs	Master Protein Accessions	Description	Missed Cleavages	Theo. MH+ [Da]	atpF_01	atpF_02	ndhB_01	ndhB_02	rps14_01	rps14_02	Control_01	Control_02
							DEMETHYLATION PROTEIN // SUBFAMILY NOT NAMED										
High	MEGYEILKDIGSGNFAVAK	1xAcetyl [N-Term]	1	1	7	Glyma.14G176700.1.p	SERINE/THREONINE-PROTEIN KINASE SRK2C	0	2872.437198	N	N	N	N	N	N	N	M
Medium	GTAKEVASSVGETKK		1	1	1	Glyma.14G179900.1.p	no GO	1	2225.134942	N	M	M	N	N	N	N	N
Medium	EGVGLEITTELRLGLPGGELPDK		1	1	1	Glyma.15G012800.1.p	AUXIN-RESPONSIVE PROTEIN IAA19-RELATED	1	1782.799674	N	M	N	N	N	H	N	N
Medium	RTQGDDAANSGSGSDSGLR		1	1	1	Glyma.15G023900.1.p	no GO	2	2243.097236	N	N	N	N	N	H	N	H
Medium	SESGHLHKSAYSDSGK		0	3	1	Glyma.15G035900	pumilio RNA-binding family (PUM)	1	1422.722334	N	N	N	N	N	N	N	M
Medium	TTTTDGELDNIRR		1	1	1	Glyma.15G087100.1.p	carboxy-terminal domain RNA polymerase II polypeptide A small phosphatase [EC:3.1.3.16] (CTDSP)	0	1423.70787	N	N	N	M	N	N	N	N
Medium	EEADFQLVDENKVDWK		1	1	1	Glyma.15G088200.1.p	OLIGOPEPTIDE TRANSPORTER-RELATED // SUBFAMILY NOT NAMED	0	2267.160294	M	N	N	N	N	N	N	N
Medium	GPHMVVAPKSTLGNWMNEIR	1xOxidation [M4]	1	2	1	Glyma.15G097000.1.p	SWI/SNF-related matrix-associated actin-dependent regulator of chromatin subfamily A member 5 [EC:3.6.4.-] (SMARCA5, SNF2H, ISWI)	2	1876.837985	N	N	N	N	N	N	N	M
Medium	LEEVLSDPTLPWSK		1	1	1	Glyma.15G107100.1.p	MATE EFFLUX FAMILY PROTEIN	1	2920.417918	N	N	N	N	N	N	N	M
Medium	FLHYDKPEEKFQDTKDQLK		1	1	1	Glyma.15G107500.1.p	PTHR31071:SF14 - F25C20.16	2	4534.368579	N	N	N	N	N	M	N	N
Medium	ELMEEMCEELAMQIGEDK	1xCarbamidomethyl [C7]; 2xOxidation [M3; M6]	1	2	1	Glyma.15G107500.1.p	PTHR31071:SF14 - F25C20.16	0	3295.538304	N	N	N	N	N	N	M	N
Medium	VQEAMASIAASAEADRNAMRI R	2xOxidation [M5; M19]	1	1	1	Glyma.15G139900.1.p	Huntingtin interacting protein HYPK	2	2213.117184	N	N	H	N	N	M	N	H
High	QSTTSIIQMGEAK		1	2	1	Glyma.15G167400.1.p	large subunit ribosomal protein L7e (RP-L7e, RPL7)	1	2225.065542	N	N	M	N	N	N	N	N
Medium	LLTELMDADSDEVGTR	1xOxidation [M6]	1	1	2	Glyma.15G179000.1.p	PTHR12000:SF3 - LEGUMAIN	0	1866.92996	N	N	N	N	N	N	N	M
Medium	AEYVQQNDR		1	2	1	Glyma.15G237600.3.p	no GO	1	1547.803896	N	N	N	N	N	N	N	H
Medium	DDMWVPVRPMPNAFVVNVGDI LEVK	2xOxidation [M3; M10]	1	1	1	Glyma.15G240600.2.p	OXIDOREDUCTASE, 2OG-FE II OXYGENASE FAMILY PROTEIN // SUBFAMILY NOT NAMED	0	1695.879956	N	N	M	N	N	N	N	N
Medium	EEIHEPQWLGKTNFVEIR		1	2	2	Glyma.15G245800.1.p	CALLOSE SYNTHASE 8-RELATED	1	1811.808934	N	N	N	H	N	N	N	N

Confidence	Sequence	Modifications	Protein Groups	Proteins	PSMs	Master Protein Accessions	Description	Missed Cleavages	Theo. MH+ [Da]	atpF_01	atpF_02	ndhB_01	ndhB_02	rps14_01	rps14_02	Control_01	Control_02
High	CMQKESVSETLADER	1xCarbamidomethyl [C1]	1	2	2	Glyma.15G251900.2.p	GLUTATHIONE S-TRANSFERASE, GST, SUPERFAMILY, GST DOMAIN CONTAINING // SUBFAMILY NOT NAMED	1	1615.817356	N	N	M	N	N	N	N	N
High	AAGHITKKTDVMDDDDLIER		1	1	3	Glyma.15G258300.1.p	cyclin T (CCNT)	0	1372.69545	N	N	N	N	N	N	N	M
Medium	TAEDFLRSATPSK		1	4	1	Glyma.15G273700.1.p	no GO	1	2066.004991	M	N	N	N	N	N	N	N
Medium	VGDYFMFYLLR		1	9	1	Glyma.15G274000.1.p	telomerase reverse transcriptase (TERT, EST2)	2	1851.851467	N	N	N	N	N	N	N	M
Medium	MPFLQMLQSVESPQFFPLK		1	2	1	Glyma.16G017700.2.p	TRANSCRIPTION FACTOR BHLH57-RELATED	1	1615.826088	N	N	N	M	N	N	N	N
Medium	NQKQESGNNRGESEDGK		1	1	2	Glyma.16G030000.1.p	BAG FAMILY MOLECULAR CHAPERONE REGULATOR 6	2	2243.08734	N	M	N	N	N	N	N	N
Medium	MEKEVVESLWNGNTEMQIQAAVELR	1xOxidation [M1]	1	1	1	Glyma.16G070300.1.p	ARMADILLO/BETA-CATENIN-LIKE REPEAT-CONTAINING PROTEIN	2	1395.752529	N	N	N	N	N	N	N	M
Medium	MIDDTLEAFFLLPIPMHADLLPGLMSGLDKSLQQYILKAK	3xOxidation [M1; M16; M25]	1	2	1	Glyma.16G077200.1.p	Plant family of unknown function (DUF810) (DUF810)	2	2225.120555	N	N	M	N	M	N	N	N
Medium	ENHGTLPSEDNTVQDPVLMASGEDNIVELR	1xOxidation [M19]	1	21	1	Glyma.16G093800.38.p	Nucleoplasmin ATPase	1	2066.016224	M	N	N	N	N	N	N	N
High	IGYCWPTLRADTLDFTRK	1xCarbamidomethyl [C4]	1	1	4	Glyma.16G120100.1.p	no GO	1	1409.727085	N	N	N	N	N	N	N	H
Medium	NTGAGEWSGEEGLMVLSFGRK		1	1	1	Glyma.16G122900.1.p	no GO	2	2083.0277	N	N	N	M	N	N	N	N
Medium	MAASTMALSSSSLAGQAIK	1xAcetyl [N-Term]	1	3	1	Glyma.16G165200.1.p	light-harvesting complex II chlorophyll a/b binding protein 1 (LHCB1)	0	2211.122663	N	N	N	N	H	N	N	N
High	SCKLGPTFPNWLK	1xCarbamidomethyl [C2]	1	1	2	Glyma.16G171400.1.p	Leucine Rich Repeat (LRR_1) // Leucine rich repeat N-terminal domain (LRRNT_2) // Leucine rich repeat (LRR_8)	1	1817.797031	N	N	N	N	H	N	N	N
Medium	ALENTAIPIGDLPEDK		1	3	1	Glyma.16G182500.1.p	tripeptidyl-peptidase II [EC:3.4.14.10] (TPP2)	0	2172.02776	N	N	N	M	N	N	N	N
High	MGISWSNSNSNSSSRR	1xAcetyl [N-Term]	1	1	1	Glyma.17G016900.1.p	E3 UBIQUITIN-PROTEIN LIGASE LUL3-RELATED	0	2082.040497	N	N	N	N	N	N	N	H
Medium	DDIELALNEVNKDK		1	2	1	Glyma.17G029600.1.p	pumilio homology domain family member 6 (PUF6)	1	2082.013824	M	N	N	N	N	N	N	N
Medium	DDIELALNEVNK		1	2	1	Glyma.17G029600.1.p	pumilio homology domain family member 6 (PUF6)	0	1490.767176	N	N	H	N	N	N	N	M
Medium	FDAPGTRHPSNITDPIGDR		1	4	1	Glyma.17G035500.1.p	FLOWERING TIME CONTROL PROTEIN FCA-RELATED	2	4200.071285	N	N	N	N	N	N	N	M
Medium	KTANESNSGHARCYTR	1xCarbamidomethyl [C13]	1	1	1	Glyma.17G061000.1.p	SPERMIDINE HYDROXYCINNAMOYL TRANSFERASE	1	2239.102321	N	N	N	N	N	H	N	N

Confidence	Sequence	Modifications	Protein Groups	Proteins	PSMs	Master Protein Accessions	Description	Missed Cleavages	Theo. MH+ [Da]	atpF_01	atpF_02	ndhB_01	ndhB_02	rps14_01	rps14_02	Control_01	Control_02
Medium	EEFLRLHSSCIPK	1xCarbamidomethyl [C10]	1	1	1	Glyma.17G071400.1.p	CGTHBA PROTEIN -14 GENE PROTEIN	2	2050.01747	M	M	N	N	N	N	N	N
Medium	RPSYGETSMKRSFGQEPGTK		1	1	1	Glyma.17G076300.1.p	PTHR31471:SF8 - GB	1	2282.096902	N	N	N	M	N	N	N	N
Medium	TRLKQFSVMNR	1xOxidation [M9]	1	1	1	Glyma.17G096000.1.p	CALCIUM-DEPENDENT PROTEIN KINASE 10-RELATED	1	2691.288032	N	N	M	N	N	N	N	N
Medium	FLIRMGLSGKAWDNMLDNK	1xOxidation [M5]	1	1	3	Glyma.17G103700.1.p	ATPASE 6, PLASMA MEMBRANE-TYPE	1	2009.983016	M	N	N	M	M	H	H	H
Medium	KLPHAHSDPVVQDQHDSR		1	4	1	Glyma.17G105100.1.p	GENOMIC DNA, CHROMOSOME 3, TAC CLONE:K7P8-RELATED	1	1851.862233	N	N	N	N	N	N	N	M
High	ESAKGALSTPSFSK		1	1	1	Glyma.17G148100.1.p	no GO	2	2333.197166	N	N	N	M	N	N	N	N
Medium	MVSPRSFVDSFLDAKEGR	1xAcetyl [N-Term]	1	1	1	Glyma.17G190100.1.p	CORE-2/I-BRANCHING BETA-1,6-N-ACETYLGLUCOSAMINYLT RANSFERASE FAMILY PROTEIN	1	2239.124972	N	M	M	H	M	M	N	N
High	IIHCDIKPENVLLDDNFR	1xCarbamidomethyl [C4]	1	1	1	Glyma.17G211200.1.p	G-TYPE LECTIN S-RECEPTOR-LIKE SERINE/THREONINE-PROTEIN KINASE SD2-5	1	1395.722668	N	N	N	M	N	N	N	M
High	MSVESETGSFRDSETR		1	2	1	Glyma.17G215800.1.p	FKBP-ASSOCIATED PROTEIN	1	2378.191671	M	N	N	N	N	N	N	H
Medium	MAEVEEGQVIGVHTVDEWK	1xOxidation [M1]	1	2	1	Glyma.17G254200.1.p	THIOREDOXIN H1	1	1963.919153	M	N	N	N	N	N	N	M
High	YFQGMNEVLAPLFYVFK	1xOxidation [M5]	1	3	1	Glyma.18G001500.1.p	TBC1 DOMAIN FAMILY MEMBER GTPASE-ACTIVATING PROTEIN // SUBFAMILY NOT NAMED	0	3248.492162	N	N	N	N	N	N	H	N
Medium	WNFSIETDSEVVEKGVSR		1	1	1	Glyma.18G009900.1.p	mitotic spindle assembly checkpoint protein MAD2 (MAD2)	1	2293.10299	N	N	N	N	N	M	N	N
High	VFGSPNMVALAQEK		1	1	2	Glyma.18G014800.1.p	HELICASE , IBR AND ZINC FINGER PROTEIN DOMAIN-CONTAINING PROTEIN	0	1826.801389	N	N	N	N	N	H	N	N
Medium	AEIDTSPPFGSVKEAVTRFGGSG PWIPYFNNIENFDLK		1	1	1	Glyma.18G021300.1.p	Weak chloroplast movement under blue light (WEMBL)	2	2244.105239	N	N	N	M	N	N	N	N
High	MVLSKTASESDVSVHSTFASR		1	1	1	Glyma.18G043600.1.p	GROUP II PYRIDOXAL-5-PHOSPHATE DECARBOXYLASE // SUBFAMILY NOT NAMED	1	1925.881036	M	H	N	N	N	N	N	N
Medium	GFAFKGMDVSQRIFTSSR	1xOxidation [M7]	1	1	3	Glyma.18G074700.1.p	TRANSMEMBRANE PROTEIN	2	2239.135336	N	N	N	M	N	N	N	N
Medium	MDSPQSVVSPFRSSVLGESEK	1xOxidation [M1]	1	1	1	Glyma.18G081900.3.p	C2 domain (C2)	2	1602.779197	N	N	N	N	N	N	N	H

Confidence	Sequence	Modifications	Protein Groups	Proteins	PSMs	Master Protein Accessions	Description	Missed Cleavages	Theo. MH+ [Da]	atpF_01	atpF_02	ndhB_01	ndhB_02	rps14_01	rps14_02	Control_01	Control_02
Medium	LTWVQCWGIPIMTWDMKQTHK	1xCarbamidomethyl [C6]; 2xOxidation [M12; M16]	1	1	1	Glyma.18G091400.1.p	SERINE/ARGININE-RICH SPLICING FACTOR 2	0	2968.425312	N	N	N	N	N	M	N	N
High	IQGQPCHRIGSMLPMPR	1xCarbamidomethyl [C6]; 2xOxidation [M12; M15]	1	1	20	Glyma.18G102300.1.p	Helitron helicase-like domain at N-terminus (Helitron_like_N)	1	3476.722222	N	N	N	N	N	M	N	N
Medium	AMATGQGIRDLCNCLR	2xCarbamidomethyl [C12; C14]; 1xOxidation [M2]	1	4	1	Glyma.18G113200.1.p	no GO	2	1373.738318	N	N	N	N	N	N	N	M
Medium	LCWSSFKTISKQSHAFLHR	1xCarbamidomethyl [C2]	1	2	1	Glyma.18G119400.1.p	CAAX AMINO TERMINAL PROTEASE FAMILY PROTEIN	2	3264.536512	N	N	N	N	M	N	N	N
High	ISEKGFIMLEGVWLADMQR	1xOxidation [M17]	1	1	8	Glyma.18G124400.1.p	Endonuclease/Exonuclease/phosphatase family (Exo_endo_phos)	2	1600.847551	N	N	N	N	N	H	N	N
Medium	EKIEDIEHVQR		1	1	2	Glyma.18G134000.1.p	CYTOCHROME P450 89A2-RELATED	2	1616.842466	N	N	H	N	N	N	N	N
High	MSLCEKKPWSSLLCNEIIK	2xCarbamidomethyl [C4; C14]; 1xAcetyl [N-Term]	1	1	3	Glyma.18G162600.1.p	F-box domain (F-box)	0	1374.675584	N	N	N	N	N	N	N	H
Medium	YCENVFGLNRENGHVR	1xCarbamidomethyl [C2]	1	4	2	Glyma.18G174200.4.p	SERINE-THREONINE PROTEIN KINASE // SUBFAMILY NOT NAMED	1	3371.595798	N	N	N	N	N	N	N	M
High	ENLQTCAAIHGESFNAAMSSMHPSAFAQLK	1xCarbamidomethyl [C6]	1	3	1	Glyma.18G203900.1.p	IMPORTIN-11	1	1465.677374	N	N	N	M	N	N	N	N
Medium	FSQINNAYELLSDEEK		0	3	2	Glyma.18G204000	DNAJ DOMAIN-CONTAINING PROTEIN	0	2526.12755	N	M	N	N	N	N	N	N
Medium	MASNQEITAPLRYWVDNER		1	1	1	Glyma.18G226600.1.p	PTHR33103:SF4 - EMB	1	1858.79796	N	N	N	N	N	M	N	N
High	SSVGDSIYHYMHTADK	1xOxidation [M11]	1	2	1	Glyma.18G232800.5.p	ROP GUANINE NUCLEOTIDE EXCHANGE FACTOR 2-RELATED	2	2266.036427	N	N	M	N	N	N	N	N
Medium	FTCFIRGCHDASKLLQYK	2xCarbamidomethyl [C3; C8]	1	2	1	Glyma.18G247900.1.p	no GO	2	3394.655857	N	N	N	H	N	N	N	N
High	HFSIEEMRAATNNFDK	1xOxidation [M7]	1	1	6	Glyma.18G271000.1.p	Protein tyrosine kinase (Pkinase_Tyr) // Carbohydrate-binding protein of the ER (Malectin_like)	1	2264.137979	N	N	M	N	N	N	N	N
Medium	LEQYRNFENILKSGDAVDK		1	1	1	Glyma.18G284000.1.p	WD40 REPEAT PROTEIN // SUBFAMILY NOT NAMED	1	2201.007375	N	N	N	N	N	H	N	N
Medium	ANPGPISGGLAPIYGAAGKMPDR	1xOxidation [M20]	0	3	1	Glyma.18G284300	sphinganine-1-phosphate aldolase (SGPL1, DPL1)	1	1384.688926	N	N	N	N	N	N	N	M
High	VKEESTQMEEKHK		1	1	3	Glyma.19G018600.1.p	AAA-TYPE ATPASE FAMILY PROTEIN-RELATED	1	1882.814849	M	N	N	N	N	N	N	N

Confidence	Sequence	Modifications	Protein Groups	Proteins	PSMs	Master Protein Accessions	Description	Missed Cleavages	Theo. MH+ [Da]	atpF_01	atpF_02	ndhB_01	ndhB_02	rps14_01	rps14_02	Control_01	Control_02
Medium	ELVQMDPLNTEYHILLSNMYALCGK	1xCarbamidomethyl [C23]; 1xOxidation [M5]	1	1	1	Glyma.19G025700.1.p	PPR repeat (PPR) // Pentatricopeptide repeat domain (PPR_3) // DYW family of nucleic acid deaminases (DYW_deaminase)	0	2392.187825	M	N	N	N	N	N	N	N
Medium	TVGECQSPLCDLPGGVITMHVVVQPPSVEKDK	2xCarbamidomethyl [C5; C10]	1	1	1	Glyma.19G028200.1.p	MEMBRANE-ANCHORED UBIQUITIN-FOLD PROTEIN 1-RELATED	2	2289.157728	N	N	N	N	N	N	N	M
Medium	EKGNKAGGSAVEVK		1	1	2	Glyma.19G044300.1.p	translation initiation factor eIF-2B subunit delta (EIF2B4)	2	4108.153575	M	N	N	N	N	N	N	N
Medium	MASTVEVQSSYSAKKQDDSDFNLTEWAVK		1	1	1	Glyma.19G045400.1.p	no GO	1	2212.114073	N	N	N	N	N	N	N	M
High	ARKDNTQEGMLLPK		1	1	1	Glyma.19G068800.1.p	lysine-specific demethylase 3 [EC:1.14.11.-] (KDM3)	2	1866.832506	N	N	N	N	M	N	N	N
High	ARKDNTQEGMLLPK	1xOxidation [M10]	1	1	1	Glyma.19G068800.1.p	lysine-specific demethylase 3 [EC:1.14.11.-] (KDM3)	2	1866.832506	N	N	N	N	M	N	N	N
High	MPDDAICQAILK	1xCarbamidomethyl [C7]	1	6	1	Glyma.19G091200.1.p	SUA5 // SUBFAMILY NOT NAMED	2	2241.110578	N	N	N	N	M	N	N	N
Medium	VPIVIFLHGGGFCFGSRTWPHIHNCCMR	3xCarbamidomethyl [C13; C25; C26]; 1xOxidation [M27]	1	1	1	Glyma.19G202900.1.p	CARBOXYLESTERASE 15-RELATED	2	1853.979611	N	N	N	N	N	N	N	M
Medium	AELSDMKNMNNAK		1	1	1	Glyma.19G204100.1.p	Homogentisate solanesyltransferase / HST	1	2225.156072	N	N	N	N	N	N	N	M
Medium	TNLNFLPHAMEEQTYVEDSCK	1xCarbamidomethyl [C20]	1	2	1	Glyma.19G204200.1.p	CLEAVAGE AND POLYADENYLATION SPECIFICITY FACTOR (CPSF) A SUBUNIT PROTEIN	2	3050.670703	N	N	N	N	N	N	N	M
Medium	TSSSVCGMKGCDEEAIK	2xCarbamidomethyl [C6; C11]	1	1	1	Glyma.19G214100.1.p	CELLULOSE SYNTHASE-LIKE PROTEIN D5-RELATED	0	1901.855198	N	N	N	N	N	N	N	M
Medium	STATSSGSEGGGDPQMIDERKR		1	1	2	Glyma.19G216200.1.p	CAMP-RESPONSE ELEMENT BINDING PROTEIN-RELATED // SUBFAMILY NOT NAMED	1	2170.000225	N	N	N	N	M	N	N	N
High	GSGPLNGFMSGPLERFASGPLDKGGGFMSGPIEK		1	3	1	Glyma.19G222700.1.p	PROTEIN PHOSPHATASE 2C 32	0	1780.787178	N	N	N	N	N	M	N	N
Medium	TDDKQNCAFGINDILLLWK	1xCarbamidomethyl [C7]	1	1	1	Glyma.19G223200.1.p	TYROSINE SPECIFIC PROTEIN PHOSPHATASE AND DUAL SPECIFICITY PROTEIN PHOSPHATASE	1	1465.695133	N	N	N	N	N	N	N	M
High	NNAHSRDTLISNDDIPCMK	1xCarbamidomethyl [C17]	1	1	1	Glyma.19G229200.1.p	F1O19.10 PROTEIN	0	2309.064022	H	H	H	H	N	N	N	H

Confidence	Sequence	Modifications	Protein Groups	Proteins	PSMs	Master Protein Accessions	Description	Missed Cleavages	Theo. MH+ [Da]	atpF_01	atpF_02	ndhB_01	ndhB_02	rps14_01	rps14_02	Control_01	Control_02
Medium	NSNLYKCTSAVK	1xCarbamidomethyl [C7]	1	1	1	Glyma.19G229200.1.p	F1O19.10 PROTEIN	0	2054.124043	N	N	H	H	N	N	H	H
Medium	TEKSTESEAAATDDGNEK		1	1	1	Glyma.19G246100.1.p	Alba (Alba)	2	3023.648387	N	N	H	N	N	N	N	N
Medium	FGSTPSAPTSLLVSSSGSEPLEN K		1	1	1	Glyma.20G070600.1.p	no GO	0	1425.696847	H	H	H	H	N	N	N	H
Medium	DGMKMGKSLGNILEPNDLVNK	1xOxidation [M]	1	1	2	Glyma.20G099600.1.p	METHIONYL-TRNA SYNTHETASE	1	1741.890796	N	N	N	N	N	N	N	H
Medium	KIMGTLTSNINLSPQYNVPKDY PPHGSVVEEIQGLIR		1	1	1	Glyma.20G113800.1.p	CARBOXYLESTERASE 6-RELATED	0	2738.331018	H	N	N	N	N	N	N	N
Medium	VRFMASGASPLSPDIMEFLK	1xOxidation [M4]	1	2	1	Glyma.20G143900.1.p	PROTEIN ACS-13, ISOFORM C	1	2465.165132	N	N	N	N	N	N	N	H
Medium	FGANGGDEGEGSDGSARRK		1	1	1	Glyma.20G161800.2.p	KARYOGAMY PROTEIN KAR4-RELATED	1	2123.094048	N	N	M	H	N	N	N	M
Medium	KFGANGGDEGEGSDGSARR		1	1	1	Glyma.20G161800.2.p	KARYOGAMY PROTEIN KAR4-RELATED	0	1278.585523	H	N	N	N	N	N	H	H
Medium	SKDDSSALSSAARYLSGVSPSR		1	1	1	Glyma.20G185300.1.p	PROTEIN CHUP1, CHLOROPLASTIC	0	900.424378	M	N	H	N	N	N	N	N
Medium	TEKEWKQLFIEAGFK		1	1	1	Glyma.20G213700.1.p	Isoflavone 7-O-methyltransferase	2	2251.189011	N	N	N	M	N	N	M	N
Medium	GGQSVTFVGTSLVIFGGEDAKR		1	1	1	Glyma.20G235500.1.p	KELCH REPEAT DOMAIN // SUBFAMILY NOT NAMED	0	1892.94561	N	N	M	N	N	N	N	N
Medium	MNVSLPFHVTPLSTTNPTELKIR AITK	1xAcetyl [N-Term]	1	1	1	Glyma.20G236600.1.p	TRANSLATIONALLY CONTROLLED TUMOR PROTEIN-RELATED	0	1361.690699	H	H	H	H	H	N	H	H
Medium	HGSFYVDIPEPDPDDAK		1	1	1	Glyma.U005400.1.p	SEC14 RELATED PROTEIN // SUBFAMILY NOT NAMED	1	2104.051666	N	N	H	H	N	N	N	N
Medium	CLGSTDAAVAEIMTQKEDSK	1xCarbamidomethyl [C1]; 1xOxidation [M13]	1	1	1	Glyma.U018000.1.p	MADS BOX PROTEIN // SUBFAMILY NOT NAMED	0	1220.600487	M	M	H	H	N	N	N	H
Medium	FDGQPEVDEETTASQK		1	1	1	Glyma.U023700.2.p	IRON-SULFUR CLUSTER ASSEMBLY PROTEIN // SUBFAMILY NOT NAMED	1	1433.723062	N	N	H	N	N	N	N	N
Medium	VKMTNDSNIANDK	1xOxidation [M3]	1	1	1	Glyma.U028100.1.p	MYB DOMAIN PROTEIN 100-RELATED	1	1036.532289	N	N	M	M	N	N	N	M
High	NAHSATTWSGQYVGGAEAR		1	1	45 4	Streptavidin		1	1422.722334	N	N	N	N	N	N	N	M
High	INTQWLLTSGTTEANAWK		1	1	47	Streptavidin		1	1851.847628	N	N	N	N	N	N	N	M
High	YDSAPATDGSGTALGWTVAW K		1	1	23 7	Streptavidin		1	1689.782702	N	N	N	N	N	N	N	M
High	INTQWLLTSGTTEANAWKSTLV GHDTFTK		1	1	1	Streptavidin		0	1810.808976	N	N	N	N	N	N	N	M
High	STLVGHDTFTK		1	1	23	Streptavidin		1	1851.846759	N	N	N	N	N	N	N	M

*N: Not Found, M: Medium and H: High.
