# Supplementary material for: Identification of soybean trans-factors associated with plastid RNA editing sites
Source: Genet Mol Biol. 2020 May 11;43(1 Suppl 2):e20190067. doi: 10.1590/1678-4685-GMB-2019-0067 (PMC7231544; doi:10.1590/1678-4685-GMB-2019-0067)
Supplement: Table S3 [file 1415-4757-gmb-43-1-s2-e20190067-suppl5.pdf]

# Supplementary Material to "Identification of soybean *trans*-factors associated with plastid RNA editing sites"

**Table S3 -** List of PPR protein sequences used in the phylogenetic analysis.

| Accession | Subclass | Name | Edited gene | Reference |
|---|---|---|---|---|
| AT1G08070 | DYW | OTP82 | ndhG, ndhB | Hammani *et al*. (2009) |
| AT1G11290 | DYW | CRR22 | ndhB, ndhD, rpoB | Okuda *et al*. (2009) |
| AT1G15510 | DYW | AtECB2/VAC1 | accD | Yu *et al*. (2009), Tseng *et al*. (2010) |
| AT1G59720 | DYW | CRR28 | ndhB, ndhD | Okuda *et al*. (2009) |
| AT2G02980 | DYW | OTP85 | ndhD | Hammani *et al*. (2009) |
| AT2G29760 | DYW | OTP81 | rps12 intron | Hammani *et al*. (2009) |
| AT3G22690 | DYW | YS1 | rpoB | Zhou *et al*. (2008) |
| AT3G57430 | DYW | OTP84 | ndhF, psbZ, ndhB | Hammani *et al*. (2009) |
| AT3G63370 | DYW | OTP86 | rps14 | Hammani *et al*. (2009) |
| AT5G13270 | DYW | RARE1 | accD | Robbins *et al*. (2009) |
| AT5G48910 | DYW | LPA66 | psbF | Cai *et al*. (2009) |
| AT2G45350 | E | CRR4 | ndhD | Kotera *et al*. (2005) |
| AT5G59200 | E | OTP80 | rpl23 | Hammani *et al*. (2009) |

| **Accession** | **Subclass** | **Name** | **Edited gene** | **Reference** |
|---|---|---|---|---|
| AT1G05750 | E | CLB19 | rpoA, clpP | Chateigner-Boutin *et al.* (2008) |
| AT5G55740 | E | CRR21 | ndhD | Okuda *et al*. (2007) |
| AT3G22150 | E | AEF1/MPR25 | atpF | Yap *et al.* (2015) |
| AT1G12300 | - | - | - | - |
| AT1G12620 | - | - | - | - |
| AT1G12775 | - | - | - | - |
| AT1G68930 | - | - | - | - |
| AT1G74630 | - | - | - | - |
| AT3G13770 | - | - | - | - |
| AT3G22470 | - | - | - | - |
| AT3G46790 | - | - | - | - |
| AT3G49170 | - | - | - | - |
| AT3G62470 | - | - | - | - |
| AT5G04780 | - | - | - | - |
| AT5G15340 | - | - | - | - |
| AT5G61990 | - | - | - | - |
| AT5G39710 | - | - | - | - |

| **Accession** | **Subclass** | **Name** | **Edited gene** | **Reference** |
|---|---|---|---|---|
| AT5G50280 | - | - | - | - |
| AT5G59900 | - | - | - | - |
| Glyma.01G016100 | - | - | - | - |
| Glyma.02G174500 | - | - | - | - |
| Glyma.11G111200 | - | - | - | - |
| Glyma.11G217500 | - | - | - | - |
| Glyma.19G025700 | - | - | - | - |
