# Supplementary material for: Identification of soybean trans-factors associated with plastid RNA editing sites
Source: Genet Mol Biol. 2020 May 11;43(1 Suppl 2):e20190067. doi: 10.1590/1678-4685-GMB-2019-0067 (PMC7231544; doi:10.1590/1678-4685-GMB-2019-0067)
Supplement: Material S1 [file 1415-4757-gmb-43-1-s2-e20190067-suppl6.pdf]

Genetics and Molecular Biology 43, 1(suppl 2) (2020)

Supplementary Material to "Identification of soybean *trans*-factors associated with plastid RNA editing sites"

Material S1 - Individual PPR-probe alignments of each RNA probe and their corresponding *p*-values.

Protein: Glyma.01G016100
Motif Locations:
[(97, 127), (152, 186), (187, 221), (222, 260), (261, 295), (296, 330), (331, 365), (366, 400), (401, 435), (436, 470), (471, 505), (506, 540), (541, 575), (576, 610), (611, 645), (646, 680), (681, 715)]
['P', 'P', 'P', 'P', 'P', 'P', 'P', 'P', 'P', 'P', 'P', 'P', 'P', 'P']
Motif Binding Pairs:
['S:RP', 'D:DP', 'N:NP', 'T:TP', 'N:SP', 'D:NP', 'S:SP', 'D:TP', 'T:EP', 'D:RP', 'N:NP', 'S:NP', 'N:SP', 'D:TP']
Bining Protein to Target: ***ndhB*-1481**
Alignment Starts at: 9 on transcript.
Alignment: 1
1'________SDNTNDSDTDNSND______________
6 ________RDNTSNSTERNNST______________
T AUUGUAUGUGUGAUAGCAUCUACUAUACCAGGAAUA
Has Score: -7.4390007654505315
Raw: -136.12541424185483
p-value: 0.0011397109288538443
Alignment Starts at: 18 on transcript.
Alignment: 2
1'_________________SDNTNDSDTDNSND_____
6 _________________RDNTSNSTERNNST_____
T AUUGUAUGUGUGAUAGCAUCUACUAUACCAGGAAUA
Has Score: -7.576667812267827
Raw: -137.91508585047973
p-value: 0.007303724489092408
Alignment Starts at: 19 on transcript.
Alignment: 3
1'__________________SDNTNDSDTDNSND____
6 __________________RDNTSNSTERNNST____
T AUUGUAUGUGUGAUAGCAUCUACUAUACCAGGAAUA
Has Score: -7.87569655160574
Raw: -141.8024594618726
p-value: 0.13166110953976728
Alignment Starts at: 3 on transcript.
Alignment: 4
1'__SDNTNDSDTDNSND____________________
6 __RDNTSNSTERNNST____________________
T AUUGUAUGUGUGAUAGCAUCUACUAUACCAGGAAUA
Has Score: -7.9022097339334945
Raw: -142.14713083213334
p-value: 0.15835772075952692
Alignment Starts at: 1 on transcript.
Alignment: 5
1'SDNTNDSDTDNSND______________________
6 RDNTSNSTERNNST______________________
T AUUGUAUGUGUGAUAGCAUCUACUAUACCAGGAAUA
Has Score: -7.967386492424817
Raw: -142.9944286925206
p-value: 0.23799504749504208
Alignment Starts at: 21 on transcript.
Alignment: 6
1'____________________SDNTNDSDTDNSND__
6 ____________________RDNTSNSTERNNST__
T AUUGUAUGUGUGAUAGCAUCUACUAUACCAGGAAUA
Has Score: -7.986660711562875
Raw: -143.24499354131535
p-value: 0.26517849387911085
Alignment Starts at: 12 on transcript.
Alignment: 7
1'___________SDNTNDSDTDNSND___________
6 ___________RDNTSNSTERNNST___________
T AUUGUAUGUGUGAUAGCAUCUACUAUACCAGGAAUA
Has Score: -8.014986684271053
Raw: -143.6132311865217

p-value: 0.30780099583767007
##

Protein: Glyma.02G174500
Motif Locations:
[(56, 90), (91, 121), (122, 156), (157, 191), (192, 222), (223, 257), (258, 292), (293, 323), (324, 358), (360, 394), (397, 427), (429, 459), (463, 497), (507, 544), (545, 614), (615, 633)]
['L', 'S', 'P', 'L', 'S', 'P', 'L', 'S', 'P', 'L', 'S', 'S', 'P']
Motif Binding Pairs:
['C:NL', 'N:TS', 'N:TP', 'H:AL', 'D:SS', 'N:TP', 'Y:TL', 'T:NS', 'D:NP', 'D:LL', 'S:GS', 'N:GS', 'D:VP']
Bining Protein to Target: ***rps14*-80**
Alignment Starts at: 6 on transcript.
Alignment: 1
1'_____CNNHDNYTDDSND__________________
6 _____NTTASTTNNLGGV__________________
T CAGAAAUAUCAUUUGAUUCGCCGAUCCUCAAAAAAA
Has Score: -8.079642965144444
Raw: -137.84918310362812
p-value: 0.018992400251398017
Alignment Starts at: 3 on transcript.
Alignment: 2
1'__CNNHDNYTDDSND_____________________
6 __NTTASTTNNLGGV_____________________
T CAGAAAUAUCAUUUGAUUCGCCGAUCCUCAAAAAAA
Has Score: -8.137292693599617
Raw: -138.5409798450902
p-value: 0.03429001031602907
Alignment Starts at: 1 on transcript.
Alignment: 3
1'CNNHDNYTDDSND_______________________
6 NTTASTTNNLGGV_______________________
T CAGAAAUAUCAUUUGAUUCGCCGAUCCUCAAAAAAA
Has Score: -8.257204201483395
Raw: -139.9799179396955
p-value: 0.09797602563101043
Alignment Starts at: 19 on transcript.
Alignment: 4
1'__________________CNNHDNYTDDSND_____
6 __________________NTTASTTNNLGGV_____
T CAGAAAUAUCAUUUGAUUCGCCGAUCCUCAAAAAAA
Has Score: -8.308652620621197
Raw: -140.5972989693491
p-value: 0.14307098324549716
Alignment Starts at: 11 on transcript.
Alignment: 5
1'__________CNNHDNYTDDSND_____________
6 __________NTTASTTNNLGGV_____________
T CAGAAAUAUCAUUUGAUUCGCCGAUCCUCAAAAAAA
Has Score: -8.364305068335836
Raw: -141.2651283419248
p-value: 0.20566321347488992
Alignment Starts at: 14 on transcript.
Alignment: 6
1'_____________CNNHDNYTDDSND__________
6 _____________NTTASTTNNLGGV__________
T CAGAAAUAUCAUUUGAUUCGCCGAUCCUCAAAAAAA
Has Score: -8.379260775653465
Raw: -141.44459682973633
p-value: 0.22491308006242094
Alignment Starts at: 4 on transcript.
Alignment: 7
1'___CNNHDNYTDDSND____________________
6 ___NTTASTTNNLGGV____________________
T CAGAAAUAUCAUUUGAUUCGCCGAUCCUCAAAAAAA
Has Score: -8.440306249594746
Raw: -142.1771425170317
p-value: 0.3131671739570225
##

Protein: Glyma.11G111200
Motif Locations:
[(223, 253), (258, 292), (293, 328), (329, 363), (364, 398), (399, 433), (434, 469), (470, 504), (505, 539), (540, 574), (575, 609), (610, 644)]
['P', 'P', 'P', 'P', 'P', 'P', 'P', 'P']
Motif Binding Pairs:
['D:TP', 'D:NP', 'G:SP', 'N:GP', 'T:NP', 'N:NP', 'T:TP', 'S:TP']
Bining Protein to Target: ***ndhB*-1481**
Alignment Starts at: 4 on transcript.
Alignment: 1
1'___DDGNTNTS_________________________
6 ___TNSGNNTT_________________________
T AUUGUAUGUGUGAUAGCAUCUACUAUACCAGGAAUA
Has Score: -7.919077696097026
Raw: -103.65143991628977
p-value: 0.022154836480497382
Alignment Starts at: 10 on transcript.
Alignment: 2
1'_________DDGNTNTS___________________
6 _________TNSGNNTT___________________
T AUUGUAUGUGUGAUAGCAUCUACUAUACCAGGAAUA
Has Score: -8.020348487172466
Raw: -104.36033545381791
p-value: 0.04656647318835122
Alignment Starts at: 16 on transcript.
Alignment: 3
1'_______________DDGNTNTS_____________
6 _______________TNSGNNTT_____________
T AUUGUAUGUGUGAUAGCAUCUACUAUACCAGGAAUA
Has Score: -8.057278586479823
Raw: -104.61884614896944
p-value: 0.059615678751222985
Alignment Starts at: 25 on transcript.
Alignment: 4
1'________________________DDGNTNTS____
6 ________________________TNSGNNTT____
T AUUGUAUGUGUGAUAGCAUCUACUAUACCAGGAAUA
Has Score: -8.119369512966744
Raw: -105.05348263437794
p-value: 0.08780181546344817
Alignment Starts at: 6 on transcript.
Alignment: 5
1'_____DDGNTNTS_______________________
6 _____TNSGNNTT_______________________
T AUUGUAUGUGUGAUAGCAUCUACUAUACCAGGAAUA
Has Score: -8.216140773479859
Raw: -105.73088145796963
p-value: 0.14984326987114926
Alignment Starts at: 18 on transcript.
Alignment: 6
1'_________________DDGNTNTS___________
6 _________________TNSGNNTT___________
T AUUGUAUGUGUGAUAGCAUCUACUAUACCAGGAAUA
Has Score: -8.260245842286945
Raw: -106.03961693961931
p-value: 0.1860664977408389
Alignment Starts at: 2 on transcript.
Alignment: 7
1'_DDGNTNTS___________________________
6 _TNSGNNTT___________________________
T AUUGUAUGUGUGAUAGCAUCUACUAUACCAGGAAUA
Has Score: -8.274064360352453
Raw: -106.13634656607776
p-value: 0.19844909647285602
###
Bining Protein to Target: ***rps14*-80**
Alignment Starts at: 17 on transcript.
Alignment: 1
1'________________DDGNTNTS____________
6 ________________TNSGNNTT____________

T CAGAAAUAUCAUUUGAUUCGCCGAUCCUCAAAAAAA
Has Score: -7.905259178031517
Raw: -103.5547102898313
p-value: 0.01986921332929131
Alignment Starts at: 24 on transcript.
Alignment: 2
1'______________________DDGNTNTS______
6 ______________________TNSGNNTT______
T CAGAAAUAUCAUUUGAUUCGCCGAUCCUCAAAAAAA
Has Score: -7.9463566169532
Raw: -103.84239236228315
p-value: 0.02732132879760668
Alignment Starts at: 23 on transcript.
Alignment: 3
1'________DDGNTNTS____________________
6 ________TNSGNNTT____________________
T CAGAAAUAUCAUUUGAUUCGCCGAUCCUCAAAAAAA
Has Score: -8.005186713110122
Raw: -104.25420303538156
p-value: 0.04192140678744788
Alignment Starts at: 9 on transcript.
Alignment: 4
1'_______DDGNTNTS_____________________
6 _______TNSGNNTT_____________________
T CAGAAAUAUCAUUUGAUUCGCCGAUCCUCAAAAAAA
Has Score: -8.01618114755814
Raw: -104.3311640765176
p-value: 0.045250368326222416
Alignment Starts at: 8 on transcript.
Alignment: 5
1'_____DDGNTNTS_______________________
6 _____TNSGNNTT_______________________
T CAGAAAUAUCAUUUGAUUCGCCGAUCCUCAAAAAAA
Has Score: -8.298335651323224
Raw: -106.3062456028732
p-value: 0.22136606232716682
Alignment Starts at: 6 on transcript.
Alignment: 6
1'_____________DDGNTNTS_______________
6 _____________TNSGNNTT_______________
T CAGAAAUAUCAUUUGAUUCGCCGAUCCUCAAAAAAA
Has Score: -8.3174115645553
Raw: -106.4397769954977
p-value: 0.24039351811497128
Alignment Starts at: 14 on transcript.
Alignment: 7
1'_____________DDGNTNTS_______________
6 _____________TNSGNNTT_______________
T CAGAAAUAUCAUUUGAUUCGCCGAUCCUCAAAAAAA
Has Score: -8.322083568329273
Raw: -106.47248102191558
p-value: 0.24518589116474898
##

Protein: Glyma.11G217500
Motif Locations:
[(103, 133), (137, 171), (178, 213), (215, 250), (251, 285), (287, 321), (322, 356), (357, 387), (392, 426), (427, 457)]
['S', 'P', 'P', 'P', 'P', 'L']
Motif Binding Pairs:
['S:NS', 'D:AP', 'N:AP', 'G:DP', 'T:KP', 'D:NL']
Bining Protein to Target: ***atpF*-92**
Alignment Starts at: 9 on transcript.
Alignment: 1
1'________SDNGTD______________________
6 ________NAADKN______________________
T UUUAAUACCGAUAUUUUAGCAACAAAUCCAAUAAAU
Has Score: -9.609513573728524
Raw: -99.17591640243802
p-value: 0.030724466803667392

Alignment Starts at: 2 on transcript.
Alignment: 2
1'_SDNGTD_____________________________
6 _NAADKN_____________________________
T UUUAAUACCGAUAUUUUAGCAACAAAUCCAAUAAAU
Has Score: -9.654142283991366
Raw: -99.39905995375221
p-value: 0.05552578088904154
Alignment Starts at: 16 on transcript.
Alignment: 3
1'_______________SDNGTD_______________
6 _______________NAADKN_______________
T UUUAAUACCGAUAUUUUAGCAACAAAUCCAAUAAAU
Has Score: -9.654142283991366
Raw: -99.39905995375229
p-value: 0.05552578088904154
Alignment Starts at: 18 on transcript.
Alignment: 4
1'_________________SDNGTD_____________
6 _________________NAADKN_____________
T UUUAAUACCGAUAUUUUAGCAACAAAUCCAAUAAAU
Has Score: -9.711678698481723
Raw: -99.68674202620407
p-value: 0.10810610836836121
Alignment Starts at: 5 on transcript.
Alignment: 5
1'____SDNGTD__________________________
6 ____NAADKN__________________________
T UUUAAUACCGAUAUUUUAGCAACAAAUCCAAUAAAU
Has Score: -9.792771720103357
Raw: -100.09220713431216
p-value: 0.2315531459756599
Alignment Starts at: 3 on transcript.
Alignment: 6
1'__SDNGTD____________________________
6 __NAADKN____________________________
T UUUAAUACCGAUAUUUUAGCAACAAAUCCAAUAAAU
Has Score: -9.792771720103357
Raw: -100.09220713431216
p-value: 0.2315531459756599
Alignment Starts at: 6 on transcript.
Alignment: 7
1'_____SDNGTD_________________________
6 _____NAADKN_________________________
T UUUAAUACCGAUAUUUUAGCAACAAAUCCAAUAAAU
Has Score: -9.792771720103357
Raw: -100.09220713431216
p-value: 0.2315531459756599
##

Protein: Glyma.19G025700
Motif Locations:
[(10, 44), (47, 77), (86, 110), (111, 145), (146, 180), (177, 207), (208, 242), (243, 322), (323, 358), (359, 393), (390, 420), (424, 459), (460, 494), (495, 522), (526, 560)]
['P', 'L', 'S', 'P', 'S', 'S', 'P', 'P', 'L', 'S', 'S']
Motif Binding Pairs:
['S:RP', 'A:NL', 'D:SS', 'H:IP', 'S:NS', 'N:TS', 'L:TP', 'N:RP', 'G:CL', 'N:TS', 'D:NS']
Bining Protein to Target: ***atpF*-92**
Alignment Starts at: 17 on transcript.
Alignment: 1
1'________________SADHSNLNGND_________
6 ________________RNSINTTRCTN_________
T UUUAAUACCGAUAUUUUAGCAACAAAUCCAAUAAAU
Has Score: -8.167041294917103
Raw: -125.50301190519482
p-value: 0.0019887637475321067
Alignment Starts at: 2 on transcript.
Alignment: 2
1'_SADHSNLNGND________________________

6 _RNSINTTRCTN________________________
T UUUAAUACCGAUAUUUUAGCAACAAAUCCAAUAAAU
Has Score: -8.29231759176664
Raw: -126.75577487369026
p-value: 0.015237171078322643
Alignment Starts at: 13 on transcript.
Alignment: 3
1'____________SADHSNLNGND_____________
6 ____________RNSINTTRCTN_____________
T UUUAAUACCGAUAUUUUAGCAACAAAUCCAAUAAAU
Has Score: -8.409078107782147
Raw: -127.92338003384529
p-value: 0.06726986533431965
Alignment Starts at: 8 on transcript.
Alignment: 4
1'_______SADHSNLNGND__________________
6 _______RNSINTTRCTN__________________
T UUUAAUACCGAUAUUUUAGCAACAAAUCCAAUAAAU
Has Score: -8.45503134071999
Raw: -128.38291236322374
p-value: 0.10864816957467538
Alignment Starts at: 25 on transcript.
Alignment: 5
1'________________________SADHSNLNGND_
6 ________________________RNSINTTRCTN_
T UUUAAUACCGAUAUUUUAGCAACAAAUCCAAUAAAU
Has Score: -8.51204004950026
Raw: -128.9529994510264
p-value: 0.1819763672220555
Alignment Starts at: 16 on transcript.
Alignment: 6
1'_______________SADHSNLNGND__________
6 _______________RNSINTTRCTN__________
T UUUAAUACCGAUAUUUUAGCAACAAAUCCAAUAAAU
Has Score: -8.51204004950026
Raw: -128.95299945102641
p-value: 0.1819763672220555
Alignment Starts at: 22 on transcript.
Alignment: 7
1'_____________________SADHSNLNGND____
6 _____________________RNSINTTRCTN____
T UUUAAUACCGAUAUUUUAGCAACAAAUCCAAUAAAU
Has Score: -8.517446771627288
Raw: -129.0070666722967
p-value: 0.19025656445719247
##
