# Supplementary material for: Identification of soybean trans-factors associated with plastid RNA editing sites
Source: Genet Mol Biol. 2020 May 11;43(1 Suppl 2):e20190067. doi: 10.1590/1678-4685-GMB-2019-0067 (PMC7231544; doi:10.1590/1678-4685-GMB-2019-0067)
Supplement: Material S2 [file 1415-4757-gmb-43-1-s2-e20190067-suppl7.pdf]

Genetics and Molecular Biology 43, 1(suppl 2) (2020)

Supplementary Material to "Identification of soybean *trans*-factors associated with plastid RNA editing sites"

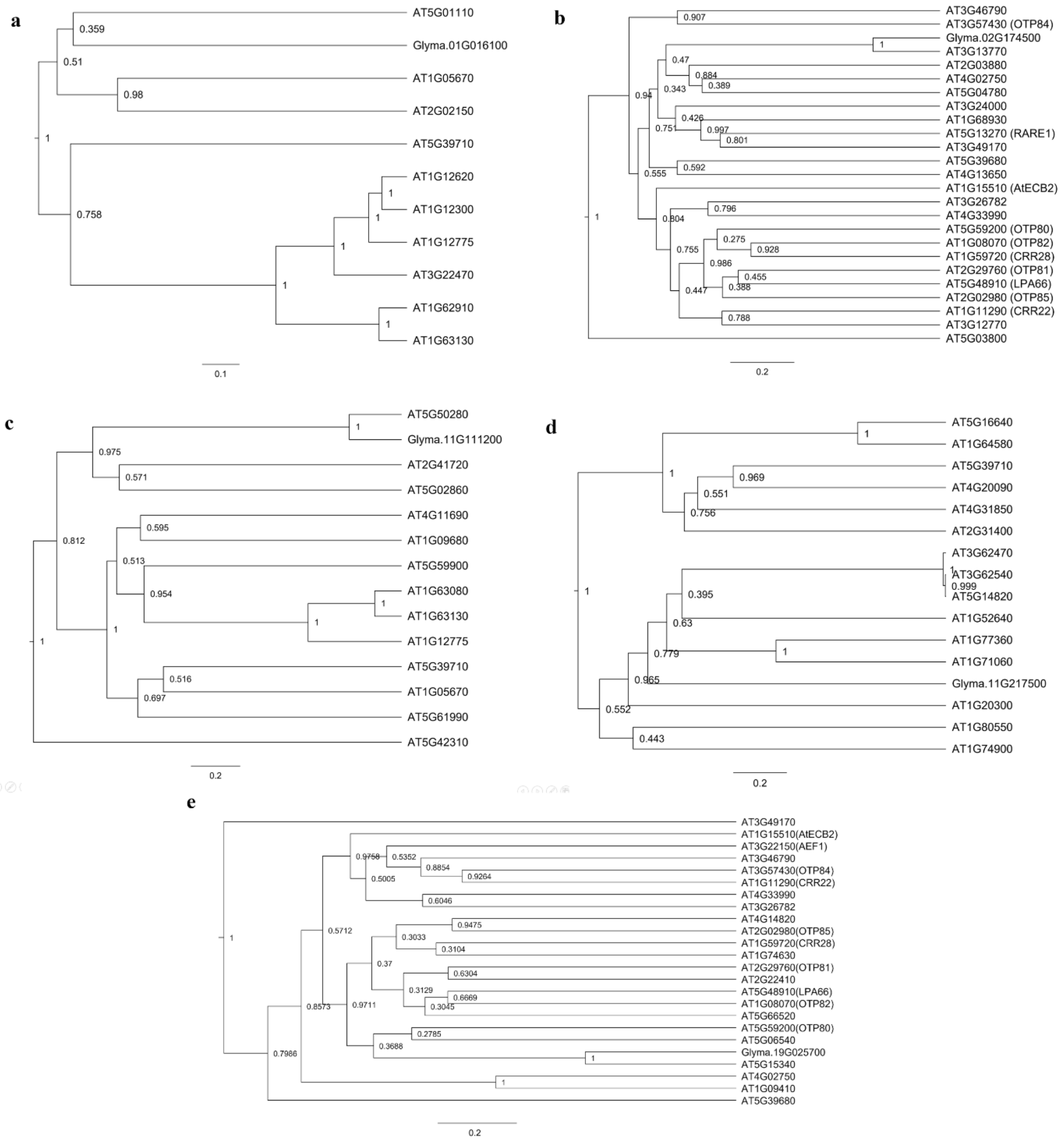

Material S2 - Phylogenetic tree of the PPR protein (a) Glyma.01G016100, (b) Glyma.02G174500, (c) Glyma.11G111200, (d) Glyma.11G217500, and (e) Glyma.19g025700. The phylogenetic analysis was performed with PPR protein sequences from *A. thaliana* and *G. max*. Posteriori probabilities are labeled above the branches.
