# Supplementary material for: Identification of soybean trans-factors associated with plastid RNA editing sites
Source: Genet Mol Biol. 2020 May 11;43(1 Suppl 2):e20190067. doi: 10.1590/1678-4685-GMB-2019-0067 (PMC7231544; doi:10.1590/1678-4685-GMB-2019-0067)
Supplement: Material S3 [file 1415-4757-gmb-43-1-s2-e20190067-suppl8.pdf]

Supplementary Material to "Identification of soybean *trans*-factors associated with plastid RNA editing sites"

Material S3 - Arabidopsis and soybean PPR-probe alignments and their corresponding *p*-values.

Protein: AEF1/MPR25
Motif Locations:
[(37, 67), (68, 98), (105, 135), (140, 176), (177, 211), (212, 246), (249, 283), (280, 315), (316, 346), (351, 381), (382, 416), (417, 451), (452, 483), (484, 518), (519, 549), (554, 584), (585, 619), (620, 650), (656, 690), (724, 759)]
['S', 'P', 'P', 'P', 'P', 'L', 'S', 'P', 'L', 'S', 'P', 'L', 'S', 'P', 'L', 'S', 'P']
Motif Binding Pairs:
['T:RS', 'D:NP', 'S:SP', 'N:NP', 'S:NP', 'D:VL', 'N:SS', 'D:NP', 'P:LL', 'D:NS', 'D:NP', 'E:TL', 'D:YS', 'N:NP', 'N:AL', 'N:SS', 'D:TP']
Bining Protein to Target: **atpF-92**
Alignment Starts at: 5 on transcript.
Alignment: 1
1'____TDSNSDNDPDDEDNNND_______________
6 ____RNSNNVSNLNNTYNAST_______________
T UUUAAUACCGAUAUUUUAGCAACAAAUCCAAUAAAU
Has Score: -7.18059517763961
Raw: -149.85638475758165
p-value: 0.00333962771967284
Alignment Starts at: 7 on transcript.
Alignment: 2
1'______TDSNSDNDPDDEDNNND_____________
6 ______RNSNNVSNLNNTYNAST_____________
T UUUAAUACCGAUAUUUUAGCAACAAAUCCAAUAAAU
Has Score: -7.4160902814804786
Raw: -153.62430641903552
p-value: 0.06621175631426134
Alignment Starts at: 19 on transcript.
Alignment: 3
1'__________________TDSNSDNDPDDEDNNND_
6 __________________RNSNNVSNLNNTYNAST_
T UUUAAUACCGAUAUUUUAGCAACAAAUCCAAUAAAU
Has Score: -7.423303037798983
Raw: -153.73971052013147
p-value: 0.07110391775149527
Alignment Starts at: 16 on transcript.
Alignment: 4
1'_______________TDSNSDNDPDDEDNNND____
6 _______________RNSNNVSNLNNTYNAST____
T UUUAAUACCGAUAUUUUAGCAACAAAUCCAAUAAAU
Has Score: -7.444726649623014
Raw: -154.08248830931598
p-value: 0.08727387059600217
Alignment Starts at: 3 on transcript.
Alignment: 5
1'__TDSNSDNDPDDEDNNND_________________
6 __RNSNNVSNLNNTYNAST_________________
T UUUAAUACCGAUAUUUUAGCAACAAAUCCAAUAAAU
Has Score: -7.499048375367604
Raw: -154.95163592122958
p-value: 0.14026898101923124
Alignment Starts at: 2 on transcript.

Alignment: 6
1'_TDSNSDNDPDDEDNNND__________________
6 _RNSNNVSNLNNTYNAST__________________
T UUUAAUACCGAUAUUUUAGCAACAAAUCCAAUAAAU
Has Score: -7.550340299678709

Raw: -155.77230671020726
p-value: 0.2072428135764579
Alignment Starts at: 11 on transcript.
Alignment: 7
1'__________TDSNSDNDPDDEDNNND_________
6 __________RNSNNVSNLNNTYNAST_________
T UUUAAUACCGAUAUUUUAGCAACAAAUCCAAUAAAU
Has Score: -7.596637103557807
Raw: -156.51305557227275
p-value: 0.2814436872090682
##

Protein: Glyma.14G003000.1
Motif Locations:
[(23, 53), (54, 88), (90, 120), (124, 158), (159, 193), (194, 227), (228, 258), (259, 293), (295, 325), (330, 360), (361, 395), (396, 430), (431, 462), (463, 497), (498, 528), (533, 563), (564, 598), (599, 629), (635, 669), (689, 724)]
['S', 'P', 'S', 'P', 'L', 'S', 'S', 'P', 'L', 'S', 'P', 'L', 'S', 'P', 'L', 'S', 'P']
Motif Binding Pairs:
['S:RS', 'D:NP', 'S:SS', 'N:NP', 'S:NL', 'D:VS', 'N:SS', 'D:NP', 'P:LL', 'D:NS', 'D:NP', 'E:TL', 'D:YS', 'N:NP', 'N:AL', 'N:TS', 'D:TP']
Bining Protein to Target: atpF-92
Alignment Starts at: 5 on transcript.
Alignment: 1
1'____SDSNSDNDPDDEDNNND_______________
6 ____RNSNNVSNLNNTYNATT_______________
T UUUAAUACCGAUAUUUUAGCAACAAAUCCAAUAAAU
Has Score: -7.47823104856447
Raw: -154.6185586923794
p-value: 0.0320656104586755
Alignment Starts at: 7 on transcript.
Alignment: 2
1'______SDSNSDNDPDDEDNNND_____________
6 ______RNSNNVSNLNNTYNATT_____________
T UUUAAUACCGAUAUUUUAGCAACAAAUCCAAUAAAU
Has Score: -7.508988895210283
Raw: -155.1106842387124
p-value: 0.046192391250036935
Alignment Starts at: 3 on transcript.
Alignment: 3
1'__SDSNSDNDPDDEDNNND_________________
6 __RNSNNVSNLNNTYNATT_________________
T UUUAAUACCGAUAUUUUAGCAACAAAUCCAAUAAAU
Has Score: -7.660610257139165
Raw: -157.53662602957456
p-value: 0.19677238484897036
Alignment Starts at: 15 on transcript.
Alignment: 4
1'______________SDSNSDNDPDDEDNNND_____
6 ______________RNSNNVSNLNNTYNATT_____
T UUUAAUACCGAUAUUUUAGCAACAAAUCCAAUAAAU
Has Score: -7.683490115633952
Raw: -157.90270376549105
p-value: 0.23330842951574787
Alignment Starts at: 10 on transcript.
Alignment: 5
1'_________SDSNSDNDPDDEDNNND__________
6 _________RNSNNVSNLNNTYNATT__________
T UUUAAUACCGAUAUUUUAGCAACAAAUCCAAUAAAU
Has Score: -7.697934598194164
Raw: -158.13381548645447
p-value: 0.2581867036653473
Alignment Starts at: 19 on transcript.
Alignment: 6
1'__________________SDSNSDNDPDDEDNNND_
6 __________________RNSNNVSNLNNTYNATT_
T UUUAAUACCGAUAUUUUAGCAACAAAUCCAAUAAAU
Has Score: -7.7018525556268935

Raw: -158.19650280537806
p-value: 0.26516433675400963
Alignment Starts at: 6 on transcript.
Alignment: 7
1'_____SDSNSDNDPDDEDNNND______________
6 _____RNSNNVSNLNNTYNATT______________
T UUUAAUACCGAUAUUUUAGCAACAAAUCCAAUAAAU
Has Score: -7.714154047911982
Raw: -158.3933266819396
p-value: 0.2876772824434619
##

Protein: Glyma.02G309700.1
Motif Locations:
[(20, 50), (51, 85), (87, 121), (128, 158), (159, 193), (194, 227), (228, 258), (259, 293), (295, 325), (330, 360), (361, 395), (396, 430), (431, 462), (463, 497), (506, 523), (524, 558), (560, 594), (628, 663)]
['S', 'P', 'L', 'S', 'P', 'S', 'S', 'P', 'L', 'S', 'P', 'L', 'S', 'P', 'S']
Motif Binding Pairs:
['S:RS', 'D:NP', 'L:SL', 'N:SS', 'T:NP', 'D:VS', 'N:SS', 'D:NP', 'P:LL', 'D:NS', 'D:NP', 'E:TL', 'D:YS', 'L:NP', 'D:AS']
Bining Protein to Target: atpF-92
Alignment Starts at: 5 on transcript.
Alignment: 1
1'____SDLNTDNDPDDEDLD_________________
6 ____RNSSNVSNLNNTYNA_________________
T UUUAAUACCGAUAUUUUAGCAACAAAUCCAAUAAAU
Has Score: -7.567912349114486
Raw: -143.88999818145993
p-value: 0.0038561872679981585
Alignment Starts at: 2 on transcript.
Alignment: 2
1'_SDLNTDNDPDDEDLD____________________
6 _RNSSNVSNLNNTYNA____________________
T UUUAAUACCGAUAUUUUAGCAACAAAUCCAAUAAAU
Has Score: -7.789347512605787
Raw: -146.99009047033817
p-value: 0.07587702293625857
Alignment Starts at: 8 on transcript.
Alignment: 3
1'_______SDLNTDNDPDDEDLD______________
6 _______RNSSNVSNLNNTYNA______________
T UUUAAUACCGAUAUUUUAGCAACAAAUCCAAUAAAU
Has Score: -7.795914476557621
Raw: -147.08202796566385
p-value: 0.08122884957449844
Alignment Starts at: 7 on transcript.
Alignment: 4
1'______SDLNTDNDPDDEDLD_______________
6 ______RNSSNVSNLNNTYNA_______________
T UUUAAUACCGAUAUUUUAGCAACAAAUCCAAUAAAU
Has Score: -7.8313322743845095
Raw: -147.57787713524027
p-value: 0.11508102005991666
Alignment Starts at: 19 on transcript.
Alignment: 5
1'__________________SDLNTDNDPDDEDLD___
6 __________________RNSSNVSNLNNTYNA___
T UUUAAUACCGAUAUUUUAGCAACAAAUCCAAUAAAU
Has Score: -7.867345295128739
Raw: -148.0820594256595
p-value: 0.1587233256054339
Alignment Starts at: 1 on transcript.
Alignment: 6
1'SDLNTDNDPDDEDLD_____________________
6 RNSSNVSNLNNTYNA_____________________
T UUUAAUACCGAUAUUUUAGCAACAAAUCCAAUAAAU
Has Score: -7.908276638576424
Raw: -148.65509823392708

p-value: 0.22001296669620174
Alignment Starts at: 18 on transcript.
Alignment: 7
1'_________________SDLNTDNDPDDEDLD____
6 _________________RNSSNVSNLNNTYNA____
T UUUAAUACCGAUAUUUUAGCAACAAAUCCAAUAAAU
Has Score: -7.923256676503715
Raw: -148.86481876490913
p-value: 0.24545465466797345
###

Protein: Glyma.06G206900.1
Motif Locations:
[(51, 85), (90, 120), (126, 156), (157, 192), (193, 227), (228, 262), (259, 289), (297, 331), (332, 362), (363, 393), (399, 433), (434, 464), (465, 499), (500, 534), (535, 565), (566, 600), (601, 635), (636, 666), (667, 701), (702, 737), (738, 768), (770, 800), (804, 838), (867, 901), (902, 955), (956, 974)]
['P', 'L', 'S', 'P', 'L', 'S', 'P', 'L', 'S', 'P', 'L', 'S', 'P', 'L', 'S', 'P', 'L', 'S', 'P', 'L', 'S']
Motif Binding Pairs:
['S:LP', 'D:GL', 'D:TS', 'D:NP', 'D:PL', 'N:NS', 'D:NP', 'E:VL', 'N:NS', 'N:NP', 'D:LL', 'T:NS', 'D:NP', 'D:GL', 'S:IS', 'Q:NP', 'D:TL', 'D:CS', 'D:NP', 'K:LL', 'D:AS']
Bining Protein to Target: atpF-92
Alignment Starts at: 9 on transcript.
Alignment: 1
1'________SDDDDNDENNDTDDSQDDDKD_______
6 ________LGTNPNNVNNLNNGINTCNLA_______
T UUUAAUACCGAUAUUUUAGCAACAAAUCCAAUAAAU
Has Score: -7.804801246746557
Raw: -185.04628222865625
p-value: 0.12296767105308098
Alignment Starts at: 3 on transcript.
Alignment: 2
1'__SDDDDNDENNDTDDSQDDDKD_____________
6 __LGTNPNNVNNLNNGINTCNLA_____________
T UUUAAUACCGAUAUUUUAGCAACAAAUCCAAUAAAU
Has Score: -7.8105717393221
Raw: -185.1616920801671
p-value: 0.13217729691489744
Alignment Starts at: 11 on transcript.
Alignment: 3
1'__________SDDDDNDENNDTDDSQDDDKD_____
6 __________LGTNPNNVNNLNNGINTCNLA_____
T UUUAAUACCGAUAUUUUAGCAACAAAUCCAAUAAAU
Has Score: -7.815472461838796
Raw: -185.25970653050106
p-value: 0.14036363472839114
Alignment Starts at: 5 on transcript.
Alignment: 4
1'____SDDDDNDENNDTDDSQDDDKD___________
6 ____LGTNPNNVNNLNNGINTCNLA___________
T UUUAAUACCGAUAUUUUAGCAACAAAUCCAAUAAAU
Has Score: -7.859563176956006
Raw: -186.14152083284523
p-value: 0.2291744504754446
Alignment Starts at: 8 on transcript.
Alignment: 5
1'_______SDDDDNDENNDTDDSQDDDKD________
6 _______LGTNPNNVNNLNNGINTCNLA________
T UUUAAUACCGAUAUUUUAGCAACAAAUCCAAUAAAU
Has Score: -7.895863227227556
Raw: -186.86752183827625
p-value: 0.32131859480284986
Alignment Starts at: 14 on transcript.
Alignment: 6
1'_____________SDDDDNDENNDTDDSQDDDKD__
6 _____________LGTNPNNVNNLNNGINTCNLA__
T UUUAAUACCGAUAUUUUAGCAACAAAUCCAAUAAAU
Has Score: -7.901212383243392

Raw: -186.97450495859297
p-value: 0.3361058835291343
Alignment Starts at: 2 on transcript.
Alignment: 7
1'_SDDDDNDENNDTDDSQDDDKD______________
6 _LGTNPNNVNNLNNGINTCNLA______________
T UUUAAUACCGAUAUUUUAGCAACAAAUCCAAUAAAU
Has Score: -7.981369001236933
Raw: -188.5776373184638
p-value: 0.5752571135357658
##

Protein: OTP84
Motif Locations:
[(60, 94), (95, 129), (131, 161), (162, 196), (197, 233), (234, 264), (265, 299), (300, 334), (336, 370), (367, 397), (403, 437), (438, 472), (469, 503), (515, 549), (550, 580), (581, 615), (616, 651), (652, 682), (683, 718), (719, 753), (765, 800)]
['P', 'L', 'S', 'P', 'L', 'S', 'P', 'L', 'S', 'P', 'L', 'S', 'P', 'L', 'S', 'P', 'P']
Motif Binding Pairs:
['D:IP', 'S:PL', 'N:NS', 'S:NP', 'N:VL', 'D:NS', 'D:NP', 'N:SL', 'K:SS', 'N:NP', 'D:AL', 'D:NS', 'N:NP', 'D:ML', 'N:SS', 'N:NP', 'S:IP']
Bining Protein to Target: **ndhB-1481**
Alignment Starts at: 5 on transcript.
Alignment: 1
1'____DSNSNDDNKNDDNDNNS_______________
6 ____IPNNVNNSSNANNMSNI_______________
T GUAUGUGUGAUAGCAUCUACUAUACCAGGAAUAUCA
Has Score: -7.339391755865076
Raw: -152.3971300091891
p-value: 0.003297018261048385
Alignment Starts at: 11 on transcript.
Alignment: 2
1'__________DSNSNDDNKNDDNDNNS_________
6 __________IPNNVNNSSNANNMSNI_________
T GUAUGUGUGAUAGCAUCUACUAUACCAGGAAUAUCA
Has Score: -7.453853694105815
Raw: -154.2285210210409
p-value: 0.020268470713414508
Alignment Starts at: 8 on transcript.
Alignment: 3
1'_______DSNSNDDNKNDDNDNNS____________
6 _______IPNNVNNSSNANNMSNI____________
T GUAUGUGUGAUAGCAUCUACUAUACCAGGAAUAUCA
Has Score: -7.632421555372234
Raw: -157.0856068013036
p-value: 0.15736360598144733
Alignment Starts at: 2 on transcript.
Alignment: 4
1'_DSNSNDDNKNDDNDNNS__________________
6 _IPNNVNNSSNANNMSNI__________________
T GUAUGUGUGAUAGCAUCUACUAUACCAGGAAUAUCA
Has Score: -7.660875041160432
Raw: -157.54086257391484
p-value: 0.20068655686317172
Alignment Starts at: 17 on transcript.
Alignment: 5
1'________________DSNSNDDNKNDDNDNNS___
6 ________________IPNNVNNSSNANNMSNI___
T GUAUGUGUGAUAGCAUCUACUAUACCAGGAAUAUCA
Has Score: -7.688243815151765
Raw: -157.978762957776
p-value: 0.2484660275519126
Alignment Starts at: 10 on transcript.
Alignment: 6
1'_________DSNSNDDNKNDDNDNNS__________
6 _________IPNNVNNSSNANNMSNI__________
T GUAUGUGUGAUAGCAUCUACUAUACCAGGAAUAUCA

Has Score: -7.739313135287283
Raw: -158.7958720799444
p-value: 0.3515815300670794
Alignment Starts at: 12 on transcript.
Alignment: 7
1'___________DSNSNDDNKNDDNDNNS________
6 ___________IPNNVNNSSNANNMSNI________
T GUAUGUGUGAUAGCAUCUACUAUACCAGGAAUAUCA
Has Score: -7.763338866469178
Raw: -159.1802837788547
p-value: 0.4048817273942754
##

Protein: Glyma.15G156600.1
Motif Locations:
[(38, 72), (73, 107), (110, 140), (141, 175), (176, 207), (211, 241), (242, 276), (277, 311), (313, 343), (344, 379), (380, 414), (415, 445), (446, 476), (499, 533), (534, 564), (565, 599), (606, 641), (642, 672), (673, 708), (709, 743), (755, 790), (861, 879)]
['P', 'L', 'S', 'P', 'L', 'S', 'P', 'L', 'S', 'P', 'L', 'S', 'P', 'L', 'S', 'P']
Motif Binding Pairs:
['D:IP', 'S:PL', 'D:NS', 'T:NP', 'R:VL', 'D:NS', 'D:NP', 'N:AL', 'T:TS', 'N:NP', 'D:AL', 'D:NS', 'N:NP', 'D:ML', 'N:SS', 'N:NP']
Bining Protein to Target: ndhB-1481
Alignment Starts at: 5 on transcript.
Alignment: 1
1'____DSDTRDDNTNDDNDNN________________
6 ____IPNNVNNATNANNMSN________________
T GUAUGUGUGAUAGCAUCUACUAUACCAGGAAUAUCA
Has Score: -7.232018997997463
Raw: -144.93581620071933
p-value: 0.009638683418593681
Alignment Starts at: 11 on transcript.
Alignment: 2
1'__________DSDTRDDNTNDDNDNN__________
6 __________IPNNVNNATNANNMSN__________
T GUAUGUGUGAUAGCAUCUACUAUACCAGGAAUAUCA
Has Score: -7.459719739084237
Raw: -148.35132731702095
p-value: 0.11765419504622665
Alignment Starts at: 2 on transcript.
Alignment: 3
1'_DSDTRDDNTNDDNDNN___________________
6 _IPNNVNNATNANNMSN___________________
T GUAUGUGUGAUAGCAUCUACUAUACCAGGAAUAUCA
Has Score: -7.487645614313591
Raw: -148.77021544546125
p-value: 0.1479324003111233
Alignment Starts at: 8 on transcript.
Alignment: 4
1'_______DSDTRDDNTNDDNDNN_____________
6 _______IPNNVNNATNANNMSN_____________
T GUAUGUGUGAUAGCAUCUACUAUACCAGGAAUAUCA
Has Score: -7.510529075887365
Raw: -149.11346736906788
p-value: 0.17633313241353166
Alignment Starts at: 17 on transcript.
Alignment: 5
1'________________DSDTRDDNTNDDNDNN____
6 ________________IPNNVNNATNANNMSN____
T GUAUGUGUGAUAGCAUCUACUAUACCAGGAAUAUCA
Has Score: -7.602440007370978
Raw: -150.49213134132208
p-value: 0.3213716304413987
Alignment Starts at: 9 on transcript.
Alignment: 6
1'________DSDTRDDNTNDDNDNN____________
6 ________IPNNVNNATNANNMSN____________

T GUAUGUGUGAUAGCAUCUACUAUACCAGGAAUAUCA
Has Score: -7.6214953987201675
Raw: -150.7779622115599
p-value: 0.35668145892666847
Alignment Starts at: 12 on transcript.
Alignment: 7
1'___________DSDTRDDNTNDDNDNN_________
6 ___________IPNNVNNATNANNMSN_________
T GUAUGUGUGAUAGCAUCUACUAUACCAGGAAUAUCA
Has Score: -7.628570893282596
Raw: -150.88409462999633
p-value: 0.37013226763201307
##

Protein: Glyma.06G206900.1
Motif Locations:
[(51, 85), (90, 120), (126, 156), (157, 192), (193, 227), (228, 262), (259, 289), (297, 331), (332, 362), (363, 393), (399, 433), (434, 464), (465, 499), (500, 534), (535, 565), (566, 600), (601, 635), (636, 666), (667, 701), (702, 737), (738, 768), (770, 800), (804, 838), (867, 901), (902, 955), (956, 974)]
['P', 'L', 'S', 'P', 'L', 'S', 'P', 'L', 'S', 'P', 'L', 'S', 'P', 'L', 'S', 'P', 'L', 'S', 'P', 'L', 'S']
Motif Binding Pairs:
['S:LP', 'D:GL', 'D:TS', 'D:NP', 'D:PL', 'N:NS', 'D:NP', 'E:VL', 'N:NS', 'N:NP', 'D:LL', 'T:NS', 'D:NP', 'D:GL', 'S:IS', 'Q:NP', 'D:TL', 'D:CS', 'D:NP', 'K:LL', 'D:AS']
Bining Protein to Target: ndhB-1481
Alignment Starts at: 5 on transcript.
Alignment: 1
1'____SDDDDNDENNDTDDSQDDDKD___________
6 ____LGTNPNNVNNLNNGINTCNLA___________
T GUAUGUGUGAUAGCAUCUACUAUACCAGGAAUAUCA
Has Score: -7.629260131941235
Raw: -181.53545993254983
p-value: 0.006167728146887199
Alignment Starts at: 14 on transcript.
Alignment: 2
1'_____________SDDDDNDENNDTDDSQDDDKD__
6 _____________LGTNPNNVNNLNNGINTCNLA__
T GUAUGUGUGAUAGCAUCUACUAUACCAGGAAUAUCA
Has Score: -7.786246323516335
Raw: -184.6751837640518
p-value: 0.096432861527804
Alignment Starts at: 3 on transcript.
Alignment: 3
1'__SDDDDNDENNDTDDSQDDDKD_____________
6 __LGTNPNNVNNLNNGINTCNLA_____________
T GUAUGUGUGAUAGCAUCUACUAUACCAGGAAUAUCA
Has Score: -7.829894094435717
Raw: -185.54813918243946
p-value: 0.16641749280435753
Alignment Starts at: 8 on transcript.
Alignment: 4
1'_______SDDDDNDENNDTDDSQDDDKD________
6 _______LGTNPNNVNNLNNGINTCNLA________
T GUAUGUGUGAUAGCAUCUACUAUACCAGGAAUAUCA
Has Score: -7.835074566227597
Raw: -185.65174861827708
p-value: 0.17649394280999103
Alignment Starts at: 11 on transcript.
Alignment: 5
1'__________SDDDDNDENNDTDDSQDDDKD_____
6 __________LGTNPNNVNNLNNGINTCNLA_____
T GUAUGUGUGAUAGCAUCUACUAUACCAGGAAUAUCA
Has Score: -7.866106590716257
Raw: -186.27238910805028
p-value: 0.24461408804884155
Alignment Starts at: 9 on transcript.
Alignment: 6
1'________SDDDDNDENNDTDDSQDDDKD_______

6 ________LGTNPNNVNNLNNGINTCNLA_______
T GUAUGUGUGAUAGCAUCUACUAUACCAGGAAUAUCA
Has Score: -7.898947499835316
Raw: -186.92920729043144
p-value: 0.32981212037820795
Alignment Starts at: 2 on transcript.
Alignment: 7
1'_SDDDDNDENNDTDDSQDDDKD______________
6 _LGTNPNNVNNLNNGINTCNLA______________
T GUAUGUGUGAUAGCAUCUACUAUACCAGGAAUAUCA
Has Score: -7.911266865332531
Raw: -187.17559460037575
p-value: 0.36458246221465335
##

Protein: Glyma.15G273200.1
Motif Locations:
[(0, 13), (14, 44), (45, 79), (80, 114), (115, 145), (146, 180), (181, 215), (216, 246), (247, 281), (282, 316), (317, 347), (348, 382), (383, 417), (418, 448), (449, 483), (484, 518), (519, 549), (550, 584), (585, 620), (621, 651), (652, 686), (687, 721)]
['S', 'S', 'P', 'L', 'S', 'P', 'L', 'S', 'P', 'L', 'S', 'P', 'L', 'S', 'P', 'L', 'S', 'P']
Motif Binding Pairs:
['D:HS', 'D:NS', 'N:SP', 'D:PL', 'N:NS', 'N:NP', 'D:SL', 'D:NS', 'N:NP', 'D:SL', 'D:VS', 'N:NP', 'D:SL', 'D:NS', 'D:TP', 'D:SL', 'G:NS', 'N:SP']
Bining Protein to Target: ndhB-1481
Alignment Starts at: 10 on transcript.
Alignment: 1
1'_________DDNDNNDDNDDNDDDDGN_________
6 _________HNSPNNSNNSVNSNTSNS_________
T GUAUGUGUGAUAGCAUCUACUAUACCAGGAAUAUCA
Has Score: -7.554787121185198
Raw: -161.9040643460101
p-value: 0.015337726747612055
Alignment Starts at: 15 on transcript.
Alignment: 2
1'______________DDNDNNDDNDDNDDDDGN____
6 ______________HNSPNNSNNSVNSNTSNS____
T GUAUGUGUGAUAGCAUCUACUAUACCAGGAAUAUCA
Has Score: -7.690614855318703
Raw: -164.21313582627974
p-value: 0.08802209811088418
Alignment Starts at: 13 on transcript.
Alignment: 3
1'____________DDNDNNDDNDDNDDDDGN______
6 ____________HNSPNNSNNSVNSNTSNS______
T GUAUGUGUGAUAGCAUCUACUAUACCAGGAAUAUCA
Has Score: -7.692152434144617
Raw: -164.23927466632026
p-value: 0.08949255719335047
Alignment Starts at: 1 on transcript.
Alignment: 4
1'DDNDNNDDNDDNDDDDGN__________________
6 HNSPNNSNNSVNSNTSNS__________________
T GUAUGUGUGAUAGCAUCUACUAUACCAGGAAUAUCA
Has Score: -7.771188834024048
Raw: -165.58289346427048
p-value: 0.19117482932746427
Alignment Starts at: 9 on transcript.
Alignment: 5
1'________DDNDNNDDNDDNDDDDGN__________
6 ________HNSPNNSNNSVNSNTSNS__________
T GUAUGUGUGAUAGCAUCUACUAUACCAGGAAUAUCA
Has Score: -7.784124815737037
Raw: -165.80280515339138
p-value: 0.21284229245947128
Alignment Starts at: 14 on transcript.
Alignment: 6

1'_____________DDNDNNDDNDDNDDDDGN_____
6 _____________HNSPNNSNNSVNSNTSNS_____
T GUAUGUGUGAUAGCAUCUACUAUACCAGGAAUAUCA
Has Score: -7.7917510385046285
Raw: -165.93245094044042
p-value: 0.22626103394737657
Alignment Starts at: 3 on transcript.
Alignment: 7
1'__DDNDNNDDNDDNDDDDGN________________
6 __HNSPNNSNNSVNSNTSNS________________
T GUAUGUGUGAUAGCAUCUACUAUACCAGGAAUAUCA
Has Score: -7.792372741525171
Raw: -165.94301989178962
p-value: 0.22737563057868115
##

Protein: OTP86
Motif Locations:
[(78, 108), (114, 144), (145, 179), (180, 214), (215, 245), (247, 281), (282, 316), (318, 348), (349, 383), (384, 418), (419, 449), (450, 484), (485, 515), (519, 549), (550, 584), (585, 619), (620, 650), (651, 685), (686, 716), (718, 752), (754, 784), (788, 822)]
['L', 'S', 'P', 'L', 'S', 'P', 'L', 'S', 'P', 'L', 'S', 'P', 'S', 'S', 'P', 'L', 'S', 'P']
Motif Binding Pairs:
['L:AL', 'T:GS', 'G:NP', 'T:PL', 'D:NS', 'N:NP', 'E:VL', 'D:NS', 'D:NP', 'N:TL', 'D:NS', 'D:TP', 'D:GS', 'D:NS', 'D:TP', 'E:LL', 'G:VS', 'D:TP']
Bining Protein to Target: **rps14-80**
Alignment Starts at: 6 on transcript.
Alignment: 1
1'_____LTGTDNEDDNDDDDDEGD_____________
6 _____AGNPNNVNNTNTGNTLVT_____________
T AAAUAUCAUUUGAUUCGUCGAUCCUCAAAAAAGGAA
Has Score: -7.685313380037142
Raw: -164.12301074649315
p-value: 0.04227963780083864
Alignment Starts at: 3 on transcript.
Alignment: 2
1'__LTGTDNEDDNDDDDDEGD________________
6 __AGNPNNVNNTNTGNTLVT________________
T AAAUAUCAUUUGAUUCGUCGAUCCUCAAAAAAGGAA
Has Score: -7.733941531400936
Raw: -164.9496893196776
p-value: 0.07328332192434031
Alignment Starts at: 1 on transcript.
Alignment: 3
1'LTGTDNEDDNDDDDDEGD__________________
6 AGNPNNVNNTNTGNTLVT__________________
T AAAUAUCAUUUGAUUCGUCGAUCCUCAAAAAAGGAA
Has Score: -7.7718693900053895
Raw: -165.5944629159533
p-value: 0.10771161379826083
Alignment Starts at: 2 on transcript.
Alignment: 4
1'_LTGTDNEDDNDDDDDEGD_________________
6 _AGNPNNVNNTNTGNTLVT_________________
T AAAUAUCAUUUGAUUCGUCGAUCCUCAAAAAAGGAA
Has Score: -7.834600047849337
Raw: -166.66088409930043
p-value: 0.18766478189795288
Alignment Starts at: 11 on transcript.
Alignment: 5
1'__________LTGTDNEDDNDDDDDEGD________
6 __________AGNPNNVNNTNTGNTLVT________
T AAAUAUCAUUUGAUUCGUCGAUCCUCAAAAAAGGAA
Has Score: -7.88557064004764
Raw: -167.52738416667165
p-value: 0.2741489267577194
Alignment Starts at: 18 on transcript.

Alignment: 6
1'_________________LTGTDNEDDNDDDDDEGD_
6 _________________AGNPNNVNNTNTGNTLVT_
T AAAUAUCAUUUGAUUCGUCGAUCCUCAAAAAAGGAA
Has Score: -7.88770991323416
Raw: -167.56375181084255
p-value: 0.2781655681006182
Alignment Starts at: 17 on transcript.
Alignment: 7
1'________________LTGTDNEDDNDDDDDEGD__
6 ________________AGNPNNVNNTNTGNTLVT__
T AAAUAUCAUUUGAUUCGUCGAUCCUCAAAAAAGGAA
Has Score: -7.911373976151329
Raw: -167.96604088043438
p-value: 0.32440532699950136
##

Protein: Glyma.02G144100.1
Motif Locations:
[(62, 92), (95, 125), (126, 160), (161, 195), (196, 227), (229, 263), (264, 298), (299, 329), (330, 364), (365, 399), (400, 430), (431, 465), (466, 496), (500, 530), (531, 565), (566, 600), (601, 631), (632, 666), (667, 697), (699, 733), (735, 765), (769, 803), (804, 921), (922, 940)]
['S', 'S', 'P', 'L', 'S', 'P', 'L', 'S', 'P', 'L', 'S', 'P', 'S', 'S', 'P', 'L', 'S', 'P', 'S', 'P', 'S']
Motif Binding Pairs:
['S:SS', 'T:TS', 'D:NP', 'F:PL', 'D:NS', 'N:NP', 'D:VL', 'D:NS', 'D:NP', 'N:LL', 'D:NS', 'D:TP', 'D:GS', 'D:NS', 'D:TP', 'E:IL', 'D:SS', 'D:TP', 'Q:LS', 'S:PP', 'N:CS']
Bining Protein to Target: rps14-80
Alignment Starts at: 1 on transcript.
Alignment: 1
1'STDFDNDDDNDDDDDEDDQSN_______________
6 STNPNNVNNLNTGNTISTLPC_______________
T AAAUAUCAUUUGAUUCGUCGAUCCUCAAAAAAGGAA
Has Score: -7.557784588787354
Raw: -180.1059490694722
p-value: 0.05245961417793013
Alignment Starts at: 6 on transcript.
Alignment: 2
1'_____STDFDNDDDNDDDDDEDDQSN__________
6 _____STNPNNVNNLNTGNTISTLPC__________
T AAAUAUCAUUUGAUUCGUCGAUCCUCAAAAAAGGAA
Has Score: -7.616164846795108
Raw: -181.27355422962728
p-value: 0.10143628928321097
Alignment Starts at: 2 on transcript.
Alignment: 3
1'_STDFDNDDDNDDDDDEDDQSN______________
6 _STNPNNVNNLNTGNTISTLPC______________
T AAAUAUCAUUUGAUUCGUCGAUCCUCAAAAAAGGAA
Has Score: -7.672373847670073
Raw: -182.39773424712658
p-value: 0.1740451886367949
Alignment Starts at: 4 on transcript.
Alignment: 4
1'___STDFDNDDDNDDDDDEDDQSN____________
6 ___STNPNNVNNLNTGNTISTLPC____________
T AAAUAUCAUUUGAUUCGUCGAUCCUCAAAAAAGGAA
Has Score: -7.702303176682163
Raw: -182.99632082736838
p-value: 0.22366809834686546
Alignment Starts at: 11 on transcript.
Alignment: 5
1'__________STDFDNDDDNDDDDDEDDQSN_____
6 __________STNPNNVNNLNTGNTISTLPC_____
T AAAUAUCAUUUGAUUCGUCGAUCCUCAAAAAAGGAA
Has Score: -7.717068799748037
Raw: -183.29163328868583
p-value: 0.25084544518371793

Alignment Starts at: 3 on transcript.
Alignment: 6
1'__STDFDNDDDNDDDDDEDDQSN_____________
6 __STNPNNVNNLNTGNTISTLPC_____________
T AAAUAUCAUUUGAUUCGUCGAUCCUCAAAAAAGGAA
Has Score: -7.726813493510326
Raw: -183.48652716393164
p-value: 0.26969549935505255
Alignment Starts at: 9 on transcript.
Alignment: 7
1'________STDFDNDDDNDDDDDEDDQSN_______
6 ________STNPNNVNNLNTGNTISTLPC_______
T AAAUAUCAUUUGAUUCGUCGAUCCUCAAAAAAGGAA
Has Score: -7.746299331067327
Raw: -183.87624391507165
p-value: 0.30939702916343714
##

Protein: Glyma.20G155800.1
Motif Locations:
[(44, 74), (77, 107), (108, 142), (143, 177), (178, 209), (211, 245), (246, 280), (281, 311), (312, 346), (347, 381), (382, 412), (413, 447), (448, 478), (482, 512), (513, 547), (548, 582), (590, 624), (625, 655), (657, 691), (693, 723), (727, 761), (762, 879), (880, 898)]
['S', 'S', 'P', 'L', 'S', 'P', 'L', 'S', 'P', 'L', 'S', 'P', 'S', 'S', 'P', 'P', 'L', 'S', 'P', 'S']
Motif Binding Pairs:
['S:SS', 'T:TS', 'D:NP', 'F:PL', 'D:NS', 'N:NP', 'D:VL', 'D:NS', 'D:NP', 'N:LL', 'D:NS', 'D:TP', 'D:GS', 'D:NS', 'D:TP', 'L:IP', 'D:AL', 'Q:LS', 'S:PP', 'N:CS']
Bining Protein to Target: rps14-80
Alignment Starts at: 2 on transcript.
Alignment: 1
1'_STDFDNDDDNDDDDDLDQSN______________
6 _STNPNNVNNLNTGNTIALPC______________
T AAAUAUCAUUUGAUUCGUCGAUCCUCAAAAAAGGAA
Has Score: -7.5844448021796085
Raw: -174.56963901693115
p-value: 0.023752891022502248
Alignment Starts at: 6 on transcript.
Alignment: 2
1'_____STDFDNDDDNDDDDDLDQSN__________
6 _____STNPNNVNNLNTGNTIALPC__________
T AAAUAUCAUUUGAUUCGUCGAUCCUCAAAAAAGGAA
Has Score: -7.598240293949114
Raw: -174.83175336055174
p-value: 0.02894708652182162
Alignment Starts at: 9 on transcript.
Alignment: 3
1'________STDFDNDDDNDDDDDLDQSN_______
6 ________STNPNNVNNLNTGNTIALPC_______
T AAAUAUCAUUUGAUUCGUCGAUCCUCAAAAAAGGAA
Has Score: -7.73522396160408
Raw: -177.43444304599612
p-value: 0.14689802655642592
Alignment Starts at: 1 on transcript.
Alignment: 4
1'STDFDNDDDNDDDDDLDQSN_______________
6 STNPNNVNNLNTGNTIALPC_______________
T AAAUAUCAUUUGAUUCGUCGAUCCUCAAAAAAGGAA
Has Score: -7.748648006084907
Raw: -177.68949989113182
p-value: 0.16680770010336543
Alignment Starts at: 11 on transcript.
Alignment: 5
1'__________STDFDNDDDNDDDDDLDQSN_____
6 __________STNPNNVNNLNTGNTIALPC_____
T AAAUAUCAUUUGAUUCGUCGAUCCUCAAAAAAGGAA
Has Score: -7.762276680671571
Raw: -177.94844470827843

p-value: 0.18872109810297827
Alignment Starts at: 14 on transcript.
Alignment: 6
1'_____________STDFDNDDDNDDDDDLDQSN___
6 _____________STNPNNVNNLNTGNTIALPC___
T AAAUAUCAUUUGAUUCGUCGAUCCUCAAAAAAGGAA
Has Score: -7.845541887947674
Raw: -179.5304836465244
p-value: 0.3564532561886786
Alignment Starts at: 8 on transcript.
Alignment: 7
1'_______STDFDNDDDNDDDDDLDQSN_________
6 _______STNPNNVNNLNTGNTIALPC_________
T AAAUAUCAUUUGAUUCGUCGAUCCUCAAAAAAGGAA
Has Score: -7.847830578049767
Raw: -179.57396875846416
p-value: 0.36174106586110366
##

Protein: Glyma.15G273200.1
Motif Locations:
[(0, 13), (14, 44), (45, 79), (80, 114), (115, 145), (146, 180), (181, 215), (216, 246), (247, 281), (282, 316), (317, 347), (348, 382), (383, 417), (418, 448), (449, 483), (484, 518), (519, 549), (550, 584), (585, 620), (621, 651), (652, 686), (687, 721)]
['S', 'S', 'P', 'L', 'S', 'P', 'L', 'S', 'P', 'L', 'S', 'P', 'L', 'S', 'P', 'L', 'S', 'P']
Motif Binding Pairs:
['D:HS', 'D:NS', 'N:SP', 'D:PL', 'N:NS', 'N:NP', 'D:SL', 'D:NS', 'N:NP', 'D:SL', 'D:VS', 'N:NP', 'D:SL', 'D:NS', 'D:TP', 'D:SL', 'G:NS', 'N:SP']
Bining Protein to Target: rps14-80
Alignment Starts at: 3 on transcript.
Alignment: 1
1'__DDNDNNDDNDDNDDDDGN________________
6 __HNSPNNSNNSVNSNTSNS________________
T AAAUAUCAUUUGAUUCGUCGAUCCUCAAAAAAGGAA
Has Score: -7.573519693603934
Raw: -162.2225180771286
p-value: 0.02019221534820082
Alignment Starts at: 11 on transcript.
Alignment: 2
1'__________DDNDNNDDNDDNDDDDGN________
6 __________HNSPNNSNNSVNSNTSNS________
T AAAUAUCAUUUGAUUCGUCGAUCCUCAAAAAAGGAA
Has Score: -7.586386185020111
Raw: -162.44124843120363
p-value: 0.024234317148054154
Alignment Starts at: 14 on transcript.
Alignment: 3
1'_____________DDNDNNDDNDDNDDDDGN_____
6 _____________HNSPNNSNNSVNSNTSNS_____
T AAAUAUCAUUUGAUUCGUCGAUCCUCAAAAAAGGAA
Has Score: -7.735268099339392
Raw: -164.97224097463143
p-value: 0.13844659502331852
Alignment Starts at: 13 on transcript.
Alignment: 4
1'____________DDNDNNDDNDDNDDDDGN______
6 ____________HNSPNNSNNSVNSNTSNS______
T AAAUAUCAUUUGAUUCGUCGAUCCUCAAAAAAGGAA
Has Score: -7.744779997550642
Raw: -165.13394324422268
p-value: 0.15133519952215058
Alignment Starts at: 6 on transcript.
Alignment: 5
1'_____DDNDNNDDNDDNDDDDGN_____________
6 _____HNSPNNSNNSVNSNTSNS_____________
T AAAUAUCAUUUGAUUCGUCGAUCCUCAAAAAAGGAA

Has Score: -7.776722158085169
Raw: -165.67695997330958
p-value: 0.20027246204133986
Alignment Starts at: 8 on transcript.
Alignment: 6
1'_______DDNDNNDDNDDNDDDDGN___________
6 _______HNSPNNSNNSVNSNTSNS___________
T AAAUAUCAUUUGAUUCGUCGAUCCUCAAAAAAGGAA
Has Score: -7.7776150764495355
Raw: -165.69213958550384
p-value: 0.20176455965453188
Alignment Starts at: 18 on transcript.
Alignment: 7
1'_________________DDNDNNDDNDDNDDDDGN_
6 _________________HNSPNNSNNSVNSNTSNS_
T AAAUAUCAUUUGAUUCGUCGAUCCUCAAAAAAGGAA
Has Score: -7.792989474339794
Raw: -165.95350434963828
p-value: 0.2284843664299857
#################################
